# Identification and analysis of mutational hotspots in oncogenes and tumour suppressors

**DOI:** 10.18632/oncotarget.15514

**Published:** 2017-02-19

**Authors:** Hanadi Baeissa, Graeme Benstead-Hume, Christopher J. Richardson, Frances M.G Pearl

**Affiliations:** ^1^ School of Life Sciences, University of Sussex, Falmer, Brighton, UK; ^2^ Division of Structural Biology, The Institute of Cancer Research, London, UK

**Keywords:** cancer, mutation, oncogene, tumour suppressor

## Abstract

**Background:**

The key to interpreting the contribution of a disease-associated mutation in the development and progression of cancer is an understanding of the consequences of that mutation both on the function of the affected protein and on the pathways in which that protein is involved. Protein domains encapsulate function and position-specific domain based analysis of mutations have been shown to help elucidate their phenotypes.

**Results:**

In this paper we examine the domain biases in oncogenes and tumour suppressors, and find that their domain compositions substantially differ. Using data from over 30 different cancers from whole-exome sequencing cancer genomic projects we mapped over one million mutations to their respective Pfam domains to identify which domains are enriched in any of three different classes of mutation; missense, indels or truncations. Next, we identified the mutational hotspots within domain families by mapping small mutations to equivalent positions in multiple sequence alignments of protein domains We find that gain of function mutations from oncogenes and loss of function mutations from tumour suppressors are normally found in different domain families and when observed in the same domain families, hotspot mutations are located at different positions within the multiple sequence alignment of the domain.

**Conclusions:**

By considering hotspots in tumour suppressors and oncogenes independently, we find that there are different specific positions within domain families that are particularly suited to accommodate either a loss or a gain of function mutation. The position is also dependent on the class of mutation. We find rare mutations co-located with well-known functional mutation hotspots, in members of homologous domain superfamilies, and we detect novel mutation hotspots in domain families previously unconnected with cancer. The results of this analysis can be accessed through the MOKCa database (http://strubiol.icr.ac.uk/extra/MOKCa).

## INTRODUCTION

All cancers depend on mutations in critical genes that confer a selective advantage to the tumour cell. Knowledge of these mutations is fundamental to understanding the biology of cancer initiation and progression, and to the development of targeted therapeutic strategies. The genes that harbour the driver mutations that contribute to the disease process are traditionally classified as either as ‘tumour suppressors’ or as oncogenes, dependent on their role in cancer development.

When mutations (or epigenetic silencing) of the protein products of tumour suppressors result in their loss of function (LOF), cancer progression occurs. Driver alterations in these genes are typically molecularly recessive in nature, with both copies of the gene requiring a LOF defect. In oncogenes, an increase in activity, or a change of function is required for tumorigenesis. These genes tend to exhibit a molecularly dominant mode of action, and usually only one faulty copy of the gene is required to provide an oncogenic phenotype [1].

When mutations from cohorts of patients are sequenced and the alterations mapped to a single genome, the mutational spectra in tumour suppressors and oncogenes tend to differ. In tumour suppressors small mutations are often liberally dispersed along the length of the gene. This is because the protein products can be disrupted with damaging mutations at a multitude of positions [2, 3]. Driver missense mutations within a tumour suppressor can result in its loss of function in a variety of ways, including loss of stability of the protein or the disruption of a crucial ligand/DNA/protein-interaction site. Conversely, in oncogenes often only a very few, specific mutations in specific locations can lead to activation of the protein product or a change of protein function. Driver missense mutations consequently tend to cluster at distinct locations within a protein [4, 5], impacting on functional sites such as ligand-binding, protein-protein interactions, allosteric regulation and post-translational modifications.

Several groups have used the differences in these mutational patterns to automatically distinguish between tumour suppressor and oncogenes [6]. For instance, Vogelstein's 20:20 rule [2] can be applied to cohorts of tumour samples. Within a cohort: if 20% of all mutations observed within a gene are truncations, then the gene is likely to be a tumour suppressor. Similarly, if 20% of all missense mutations occur at a single position in the sequence, the gene is predicted to be an oncogene.

As well as discriminating between tumour suppressors and oncogenes, there are several approaches to detect which genes are likely to be drivers, irrespective of their biological function: Statistical methods have been successfully applied to identify recurrently mutated genes within large cohorts of sequenced tumours (eg [7, 8]). However, the data sets are not yet large enough to have the statistical power to detect low frequency mutated genes that contribute to the disease process. This poses a problem as most somatic mutations in tumours occur in genes that are rarely mutated [9, 10].

An alternative approach to identifying drivers uses sequence and structural data to predict whether a missense mutation, or small insertion/deletion (indel) could contribute to disease by impacting on the function of the encoded protein [11, 12]. Sequence conservation is used to predict which mutations can be tolerated within a protein structure, and protein structures have been used for estimating how disruptive a missense mutation might be [13]. More recently algorithms have been specifically developed to distinguish cancer-associated somatic driver missense mutations from passenger mutations. These include profile-based methods for assessing missense mutations (eg FATHHM [14], Mutation assessor [15], TransFIC [16]), and machine learning algorithms for assessing the pathogenicity of missense mutations (eg Inca [17], CHASM [18]) and indels [19].

While most analysis of cancer mutations has been gene-centric, considering encoded proteins as a whole, a few studies have focused on the individual protein domains affected [20–22]. Larger proteins are often comprised of sets of recognizable domains that recur in other proteins in various combinations [23]. These domains may be thought of as units of evolution, creating protein domain families, which share a ‘common ancestor’. A domain can exist across multiple proteins with conserved function and structure, it follows that similarly located mutations across different proteins in the same domain should have similar effects on the function of that domain. A well-documented example of this is the activating V600E mutation in the kinase domain of BRAF [24], which is found in thyroid cancer and malignant melanoma. Comparable activating mutations occur at the equivalent position in the kinase domain of c-KIT (D816V) in gastrointestinal stromal tumours (GIST) and acute myeloid leukaemia (AML), and in the kinase domain of FLT3 (D835Y) in AML [4, 25]. Similarly, KRAS, NRAS, HRAS all have highly recurrent activating mutations at position G12 (KRAS) in the Ras domain in a large variety of cancers [4, 21].

Proteome-wide analyses have previously been performed to identify domains enriched in missense mutations [20, 21, 26, 27] and to identify hotspot positions in missense mutations [5, 22, 28–30]. In these studies all missense mutations were analysed concurrently rather than segregated into those that would likely result in a loss of function and for those that would result in a gain.

Here we examine the domain biases in oncogenes and tumour suppressors, and have also compared them with genes not assigned to these roles and find that their domain compositions substantially differ. We have mapped over 1 million mutations from whole-exome sequencing cancer genomic projects including data from over 30 different types of cancer and identified which domains are recurrently mutated in tumour suppressors, oncogenes and throughout the genome. We have divided the mutations into three different classes; missense, truncations or indels. Finally we identified the mutational hotspots within domain families by mapping small mutations to equivalent positions in multiple sequence alignments of protein domains. Examining the differences in the distribution of the positions of domain hotspots, between tumour suppressors and oncogenes, has enabled us to identify key positions of activating mutations in a variety of domain types. This has enabled us to identify putative gain of function mutations in proteins previously unassociated with cancer that may be actionable with current therapies. The results of this analysis can be accessed through the MOKCa database (Mutations, Oncogenes and Knowledge in Cancer,http://strubiol.icr.ac.uk/extra/MOKCa).

## RESULTS AND DISCUSSION

### Functional characterisation of tumour suppressors and oncogenes

Using the Cancer Gene Census classification we assigned 133 molecularly recessive genes as tumour suppressors and 481 molecularly dominant genes as oncogenes. Genes that were labelled as both molecularly dominant and recessive were included in both data sets.

First we analysed the biological pathways. Pathway enrichment analysis showed that tumour suppressors and oncogenes usually cluster in different molecular pathways. We found 79 pathways enriched with tumour suppressors, notably those involved in the cell cycle, response to cellular stresses and the DNA damage response. The 306 pathways enriched in oncogenes include those involved in the regulation of biosynthetic process, regulation of transcription and those involved in protein amino acid phosphorylation. Only 14 pathways were enriched in tumour suppressors and oncogenes. These included immune system development, regulation of macromolecule metabolic process, and regulation of cell proliferation and apoptosis.

Although generally segregating onto different pathways, the functions of the large majority of the proteins in oncogenes and tumour suppressors were somewhat similar (see Supplementary Figure 1), with the largest class of proteins being enzymes, (TS: 32% OG: 18%), transcription factors (TS: 11%, OG: 21%) and nucleic acid binding proteins (TS: 32%, OG: 24%) with tumour suppressor comprising of significantly more enzymes (*P* = 0.000082) and oncogenes of more transcription factors (*P* = 0.0023).

### Domain characterisation of tumour suppressors and oncogenes

Next we analysed the domain compositions within tumour suppressors and oncogenes. In total 5523 Pfam domain families were identified within the 17537 proteins analysed. Tumour suppressor proteins contained 197 different types of Pfam domains with the most frequently observed domains including Helicase_C (7), DEAD (4), SET (4), HMG-box (3), F-box-like (3), ARID (3), and PHD finger (zf-HC5HC2H, 3) domains and the C-terminal domain from DNA mismatch repair proteins (DNA_mis_repair, 3). Of the 310 Pfam domain types found in our set of oncogenes the most frequently observed were Pkinase_Tyr (26), Homeobox (16), HLH (14), Ets (9), and SH2 (9) domains.

**Figure 1:**
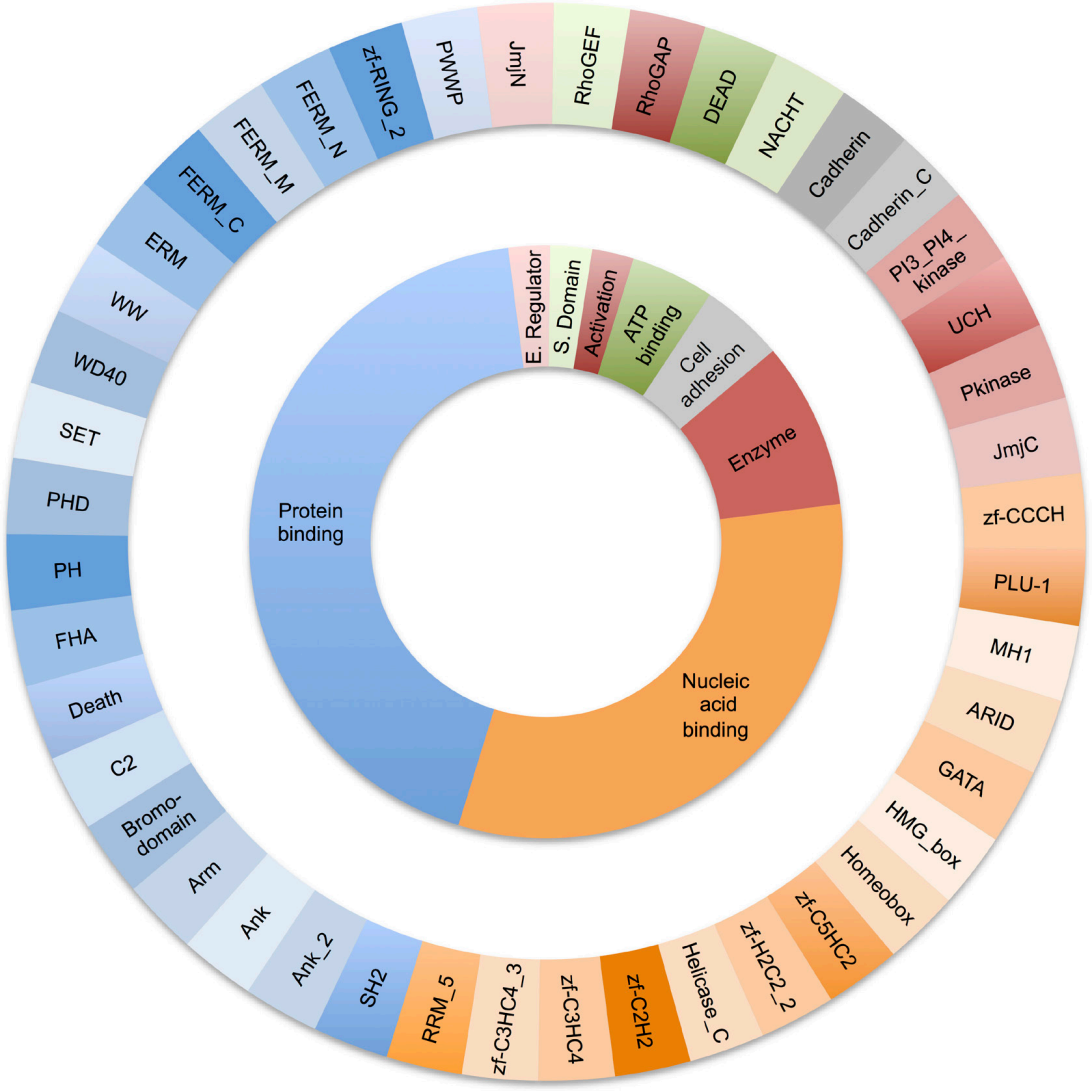
Distribution of molecular function for the 44 domains types found in both oncogenes and tumour suppressors The outer ring shows each Pfam domain type. The inner ring groups the Pfam domains by function.

We only found 44 domain types common to tumour suppressor and oncogenes. The majority of these were either protein binding modules (Ank, WD40, C2, PHD and SET domains) or modules evolved to bind to nucleic acids (Homeobox, ARID, zf-C2H2, MH1 domains, see Figure 1). Despite a substantial number of catalytic domains occurring in in TS and OG, only 5 enzyme types were common to both; the serine/threonine (Pkinase) and tyrosine protein kinases (Pkinase_Tyr), phosphatidylinositide 3-kinases (PI3_PI4_kinase), ubiquitin carboxyl-terminal hydrolase (UCH) and the JmjC protein hydroxylase.

### Identifying tumour suppressors and oncogenes using domain biases

As the domain compositions between these cancer genes differed substantially, we decided to investigate whether a gene could be classified as a tumour suppressor or an oncogene based on their domain composition alone, using a machine learning approach. Our training set comprised a list of oncogenes and a list of tumour suppressors derived from the Cancer Gene Census (CGC). Using a support vector machine classifier and a 10-fold cross validation protocol, we achieved a ROC AUC sore of 0.72 (see Supplementary Methods) suggesting that the classifier has some predictive value.

We ran the classifier on 37 genes labelled as both oncogene and tumour suppressor in the CGC. We found that 17 of the genes were predicted to be tumour suppressors with probabilities greater than 0.78, including DDB2, TP53 and DAXX. Nine genes were classified as oncogenes with probabilities greater than 0.83, including ERBB4, BCL10 and BTK. We could not resolve the classification of 11 genes using this approach (see Supplementary Table 1).

Although this classification approach may give a guide to the gene's predominant cancer role within the cell, there is increasing evidence in the literature that depending on cell type and cancer type, many genes can function as both a tumour suppressor and as an oncogene dependent on the alteration in question.

### Mutational characterisation of domains in tumour suppressors and oncogenes

To define the mutational ‘load’ that the different domain types are subjected to in cancers, we mapped mutations from Cosmic v71 (WGS) whole genome sequencing cancer studies onto the Pfam domains identified above. Mutations were grouped into three subsets; missense, truncating (nonsense or frameshift), and indels (inframe insertion and deletions). In total, 727,525 missense, 69,414 truncation and 2,958 indel mutations from over 30 different types of cancer were mapped to Pfam domains within the human genome.

### Mutational enrichment in tumour suppressors

**Figure 2:**
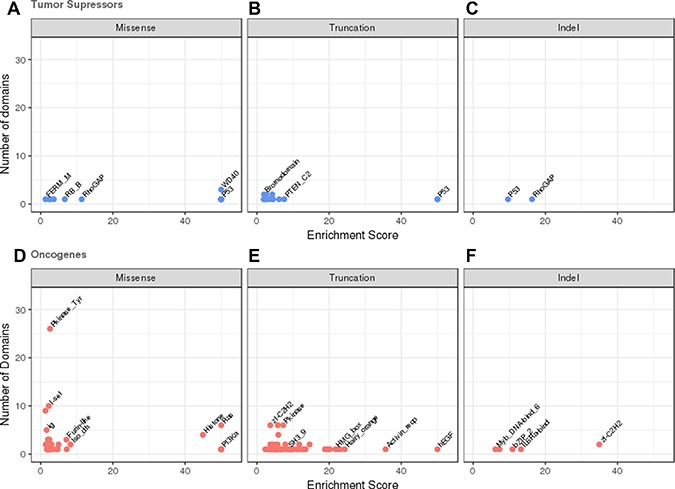
Domains enriched in mutations in oncogenes and tumour suppressors The number of domains in the dataset is plotted against the estimated mutational enrichment for that domain. Only domains with significant mutational enrichment (see methods) are shown. Missense, truncation and indel mutational enrichments are calculated independently for tumour suppressors and oncogenes. Enrichments in tumour suppressors are coloured in blue, those found in oncogenes in red. (**A**) Missense mutations in tumour suppressors, (**B**) truncation mutations in tumour suppressors (**C**) indel mutations in tumour suppressors, (**D**) missense mutations in oncogenes, (**E**) truncation mutations in oncogenes, (**F**) indel mutations in oncogenes.

The most frequently reported mutational event that changes the protein product of tumour suppressors (62%) is the missense substitution. However, only 15 domain families were significantly enriched in missense mutations (see Figure 2A and Supplementary Table 2). The majority of these were from single members of a domain family, observed within one of the frequently mutated and very well studied tumour suppressor genes. These included the P53 DNA binding domain (P53) in TP53, the dual specificity phosphatase catalytic domain (DSPc) in PTEN and the von Hippel-Lindau disease tumour suppressor protein domain (VHL) in VHL. Single amino acids substitutions usually destabilise a protein fold [31–32], and wild-type TP53, PTEN and VHL are only marginally stable at physiological temperatures [33–35], which make them particularly sensitive to missense mutations. Only WD40 domains had multiple members affected with mutations found in DDB2, FBXW7 and TBL1XR1.

15 domains found in tumour suppressors were enriched in truncations, again many being singleton domains from the commonly mutated major tumour suppressors where a truncation wipes out the complete function of the protein. These included domains from the protein products of in TP53, VHL, PTEN, RB1 and APC. Several domain families including WD40, Bromodomain and F-box-like domains displayed truncations in multiple members. Only 2 tumour suppressor domains were enriched with indels; RhoGAP (PIK3R1) and P53 (TP53) each from a single protein (see Figure 2B and 2C and Supplementary Tables 3 and 4).

### Mutational enrichment in oncogenes

Amino acid changes due to missense mutation are also the most frequently reported mutational event in oncogenes (85% of all reported mutations). We detected 37 domains from our set of oncogenes that were significantly enriched in missense mutations (see Figure 2D and Supplementary Table 5). These include the classic oncogene tyrosine kinase (Pkinase_Tyr) domain, the Ras domain and the isocitrate dehydrogenase domain family (Iso_dh), where multiple members of these domain families are known to contain highly recurrent gain/change of function activating missense mutations.

Single genes with significantly high densities of missense mutations included PIK3CA where the phosphatidylinositide 3-kinase, the gamma adapter protein p101 subunit and the accessory domains are all enriched in mutations. Mutations in these domains are thought to facilitate allosteric motions that stimulate lipid kinase activity required for catalysis on membranes [36]. The zinc finger domain (zf-CCCH) in U2AF1 was also enriched in mutations. U2AF1, a U2 auxiliary factor protein, recognises the AG splice acceptor dinucleotide at the 3′ end of introns. Mutations in its zinc finger domains have been found to promote enhanced splicing and exon skipping in reporter assays *in vitro* and may have a similar effect *in vivo*[37].

Domains that were mutated in more than one gene included both furin-like domains which are involved in cellular signaling, and immunoglobulin I-set domains which are involved in cellular communication. Missense mutations in these domains have been shown to disrupt protein interaction surfaces, causing disregulation and activation of these processes.

Of the 57 domains in oncogenes enriched in truncations the majority are derived from a single protein (see Figure 2E and Supplementary Table 6). They also tend to be present in oncogenes activated via a translocation into a fusion protein. It is not clear whether these truncations are actually miscalls, and are actually translocations that have not been identified by the analysis software or whether these truncations could cause activation of the protein by removal of a regulatory or binding domain. Alternatively, it may be that when not part of a fusion protein the proteins containing these domains behave as tumour suppressors rather than oncogenes. Examples of domains frequently truncated domains include the DNA-binding zinc finger (zf-H2C2_2) domains in BCL11A, BCL6, PLAG1, ZBTB16, ZNF278 and ZNF331. The protein products of these genes are thought to repress transcription so disrupting the DNA binding domains may result in the expression of different subsets of target genes. Again the sparsity of indel data (see Figure 2F and Supplementary Table 7) resulted in only 5 domains being identified as mutationally enriched, zf-C2H2, IL6Ra-bind, bZIP_2, PI3K_p85B and Myb_DNA-bind_6.

### Genome-wide mutational enrichment

We compared the domains observed in tumour suppressors and oncogenes with those enriched in mutations within the whole genome to see if we could identify novel domain families not previously associated with annotated cancer driver genes. In total, we detected 373 domains that were significantly enriched in missense mutations, of which 340 were not present in our tumour suppressor and oncogene datasets (see Supplementary Table 8). This suggests that the cancer community may be missing mutated genes that contribute to cancer progression but may not be the typical cancer genes analysed.

For example, we observed enrichment in mutations in the sushi domain also known as known as complement control protein (CCP) modules. These are small beta-sandwiches and function in proteins that are part of the innate immune system. Several sushi containing proteins have been implicated in the development of tumour cells and their loss correlates with poor prognosis [38, 39].

Similarly, in the 225 domains showing enrichment in truncations, 196 were not present in the current cancer gene set documented in the Cancer Gene Census (see Supplementary Table 9). Sushi domains were also significantly enriched in truncation mutations suggesting that the phenotypic role of the missense mutations may be loss of function mutations.

Of the 38 domains significantly enriched in indels, 31 were not present in our cancer gene lists (Supplementary Table 10).

### Detecting domain hotspots

**Figure 3:**
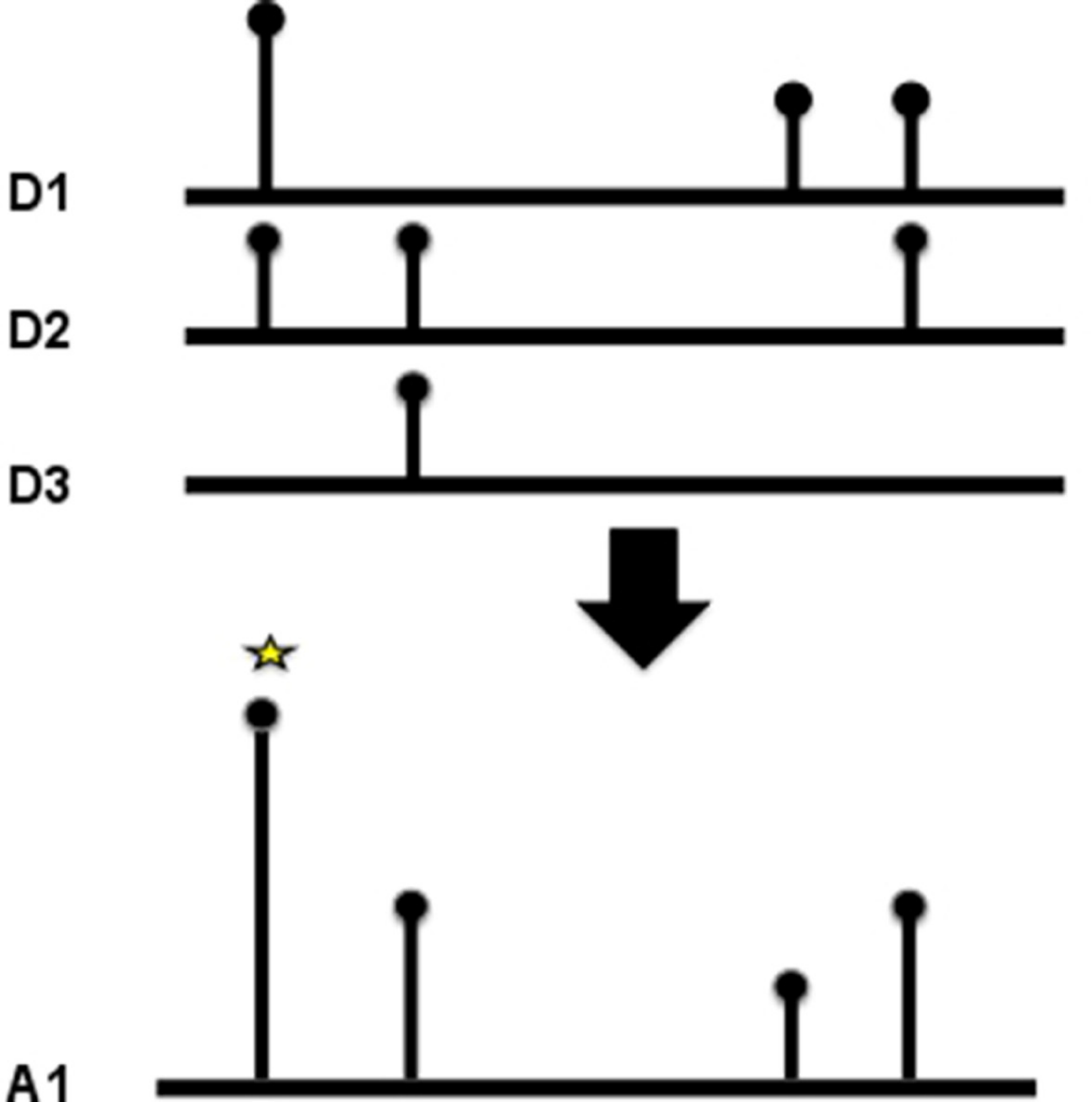
Domain hotspots To calculate a domain hotspot all the members of the domain family were aligned using MUSCLE. The position of the mutation was mapped to the multiple sequence alignment, and the number of mutations at that position summed. For the position to be considered a hotspot, at least two mutations of the same class (missense, truncation or indel) had to be recorded at the same position.

As well as identifying which domain families were enriched in mutations, we also wanted to identify the key positions within a domain, that when mutated, were particularly suited to causing a loss or change in function of the protein the domain occurs in. To achieve this we created multiple sequence alignments for each domain family and counted the mutations at each position in the alignment (see Figure 3). A binomial test was applied to determine which positions had accrued a significant number of mutations. Again we analysed tumour suppressors and oncogenes, and the different mutation types independently (see Table 1).

**Table 1:** This table describes the number of recorded and significant mutational hotspots identified in each datasets; tumour suppressor, oncogene and whole genome

Gene Type	Mutation type	#Hotspots	#Significant
Tumour suppressors	Missense	3720	119
	Indels	105	11
	Truncations	1206	73
Oncogenes	Missense	7195	85
	Indels	63	10
	Truncations	1121	42
Whole genome	Missense	65491	954
	Indels	1006	113
	Truncations	27620	506

Missense, indel and truncation mutations were analysed independently.

### Hotspot mutations in tumour suppressors

Within the annotated tumour suppressors we identified 119 missense hotspots within 42 domain families, 11 indel hotspots within 7 domain families and 73 truncation hotspots in 39 domain families (See Supplementary Tables 11, 12, 13). The positions of the hotspots were dependent on the type of mutation with little overlap in the positions of mutations between the different types of mutational alterations (see Supplementary Figure 3A).

The mutational burden of several of the hotspots was accrued from a single gene, in particular those found in TP53 and VHL. Others were derived from multiple tumour suppressor domain family members including the Pkinase and WD40 domains. Missense mutations in the protein kinase domains from CHEK2 (K373E) and MAP2K4 (G252R) have mutations co-located with the CDK12 R882L/Q mutations. The CDK12 R882L mutation has been shown to impair kinase activity, possibly by breaking critical interactions in the active conformation of the kinase between phosphorylated threonine 893 and the activation loop [40], CHEK2 K373E has been implicated as a LOF mutation leading to hereditary cancer predisposition syndrome. For these two mutations there is evidence that they result in a loss of kinase activity, suggesting that the mutations occur at a critical position in the protein structure when the kinase is in its active conformation; the co-located G252R mutation in MAP2K4 may also result in a LOF.

**Figure 4:**
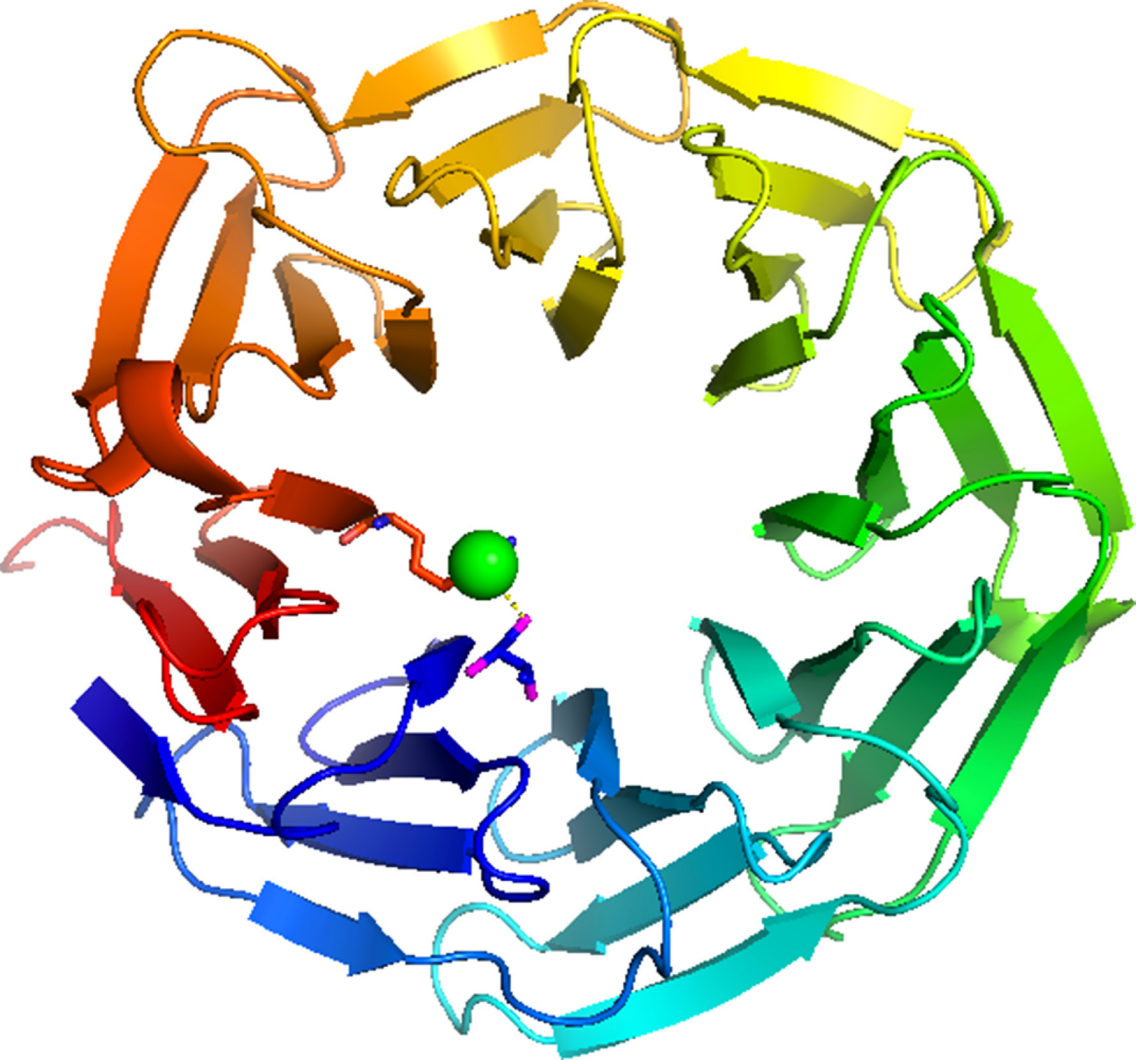
WD40 domain This illustrates the WD40 domain of FBXW7. Threonine 385 is located on the first propeller blade of the WD40, (shown in blue) forming a hydrogen bond with arginine 674 in the final propeller blade (shown in red) via a water molecule (shown as a green ball) helping to stabilise the propeller structure. Replacing the side chain with arginine would mean this hydrogen bond could not be formed destabilisation of the internal water structure of the WD40 and hence the overall fold.

Co-located mutations in the WD40 tumour suppressors FBXW7 (T385K) and TBL1XR1 (Y395H) are also likely to be loss of function. The WD40 domain is especially sensitive to position specific disruption by missense mutations because the way in which its fold is stabilized. WD40 domains consist of a β-propeller structure containing between six to eight propeller ‘blades’. These blades are each formed by a four-stranded antiparallel β-sheet, which are joined by β-hairpins. The blades are arranged symmetrically about a central axis, and the inside edge of each propellers comprise side chains that form a network of hydrogen bonds with each other, and internal water molecules that maintain the domain's stability (see Figure 4). Mutating any residue that contributes to stabilisation of this central core could be catastrophic to the overall fold. In FBXW7, threonine 385 is located on the first propeller blade of the WD40, forming a hydrogen bond with arginine 674 via a water molecule sealing the propeller structure. The replacement side chain would be unable to maintain this hydrogen bond causing destabilisation of the internal water structure and hence the overall fold.

Co-located hotspot mutations were also observed in the SNF2 family N-terminal domain (SMARC4;T1747K and ATRX;T910M) and the Helicase_C domains (ERRC3;R645Q, ATRX;R2153C, SMARCA4; R1192H/G/C).

The sparsity of both truncation and indel data meant that almost all the tumour suppressor hotspots were derived from single proteins. Truncation hotspots were observed in VHL and P53, in the RhoGAP domain in PIK3R1, and the RB_A domain in retinoblastoma associated protein. Several protein kinase domains had truncating mutations at position 14 in the domain multiple sequence alignment which would result in complete loss of function of the kinase in BUB1B (E813*), MAP3K1 (Q1247fs*26) and STK11 (D53fs*11).

TP53 exhibited the most indel hotspots with hotpots observed in DNA binding domains (P53) and the P53_tetramer tetramerisation motif. In several cases multiple variants were observed at the same hotspot. This included the P53 domain where there was a deletion of residue 113 F or several residues FLH, and at position 155 there was an insertion of DSTPPPGT and a deletion of residues TR recorded.

### Hotspots in oncogenes

Within oncogenes we identified 85 missense hotspot in 46 domain families, 10 indel hotspots within 9 domains and 42 truncation hotspots in 30 domain families (see Supplementary Tables 11, 12, 13). Again, the hotspots were category dependent with only 5 positions of mutations in common between the different mutational alterations (see Supplementary Figure 3B). Far fewer hotspots were observed per domain than in the case for tumour suppressors, which supports the conjecture there are only certain positions in a domain where a mutation can lead to the gain of function or activation that is typically found in oncogenes.

We observed the well known, high frequency mutations in the Ras (KRAS, HRAS, NRAS), isocitrate dehydrogenase (IDH1, IDH2) and tyrosine protein kinase domains (BRAF V600E etc). These highly recurrent mutations have been extensively analysed and are thought to cause a gain/change of function of the protein by changing the canonical conformation of the protein.

The small GTPases (K-RAS, N-RAS and H-RAS) are molecular switches cycling between the GTP-bound active and GDP-bound inactive conformations. They have co-located hotspots that are implicated in a large variety of cancers. When mutated at position 12, the bulky side chain of the mutants are thought to lower the GTPase activity through a steric interference of the catalytic process [41]. This leads to stabilisation of the active conformation leading to constitutive activation of downstream effectors such as phosphoinositide 3-kinases and Raf kinases.

IDH1 and IDH2 catalyse the oxidative carboxylation of isocitrate to α-ketoglutarate. Mutational hotspots at R132H in IDH1, and R140Q and R172K in IDH2 alter the progression of this reaction. Recent structural work suggests that the R132H IDH1 mutation hampers the conformational change from the initial isocitrate binding state to the pre-transition state, thus causing an impairment of enzyme function [42]. This alters the progression of this reaction causing the oncometabolite R(-)-2-hydroxyglutarate to be formed. R(-)-2-hydroxyglutarate is implicated in genomic hypermethylation, leading to histone methylation, genomic instability, and finally malignant transformation [43].

Other less will documented co-located missense hotspot mutations were found in the guanine nucleotide binding protein domains (G_alpha). GNAS R201H somatic mutation is an activating mutation resulting in constitutively activated G-alpha protein and the downstream cAMP cascade, independent of TSH signalling [44]. This results in the autonomously functioning thyroid nodules. The co-located with activating R183 mutations observed in GNA11 and GNAQ in uveal melanoma [56].

In the rhodopsin seven transmembrane helix domain family the (7tm_1) the thyrotropin receptor (TSHR) A623V activating mutations [45] are co-located with R251 mutations from the atypical chemokine receptor 3 (ACKR3). Other domain families with co-located missense mutations include the trypsin, 14-3-3, sema, frizzled, yeats and jun domain families.

Few of the truncation hotspots in oncogenes were observed in more than one protein, suggesting that truncating mutations, if they result in a consequence, may be specific to the context of the domain within the larger protein, rather than to the domain itself.

Although the indel data was sparse there was still some evidence that co-located indel hotspot mutations in oncogenes are activating. Co-located deletions E746_A750delELREA and E746_T751delELREAT both cause activation of EGFR [46], and are also co-located deletion in BRAF (M484_N486delMLN) (see Supplementary Tables 11, 12, 13).

### Hotspots in tumour suppressors and oncogenes occur in different positions in the domains

**Figure 5:**
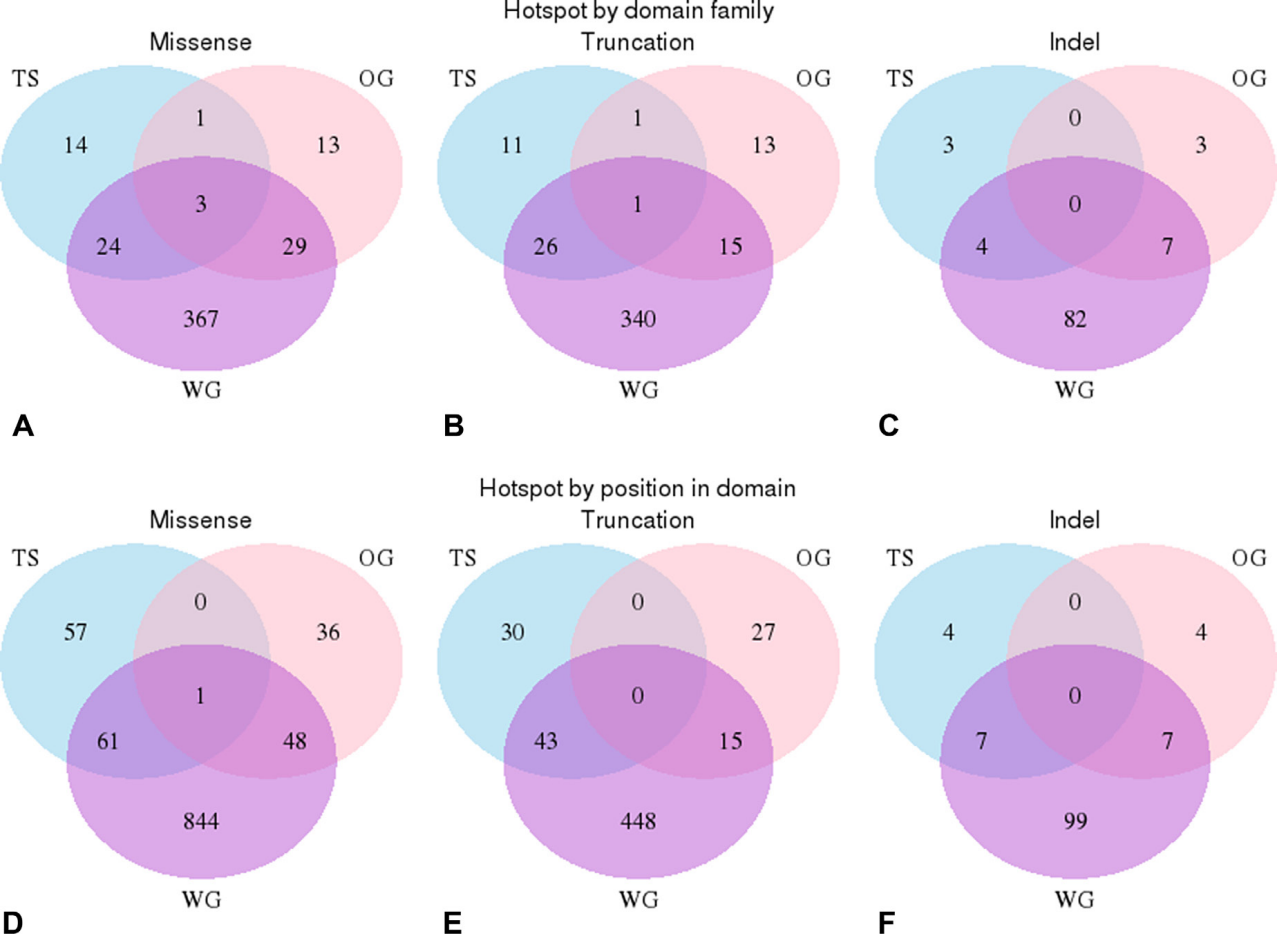
Positional analysis of domain hotspots Analysis of the overlap in the positions of the significant hotspots in oncogenes and tumour suppressors compared with those found within the whole genome. (**A**–**C**) These venn diagrams illustrate that significant hotspots can occur in the same domain family in oncogenes (pink), tumour suppressors (blue) and in the whole genome (purple). Each circle represents the number of domains that contains a hotspot mutation, intersections illustrate when the same domain is found in more than one data set. (A) missense mutations (B) truncation mutations and (C) indels mutations. (**D**–**F**) These venn diagrams illustrate that significant hotspots that occur in the same position in domain families in oncogenes, tumour suppressors and within the whole genome; (D) missense mutations, (E) truncations (F) indels mutations.

In total we identified 341 mutational hotspots within 66 domains in our cancer gene set. The hotspots in tumour suppressors and oncogenes occurred in different domain types except in 6 domains ( Pkinase, SET, Pkinase_Tyr, Tet_JBP, PI3_PI4_kinase, RhoGAP) and when they were observed in the same domain type, they were found with in different locations in the domain (see Figure 5A–5C). Only in 1 position was a hotspot mutation observed (of the same category) in both a tumour suppressors and an oncogene (see Figure 5D–5F). This was MSA position 117 in the tyrosine protein kinase domain (Pkinase_Tyr).

Protein kinases (Pkinase and Pkinase_Tyr) can be thought of being in equilibrium between the open and closed conformations. Usually, other protein kinases phosphorylate the activating residues (S/T/Y) - moving the conformational equilibrium towards the open, active conformation, whereas protein phosphatases remove the phosphate groups shifting the conformational equilibrium back to the closed, inactive conformation. These processes leads to highly regulated control of the conformation and activation of kinase domains.

Dependant on their location within the kinase domain, missense mutations will often be better tolerated in one or other conformation of the protein kinase resulting in an alteration of the conformational equilibrium and constitutive activation (or in some cases deactivation) of the protein kinase. This is reflected in that the positions of the hotspots are generally different in the oncogenes and tumour suppressor flavours of this domain. This may not be the case in position 117 of the Pkinase_Tyr domain. Ten oncogene kinases have a mutation in this position, including the documented activating mutations FGFR2 N549S/K/H, the FGFR1 N546K and EGFR R776H mutations. However, the tumour suppressor MAP3K13 has an A218T mutation of unknown consequence at this position, which suggests that it may be possible to have a driver mutation that deactivates the protein at this position alternatively A218T may be an activating mutation.

### Genome wide hotspots

The final part of our analysis was to assess how many of the genome–wide hotspots we could putatively assign as activating/gain of function, or as loss of function. In total there were 954 missense hotspots in 423 domain families, 113 indels in 93 domain families and 506 truncations in 382 domain families of which ∼11% were co-located with an oncogene or tumour suppressor hotspot.

We were able to identify mutations in genes not previously related to cancer that aligned with well-established caner hotspots. These included 14 tyrosine protein kinase domains that had missense mutations co-located with activating BRAF V600E mutation including kinase suppressor of ras 2 (KSR2) p.R724W (117) [47], mixed lineage kinase domain like (MLKL) p.R264H (117) [48] lemour tyrosine kinase 3 (lmtk3) p.L195F (117) [49] and HCK P405S (343)[50]. Mutations at this position usually activate the kinase domain, suggesting that these proteins may be cancer gain of function drivers in rare cases. Similarly, 32 receptors from the 7tm_1 family that had mutations co-located with the A623V activating mutation in the thyrotropin receptor (TSHR) [45]. These included four chemokine receptors including three c-c chemokine receptors CCR3 (I238V), CCR6 (I253M), CCR8 (237T) and the CX3X chemokine receptor 1, CX3CR1, (I230N). Chemokines are small secreted proteins with an ability to prompt the migration of leucocytes. Both cell migration and metastasis show some similarities to leucocyte trafficking, which have lead to suggestions that chemokine receptors expressed on cancer cells may play a role in cancer development [51].

Of the remaining 89% of hotspots, 94% are located in ∼700 domain families not yet associated with well-documented oncogenes and tumours suppressors. This included a significant hotspot mutation in the AAA+ domain (PF00004), a large diverse protein family belonging to the AAA superfamily of P-loop NTP hydrolases, that utilise ATP to create conformational changes that are transduced into mechanical forces on macromolecule substrates. There is a mutation located at position 110 in the MSA of the domain. This includes mutations in WRN1P1 a DNA damage sensor (R306Q), the 26S protease regulatory subunit 6 (PSMC2) (R258H), and in paraplegin (SPG7) R391W. Structural analysis by SAAPdat [13] and mCSM [52] on SPG7, the only available PDB structure (2QZ4), suggests that the R391W mutation would destabilise the structure and disrupt protein-protein interactions.

## MATERIALS AND METHODS

### Mutation mapping

Protein sequences from COSMIC v71 [57] were mapped to UniProt [58] protein sequences using MD5 hashes and BLAST [59] using the MOKCa update protocol. Pfam domain boundaries were assigned to each protein and Fasta sequence files generated for each domain.

Somatic mutation data was extracted from the “Whole Genome Sequencing” (WGS) version of the COSMIC database V71 and processed using the MOKCa update protocol. 2,399,998 mutations from 15051 patient samples in 30 cancer types were mapped to the UniProt protein sequences. In total, 1,077,825 (45%) mutations could be mapped to conserved Pfam domains [60].

The mutations were classified into three subsets. Missense mutations, where usually a single base substitution changes the protein product by a single amino acid. Truncating mutations, which incorporate nonsense mutations and frameshift insertions and deletions. Truncations may just disrupt a single domain or result in complete destruction of the protein for example by nonsense mediated decay. Finally, inframe insertions and deletions (indels) were grouped together as they are relative infrequent, and both have the possibility of causing more severe disruptions to the protein product than a missense mutations. In total there were 727,525 missense, 69414 truncations and 2,958 indels mapped to 17,536 protein domains.

### Functional classification of TS and OG

The panther functional classification website was used to define the function of the proteins assigned as tumour suppressors and oncogenes. The DAVID website [61] was used to identify GO term [62] and KEGG [63] pathway enrichment for both datasets. For the 44 domains found in both tumour suppressors and oncogenes, the molecular function for each domain was assigned individually using domain information from Interpro website.

### Enriched domains

To find the domains enriched in mutations in tumour suppressors and oncogenes we compared the mutational frequency for each domain to the mutational frequency of a dataset of 450 “random” domains not related to cancer using a chi-square association test [53]. A Bonferroni correction was used to identify significantly mutated domains. Missense, truncations and indels were tested independently.

For the genome-wide study, the mutational burden in each single domain type was compared to that in all other domain types using a chi-square association test. Data was normalized by domain frequency, number of samples and domain length.

### Hotspot identification

A suite of Perl programs was used to generate and analyse hotspot domain positions. A multiple sequence alignment (MSA) was generated for all human domain fasta sequences, for each Pfam family using the MUSCLE (v3.8.31) alignment program [64]. Each mutation from each domain was mapped to a consensus position generated from the MSA and a consensus count was generated.

A binomial test was used to identify which positions had a significant number of mutations. If each individual mutation were to affect a random residue across the domain the frequency of mutations at each site would follow a binomial distribution. As such our null model states that there is an equal probability of a mutation occurring at each residue on the given domain.

Where n is the total number of mutations in the domain, k is the number of mutations falling at a specific residue and p the probability of any mutation affecting a specific residue we can find the probability of observing k mutations falling at any specific point in the domain by calculating the probability of a minimum of k mutations at that point and comparing it to our null model.

$$P\left(n\geq k\right)=\sum_{i=k}^{n}\left(\begin{array}{l} n\\ k \end{array}\right)p^{k}{\left(1-p\right)}^{n-k}$$

Missense, truncations and indels were tested independently and only positions where mutations occurred at least two were analysed. The results were amended by a Bonferroni correction. The overlap of hotspots between different mutational types were visualised with jvenn web application [54]

### MoKCA database

The MOKCa database (Mutations, Oncogenes and Knowledge in Cancer,http://strubiol.icr.ac.uk/extra/MOKCa) was developed to structurally and functionally annotate, and where possible predict, the phenotypic consequences of disease-associated mutations in proteins implicated in cancer. The initial database focused on protein kinases, but has now been extended include all the proteins from the human genome that are mutated in cancer.

### Populating the database with mutational data

Somatic mutation data from tumours from the COSMIC database (v71) have been mapped to their position in UniProt sequences. COSMIC use their own reference sequences (Ensembl transcripts), and although most COSMIC protein sequences (∼17000) match perfectly when mapped to UniProt sequences, for the remaining ∼4000 sequences the relationship is more complicated. Each COSMIC sequence was aligned with their corresponding UniProt sequence and when the sequences are not identical the alignment was stored in the database. This allows us to identify the position of the mutation with regard to the UniProt sequence, which provides the authoritative reference.

Each mutation is described its alteration to the protein structure, eg V600E. When this mutation has been reported on more one occasion each mutation is stored as the same aggregate and an aggregate count given. Different genetic changes that result in the same mutation are presented together at the protein level. Each disease type in which this mutation has been recorded is also presented on the protein overview page.

### Functional annotation of protein sequences and mutations

Functional annotations for each protein using a variety of databases have incorporated this into the new MOKCa database. These annotations include the identification and position of Pfam domain assignments within the protein sequence, and the positions of residues known or predicted to be affected by post-translational modifications including phosphorylation, glycosylation, and ubiquitination. Gene Ontology (GO) annotations and Prosite patterns [65] have also been obtained for each sequence.

### Structural mapping of mutations

The amino acid sequence for every Pfam-annotated domain for which COSMIC records a cancer-associated mutation has been scanned against the Protein Data Bank (PDB) [64] using PSI-BLAST, to map the mutation onto the protein structure of the affected human protein domains where the structure has been experimentally determined, or onto the most closely related homologous structure where the experimental structure is not known.

To identify which mutations mapped onto residues with structural density in the PDB file, PDB sequence to structure alignments from the SIFTS (Structure integration with function, taxonomy and sequence) initiative were utilized.

### Development of web-interface

The new web-interface for MOKCa database can accessed athttp://strubiol.icr.ac.uk/extra/mokca/ and can be searched by gene name or by UniProt accession. Users can also “browse the data from the gene data. To help identify those proteins we have identified subsets of proteins that are frequently mutated in cancer this includes, protein kinases [4], oncogenes and tumour suppressors [1], proteins involved in the DNA damage response (DDR) and those proteins that are current targets of chemotherapy and personalised cancer medicine regimes (drug targets) [55].

## CONCLUSIONS

In this study we have used recurrence to identify hotspot positions of somatic missense, indel and truncating mutations on over 5000 Pfam domain families. We analysed the data in tumour suppressors and oncogenes separately as we were particularly keen to find hotspots involved in activated proteins, and found that mutational hotspots in tumour suppressors and oncogenes usually occur in different types of domains, when they do occur in the same domain family, they occur at different positions in the domain. Our analysis also suggests that there may only be a small subset of domain types that can easily be activated by single small mutations.

Missense hotspots were frequently conserved in multiple members of Pfam domain families, however truncations were conserved far less frequently with many truncational hotspots occurring only in individual proteins. This may be because truncations often obliterate the functioning protein due to processing of the transcript by nonsense-mediated decay, so its position within a domain is far less crucial than for missense mutations. The large number of truncation hotspots observed in the whole genome dataset, suggest that there may be a large number of tumour suppressors not yet documented. Current statistical methods for analysing cohorts of cancer patients are designed to identify statistically significant mutations in single genes. Many of the tumour suppressors are part of large protein complexes where failure of any single component will result in loss of function of the complex as a whole. The mutational burden is thus distributed over all components of the complex, with no individual subunit being affected at a sufficient level to generate a statistically detectable signal.

Using the Cosmic v71 (WGS) we identified several indel mutations conserved in multiple member of domain families. As more genome sequencing studies are undertaken and the algorithms used to detect indels improve, it is likely that more indel hotspots will be identified.

We have also used our oncogene and tumour suppressor hotspots to identify co-located hotspots in 167 proteins as yet, not associated with cancer. This information enables us to assign putative gain or loss of function mutations in these proteins that may contribute to cancer progression. Using the biological knowledge associated with protein domains, such as structural information and evolutionary conservation, enables the transfer of knowledge from well studied oncogenes to less well studies homologues can lead to testable hypotheses of the effect of rare mutations in large cancer genomics datasets, and may lead to tractable therapeutic intervention points.

The domain hotspots identified within this study are available though the MOKCa database where mutations are annotated by driver types (http://strubiol.icr.ac.uk/extra/MOKCa).

## SUPPLEMENTARY MATERIALS FIGURES AND TABLES


















