# Supplementary material for: Identification and analysis of mutational hotspots in oncogenes and tumour suppressors
Source: Oncotarget. 2017 Feb 19;8(13):21290–304. doi: 10.18632/oncotarget.15514 (PMC5400584; doi:10.18632/oncotarget.15514)
Supplement: Supplementary file 1 [file oncotarget-08-21290-s001.pdf]

Identification and analysis of mutational hotspots in oncogenes and tumour suppressors

Supplementary Materials

Using the caret library in R we applied a 10-fold cross validation on a polynomial kernel support vector machine (SVM) to optimise and train a classifier to predict whether a known cancer driver gene is an oncogene or a tumour suppressor. We used the domains in the gene's protein product as the features. This feature space is not exclusive: 44 protein domains are observed in tumour suppressors and oncogenes. The number of oncogenes and tumour suppressors were balanced in the training set.

A gridsearch for optimised hyperparameters at cross validation found the optimised model (Degree = 2, scale = 1, C = 4) achieved a ROC AUC score of 0.72.

Using the optimised model we made predictions for a set of genes that have been reported to act as both tumour suppressor and oncogenes We found that 17 of the genes were predicted to be tumour suppressors, including TP53, DAXX and DDB2 with probabilities of greater than 0.94. Nine genes were classified as oncogenes including ERBB4, BCL10 and BTK with probabilities around 0.90, and 11 could not be resolved using this approach.

Supplementary Table 1: Domain based prediction of oncogenes and tumour suppressors

Gene	Tumour Suppressor	Oncogene	Molecular Genetics	Mutation Type
APOBEC3B	0.55929247	0.44070753	Dom	T
ARNT	0.095102486	0.904897514	Rec	D
ATP1A1	0.095098218	0.904901782	Dom	Mis, O
BCL10	0.095224618	0.904775382	Dom	T
BCL11B	0.095203935	0.904796065	Dom	T
BCORL1	0.806008175	0.193991825		Mis, N, F
BIRC3	0.095130035	0.904869965	Dom	D, F, N, T, Mis
BMPR1A	0.938376942	0.061623058	Rec	Mis, N, F
BTK	0.094104958	0.905895042	Dom	Mis
CARS	0.095202325	0.904797675	Dom	T
CBL	0.928809629	0.071190371	Dom/Rec	T, Mis, S, O
CBLC	0.938424388	0.061575612	Rec	M
CIC	0.938338612	0.061661388	Rec	Mis, F, S,T
CREBBP	0.89078437	0.10921563	Dom/Rec	T, N, F, Mis, O
CUX1	0.710284168	0.289715832	Dom	N,F,S,Mis,O,T
DAXX	0.938425004	0.061574996	Rec	Mis, F, N
DDB2	0.938416133	0.061583867	Rec	Mis, N
EPAS1	0.162013776	0.837986224	Dom	Mis
ERBB4	0.112293069	0.887706931	Dom	Mis, N
EZH2	0.796672637	0.203327363	Dom	Mis
FOXO1	0.559292713	0.440707287	Dom	T
FOXO3	0.559292049	0.440707951	Dom	T
FOXO4	0.559292065	0.440707935	Dom	T
GATA1	0.805997986	0.194002014	Dom	Mis, F
GATA3	0.938376083	0.061623917	Rec	F, N, S
IRF4	0.559292759	0.440707241	Dom	T
KLF4	0.65729295	0.34270705	Dom	Mis
LEF1	0.889684833	0.110315167		Mis, N
NOTCH1	0.936183145	0.063816855	Dom/Rec	T, Mis, O
NOTCH2	0.806010712	0.193989288	Dom/Rec	N, F, Mis
PTK6	0.346988219	0.653011781	Dom	Mis, N
QKI	0.729998966	0.270001034	Dom	Mis, F, T
RUNX1	0.55931142	0.44068858	Dom	T
TBX3	0.559318969	0.440681031	Dom	Mis, N, F, O
TET1	0.806006076	0.193993924	Dom	T
TP53	0.93836402	0.06163598	Rec	Mis, N, F, T
TP63	0.796034669	0.203965331		Mis, N, T

This table shows the results for each of the 37 genes labelled as both OG/TS in the Cancer Gene Census (v79 CGC). For each gene it describes the probability that the gene is a tumour suppressor, the probability the gene is an oncogene, the molecular genetics as described by CGC, and the type of mutation that is commonly found within the gene in cancer samples. Abbreviations: D, dominant; R, Recessive; M, Missense mutation; T, Translocation; D, large deletion; N, Nonsense mutation; F, Frameshift mutation; S, splice site mutation; O, other;

Supplementary Table 2: Domains enriched in missense mutations in tumour suppressors

Domains	No of domains	Enrichment score	*p*-value	Genes
P53	1	7108.33	0	TP53
WD40	3	1061.49	0	DDB2, FBXW7, TBL1XR1
DSPc	1	447.59	9.253E–197*	PTEN
VHL	1	324.28	7.4782E–143*	VHL
MH2	1	297.32	6.2691E–131*	SMAD4
PTEN_C2	1	235.49	2.0893E–103*	PTEN
P53_tetramer	1	228.79	2.0875E–100*	TP53
HLH	1	96.67	9.43726E–41*	MAX
RhoGAP	1	11.37	1.01375E–53*	PIK3R1
RB_B	1	6.69	1.60402E–29*	RB1
Sterol-sensing	1	3.65	2.68495E–16*	PTCH1
DED	1	2.72	2.59859E–07*	CASP8
MATH	1	2.61	0.00022429*	SPOP
Patched	1	2.23	0.007075266*	PTCH1
FERM_M	1	1.34	0.000495019*	NF2

The significant domains in tumour suppressors are listed by the Pfam domain name, the number of domains, the mutation enrichment expressed as the ratio of the observed number of domain mutations to the expected number of mutation, the Bonferroni corrected *p*-value and the gene names. The list sorted by enrichment score followed by the number of domains. *Indicates that the initial *p*-value was calculated using Fisher's exact test.

Supplementary Table 3: Domains enriched in truncation mutations in tumour suppressors

Domains	No of domains	Enrichment score	*p*-value	Genes
P53	1	639.27	1.2247E–40*	TP53
P53_tetramer	1	115.85	3.24858E–06*	TP53
PTEN_C2	1	7.56	7.581E–108*	PTEN
APC_crr	1	6.16	5.50276E–71	APC
DSPc	1	4.46	4.36254E–58	PTEN
RB_A	1	4.44	1.34185E–60	RB1
F-box-like	2	4.24	5.95979E–25	ECT2L, FBXW7
VHL	1	3.60	3.8446E–42	VHL
GATA	1	3.01	3.63272E–07	GATA3
WD40	2	2.56	2.021E–25	FBXW7, TBL1XR1
BAH	1	2.45	7.54557E–21	PBRM1
DUF3452	1	2.27	3.0659E–05	RB1
Bromodomain	2	1.98	9.05182E–20	PBRM1, SMARCA4
RhoGAP	1	1.95	0.0019881	PIK3R1
SH2	1	1.88	0.001241721	PIK3R1

The significant domains in tumour suppressors are listed by the Pfam domain name, the number of domains, the mutation enrichment expressed as the ratio of the observed number of domain mutations to the expected number of mutation, the Bonferroni corrected p-value and the gene names. The list sorted by enrichment score followed by the number of domains. *Indicates that the initial *p*-value was calculated using Fisher's exact test.

Supplementary Table 4: Domains enriched in indels mutations in tumour suppressors.

Domains	No of domains	Enrichment score	*p*-value	Genes
RhoGAP	1	16.32	6.87324E–68	PIK3R1
P53	1	9.65	6.4868E–103*	TP53

The significant domains in tumour suppressors are listed by the Pfam domain name, the number of domains, the mutation enrichment expressed as the ratio of the observed number of domain mutations to the expected number of mutation, the Bonferroni corrected *p*-value and the gene names. The list sorted by enrichment score followed by the number of domains. *Indicates that the initial *p*-value was calculated using Fisher's exact test.

Supplementary Table 5: Domains enriched in missense mutations in oncogenes. See Supplementary_Table_1

Supplementary Table 6: Domains enriched in truncation mutations in oncogenes. See Supplementary_Table_2

Supplementary Table 7: Domains enriched in indels mutations in oncogenes

Domains	No of domains	Enrichment score	*p*–value	Genes
zf–C2H2	2	34.93	2.90451E–68	MECOM, ZNF384
IL6Ra–bind	1	13.24	3.17974E–21	IL6ST
bZIP_2	1	10.91	7.43484E–17	CEBPA
PI3K_p85B	1	7.28	4.67624E–10	PIK3CA
Myb_DNA–bind_6	1	6.18	2.54389E–05	MYB

The significant domains in oncogenes are listed by the Pfam domain name, the number of domains, the mutation enrichment expressed as the ratio of the observed number of domain mutations to the expected number of mutation, the Bonferroni corrected *p*-value and the gene names. The list sorted by enrichment score followed by the number of domains.

Supplementary Table 8: Domains enriched in missense mutations in the whole genome. See Supplementary_Table_8

Supplementary Table 9: Domains enriched in truncation mutations in the whole genome. See Supplementary_Table_9

Supplementary Table 10: Domains enriched in indels mutations in the whole genome. See Supplementary_Table_10

Supplementary Table 11: The significantly enriched missense hotspots of mutations in the whole genome (WG), tumour suppressors (TS) and oncogenes (OG). See Supplementary_Table_11

Supplementary Table 12: The significantly enriched truncation hotspots of mutations in the whole genome (WG), tumour suppressors (TS) and oncogenes (OG). See Supplementary_Table_12

Supplementary Table 13: The significantly enriched indel hotspots of mutations in the whole genome (WG), tumour suppressors (TS) and oncogenes (OG). See Supplementary_Table_13

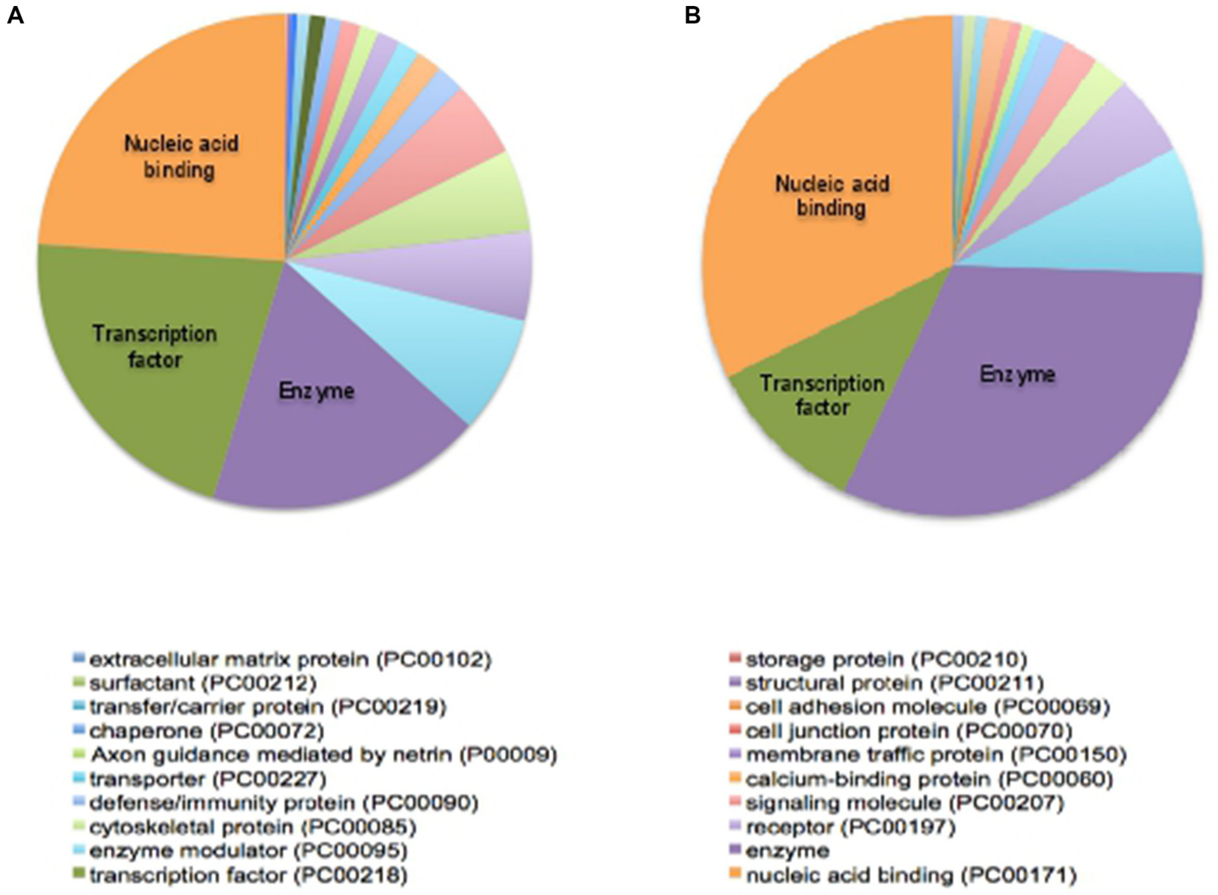

Supplementary Figure 1: Functional analysis of cancer proteins. The distribution of the protein functions (**A**) in 481 oncogenes, (**B**) in 131 tumour suppressors as determined by the DAVID functional annotation website.

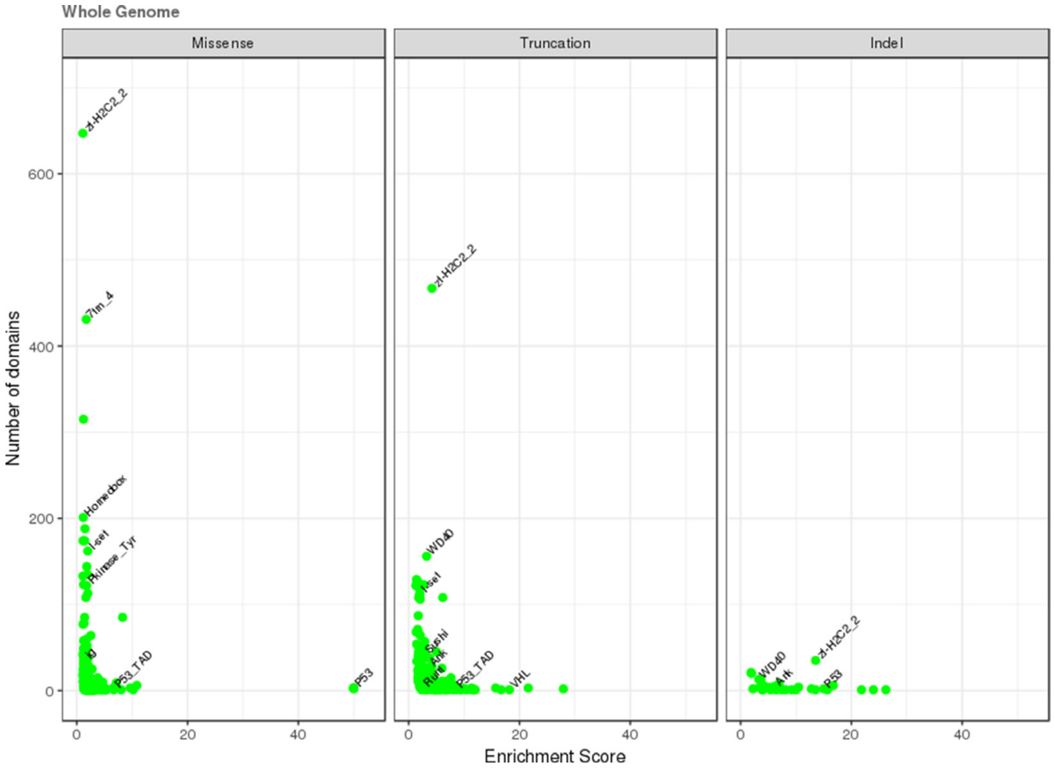

Supplementary Figure 2: Domains enriched in mutations within the whole genome. The number of domains in the dataset is plotted against the estimated mutational enrichment for that domain. Only domains with significant mutational enrichment (see methods) are shown. Missense, truncation and indel mutational enrichments were calculated for the whole genome. (**A**) Missense mutations, (**B**) truncation mutations, (**C**) indel mutations.

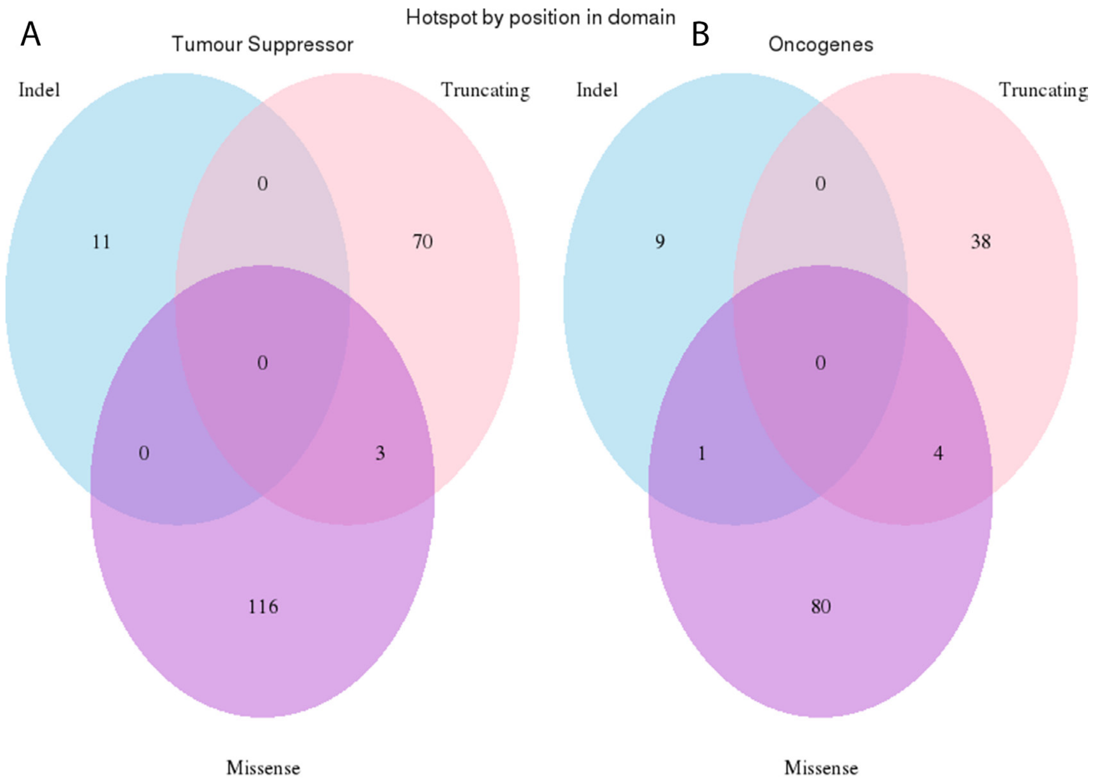

Supplementary Figure 3: Hotspot positions for missense, truncation and indel mutations. (A) hotspots in tumour suppressors, (**B**) hotspots in oncogenes. The figure illustrates the overlap in the position of the significant hotspots in missense (purple), truncation (pink) and indel (blue) mutations. Each circle represents the number of position in domains that contains a hotspot mutation, intersections illustrate when the same position in domain family is found with more than one class of mutation.
