# Supplementary material for: Identification and analysis of mutational hotspots in oncogenes and tumour suppressors
Source: Oncotarget. 2017 Feb 19;8(13):21290–304. doi: 10.18632/oncotarget.15514 (PMC5400584; doi:10.18632/oncotarget.15514)
Supplement: Supplementary file 3 [file oncotarget-08-21290-s003.docx]

**Supplementary Table 6:** Domains enriched in truncation mutations in oncogenes.

| **Domains** | **No of domains** | **Enrichment score** | ***p*-value** | **Genes** |
| --- | --- | --- | --- | --- |
| hEGF | 1 | 160.55 | 1.02E-10* | WIF1 |
| Activin_recp | 1 | 35.64 | 5.04E-46* | ACVR1 |
| Hairy_orange | 1 | 24.31 | 4.96096E-68 | HEY1 |
| Ig_3 | 1 | 23.02 | 6.23E-27* | KIT |
| NHR2 | 1 | 22.31 | 3.51145E-75 | RUNX1T1 |
| HMG_box | 1 | 21.73 | 2.58E-58 | TCF7L2 |
| Topo_C_assoc | 1 | 20.19 | 3.65738E-79 | TOP1 |
| zf-MYND | 1 | 19.78 | 9.82542E-31 | RUNX1T1 |
| FOP_dimer | 1 | 19.42 | 2.43729E-83 | FGFR1OP |
| AT_hook | 1 | 18.83 | 2.31732E-10 | HMGA1 |
| ITAM | 2 | 14.57 | 1.48941E-21 | CD79A, CD79B |
| BTG | 1 | 13.45 | 8.8208E-55 | BTG1 |
| MHCassoc_trimer | 1 | 12.89 | 1.25516E-30 | CD74 |
| zf-RING_5 | 1 | 12.88 | 1.4557E-18 | CCNB1IP1 |
| TSP_1 | 1 | 11.99 | 3.25985E-19 | RSPO3 |
| RBD | 2 | 11.71 | 1.12095E-50 | BRAF, RAF1 |
| IL2 | 1 | 11.30 | 1.71079E-46 | IL2 |
| Lep_receptor_Ig | 1 | 11.17 | 9.02978E-28 | CSF3R |
| MHC2-interact | 1 | 10.11 | 4.22037E-28 | CD74 |
| SSXT | 1 | 9.74 | 9.24142E-15 | SS18L1 |
| Fip1 | 1 | 9.65 | 2.04841E-09 | FIP1L1 |
| BTK | 1 | 9.21 | 1.53426E-05 | ITK |
| COX6C | 1 | 8.90 | 4.15347E-13 | COX6C |
| DUF1903 | 1 | 8.79 | 2.37765E-11 | MTCP1 |
| SH3_9 | 1 | 8.47 | 7.65367E-08 | LASP1 |
| Calreticulin | 1 | 8.15 | 2.71743E-48 | CALR |
| PH | 2 | 7.86 | 2.1782E-29 | AKT1, ITK |
| Pkinase | 6 | 7.30 | 1.56E-83* | ACVR1, AKT2, CDK6, IKBKB, MAP2K1, PIM1 |
| Pkinase_C | 1 | 7.20 | 5.98808E-05 | AKT2 |
| zf-C2H2_6 | 1 | 6.91 | 0.001798111 | ZNF521 |
| eIF3_N | 1 | 6.83 | 1.99578E-13 | EIF3E |
| Tropomyosin | 1 | 6.63 | 2.75126E-22 | TPM3 |
| SWIB | 1 | 6.54 | 2.42072E-06 | MDM4 |
| V-set | 4 | 5.94 | 1.27502E-36 | CD274, CD79A, CD79B, KDR |
| HIT | 1 | 5.89 | 3.00854E-06 | FHIT |
| SRC-1 | 1 | 5.84 | 0.000236723 | NCOA2 |
| zf-H2C2_2 | 6 | 5.78 | 3.8107E-19 | BCL11A, BCL6, PLAG1, ZBTB16, ZNF278, ZNF331 |
| Cation_ATPase_N | 2 | 5.73 | 1.36642E-08 | ATP1A1, ATP2B3 |
| COLFI | 1 | 5.49 | 6.70854E-13 | COL1A1 |
| Death | 1 | 5.46 | 0.000767025 | MYD88 |
| RRM_5 | 2 | 5.17 | 5.39726E-05 | RBM15, U2AF1 |
| DIL | 1 | 5.13 | 4.16087E-10 | MLLT4 |
| IMD | 1 | 5.10 | 5.01306E-10 | ARHGAP26 |
| NAP | 1 | 5.03 | 4.39945E-09 | SET |
| TIR | 1 | 4.91 | 3.14411E-05 | MYD88 |
| RunxI | 1 | 4.90 | 5.39697E-08 | RUNX1 |
| PAS_11 | 2 | 4.45 | 2.35956E-07 | ARNT, NCOA2 |
| PD-C2-AF1 | 1 | 4.41 | 2.2028E-08 | POU2AF1 |
| STAT6_C | 1 | 3.99 | 0.000183502 | STAT6 |
| zf-B_box | 2 | 3.87 | 0.003255112 | TRIM24, TRIM27 |
| Nucleoplasmin | 1 | 3.82 | 0.001320811 | NPM1 |
| WD40 | 2 | 3.71 | 0.018462408 | STRN, TRAF7 |
| zf-C2H2 | 6 | 3.71 | 0.00665852 | BCL11B, MECOM, PLAG1, PRDM16, ZBTB16, ZNF384 |
| Ran_BP1 | 1 | 3.50 | 1.65198E-08 | RANBP2 |
| Ribophorin_I | 1 | 3.25 | 6.4007E-06 | RPN1 |
| Gly_rich | 1 | 3.08 | 0.022956872 | ALK |
| DUF3827 | 1 | 2.32 | 0.014229016 | KIAA1549 |

The significant domains in oncogenes are listed by the Pfam domain name, the number of domains, the mutation enrichment expressed as the ratio of the observed number of domain mutations to the expected number of mutation, the Bonferroni corrected p-value and the gene names. The list sorted by enrichment score followed by the number of domains.

*Indicates that the initial *p*-value was calculated using Fisher’s exact test.
