# Supplementary material for: Identification and analysis of mutational hotspots in oncogenes and tumour suppressors
Source: Oncotarget. 2017 Feb 19;8(13):21290–304. doi: 10.18632/oncotarget.15514 (PMC5400584; doi:10.18632/oncotarget.15514)
Supplement: Supplementary file 4 [file oncotarget-08-21290-s004.docx]

**Supplementary Table 8:** Domains enriched in missense mutations in the whole genome.

| **Domains** | **No of domains** | **Enrichment score** | ***p*-value** | **Genes** |
| --- | --- | --- | --- | --- |
| P53 | 3 | 67065.45 | 0* | TP53, TP63, TP73 |
| FRG1 | 3 | 9112.99 | 2.8172E-108* | C20orf80, FRG1B, FRG1 |
| VHL | 2 | 1561.49 | 1.33957E-14* | VHL, VHLL |
| P53_tetramer | 3 | 1137.20 | 1.8266E-09* | TP53, TP63, TP73 |
| 4F5 | 6 | 10.85 | 2.9219E-24* | ZNF706, LOC51123, ENSG00000176050, SERF2, SERF1B, SERF1A, |
| OGFr_III | 1 | 10.20 | 9.78319E-16 | OGFR |
| NHR2 | 3 | 9.66 | 9.71563E-28* | RUNX1T1, CBFA2T2, CBFA2T3 |
| Histone | 85 | 8.27 | 0* | HIST1H3A, HIST1H3B, HIST1H3C, HIST1H3D, HIST1H3E, HIST1H3F, HIST1H3G, HIST1H3H, HIST1H3I, HIST1H3J, HIST1H4I, HIST1H4A, HIST1H4B, HIST1H4D, HIST1H4E, HIST1H4K, HIST1H4L, HIST2H4A, HIST2H4B, HIST1H2BC, HIST1H2BE, HIST1H2BI, HIST1H4C, HIST1H4F, HIST1H4H, HIST1H4J, HIST4H4, HIST1H2AG, HIST1H2AI, HIST1H2AM, HIST1H2BF, HIST1H2BG, HIST1H2BD, HIST1H2BO, HIST1H2AA, HIST2H2BE, H3F3C, HIST1H2AC, H2BFWT, HIST1H2AE, HIST1H2BH, HIST1H2AK, HIST1H2AL, H2AFY, HIST1H2BL, HIST2H2AC, HIST1H2BM, HIST1H2BA, HIST2H2AB, HIST1H4G, HIST2H2BF, HIST3H3, HIST1H2BB, HIST1H2BK, HIST1H2AJ, HIST3H2BB, HIST1H2AD, HIST1H2BJ, H2AFV, H2AFY2, HIST1H2AH, SOS2, H3F3A, H3F3B, HIST2H3C, HIST2H3A, HIST2H3D, CENPA, H2AFZ, H2AFZP2, HIST3H2A, SOS1, HIST1H2BN, H2AFX, HIST1H2AB, H2AFJ, HIST2H3PS2, LOC340096, ENSG00000196285, Q6TXQ4, H2AFB1, HIST2H2AA4, H2AFB2, H2AFB3, ENSG00000158403 |
| Titin_Z | 1 | 8.01 | 9.37089E-97* | TTN |
| PI3Ka | 9 | 7.14 | 0* | PIK3CA, PIK3C2G, PIK3CG, PIK3C3, PIK3CB, PI4KA, PIK3C2A, PIK3C2B, PIK3CD |
| DUF676 | 3 | 6.76 | 2.3579E-180* | FAM135B, LOC51059, FAM135A |
| P53_TAD | 1 | 6.68 | 2.10105E-29 | TP53 |
| DUF3657 | 3 | 6.61 | 3.7555E-114 | FAM135B, LOC51059, FAM135A, KIAA0556 |
| LELP1 | 1 | 5.30 | 6.08712E-38 | LELP1 |
| Neuromodulin | 1 | 5.28 | 3.59853E-69 | GAP43 |
| TAT_ubiq | 1 | 5.27 | 3.99659E-14 | TAT |
| PDEase_I_N | 3 | 5.10 | 6.37718E-75* | PDE1A, PDE1C, PDE1B |
| RHS_repeat | 1 | 5.09 | 1.87652E-18 | ODZ4, |
| Fer4_20 | 1 | 4.71 | 3.56867E-35 | DPYD |
| IRK_N | 2 | 4.70 | 1.5859E-27 | KCNJ12, KCNJ2 |
| Drf_FH1 | 2 | 4.67 | 3.3343E-143 | FMN2, DIAPH1 |
| PTEN_C2 | 10 | 4.65 | 0* | PTEN, TPTE, TPTE2, ENSG00000197585, Q5JV89, DNAJC6, TENC1, TNS1, TNS3, GAK |
| HNH | 1 | 4.61 | 3.85057E-11 | ZRANB3 |
| Pro-rich | 6 | 4.53 | 0* | PRB2, PRB3, PRB4, PRB1, PRH2, PRR4 |
| FAM47 | 8 | 4.51 | 0* | FAM47C, LOC442444, FAM47B, ENSG00000185448, FAM47A, Q6ZV65, FAM47E, LOC139249 |
| POT1 | 1 | 4.51 | 5.09747E-37 | POT1 |
| PHTB1_C | 1 | 4.33 | 2.84216E-68* | BBS9 |
| PapD-like | 4 | 4.25 | 2.3517E-218* | HYDIN, LOC652153, LOC652737, SPAG17 |
| Furin-like | 7 | 4.17 | 6.9648E-190* | EGFR, ERBB3, ERBB4, ERBB2, INSRR, IGF1R, INSR |
| Ig_Tie2_1 | 1 | 3.92 | 6.84564E-19 | TEK |
| WT1 | 1 | 3.89 | 4.25703E-67 | WT1 |
| FISNA | 2 | 3.80 | 1.85713E-27 | NLRP12, NLRP3 |
| Ephrin_lbd | 15 | 3.76 | 0* | EPHA6, ENSG00000188974, EPHA3, EPHB1, EPHA8, EPHA10, EPHA1, EPHB2, EPHA7, EPHA5, EPHA4, EPHB6, EPHB3, EPHA2, EPHB4 |
| DUF4546 | 2 | 3.68 | 2.40839E-59* | C1orf49, TEX35 |
| VGCC_beta4Aa_N | 4 | 3.60 | 7.88915E-28 | CACNB2, CACNB4, CACNB1, CACNB3 |
| Fra10Ac1 | 1 | 3.57 | 1.18578E-18 | FRA10AC1 |
| COX6C | 1 | 3.47 | 8.43258E-10 | COX6C |
| HS1_rep | 2 | 3.38 | 8.10858E-56 | CTTN, HCLS1 |
| DUF3451 | 6 | 3.34 | 1.6598E-164* | SCN1A, SCN2A, SCN3A, SCN9A, SCN5A, SCN8A |
| Glyco_tran_28_C | 2 | 3.26 | 8.89094E-34 | GLT28D1, ALG13 |
| Cylicin_N | 2 | 3.25 | 2.82679E-28 | CYLC1, CYLC2 |
| Iso_dh | 5 | 3.24 | 1.2612E-173* | IDH1, IDH2, IDH3B, IDH3G, IDH3A |
| Sgf11 | 1 | 3.22 | 0.041537639 | ATXN7L3 |
| PPAK | 1 | 3.21 | 3.89343E-63* | TTN |
| Peptidase_M43 | 2 | 3.19 | 3.29376E-40 | PAPPA2, PAPPA |
| TAFH | 5 | 3.19 | 5.24869E-62 | RUNX1T1, CBFA2T2, TAF4, CBFA2T3, TAF4B |
| zf-RING_4 | 1 | 3.17 | 0.000560817 | CNOT4 |
| Lge1 | 1 | 3.11 | 1.01116E-11 | PPHLN1 |
| DUF500 | 1 | 3.11 | 1.49114E-13 | SH3YL1, |
| TAFA | 5 | 3.08 | 1.83053E-52 | FAM19A1, FAM19A2, FAM19A5, FAM19A4, FAM19A3 |
| Microtub_assoc | 2 | 3.04 | 2.15517E-15 | PDE4DIP, CDK5RAP2 |
| TRP_2 | 7 | 3.02 | 3.52511E-48 | TRPC7, ENSG00000254809, TRPC3, TRPC5, TRPC4, TRPC6, TRPC1 |
| Glutenin_hmw | 1 | 2.98 | 5.47435E-37 | TXNDC2 |
| DUF3583 | 1 | 2.98 | 8.19093E-35 | PML |
| BRE | 1 | 2.92 | 3.79883E-33 | BRE |
| Epiglycanin_TR | 1 | 2.91 | 2.60189E-75 | MUC21 |
| Nuf2 | 1 | 2.86 | 1.08317E-12 | NUF2 |
| IGF2_C | 1 | 2.82 | 0.005760959 | IGF2 |
| Cadherin_C | 25 | 2.77 | 2.7745E-241* | CDH18, CDH10, CDH7, CDH9, CDH8, DSC1, CDH12, CDH6, CDH11, CDH24, DSC2, DSG4, DSC3, CDH4, CDH20, CDH2, CDH19, DSG1, CDH1, CDH5, DSG3, CDH15, DSG2, CDH3, CDH22 |
| EZH2_WD-Binding | 2 | 2.77 | 0.004895055 | EZH2, EZH1 |
| DUF1087 | 3 | 2.73 | 1.08988E-14 | CHD4, CHD5, CHD3 |
| DUF2439 | 1 | 2.72 | 0.000273024 | C4orf21, |
| UNC-79 | 2 | 2.70 | 1.30388E-66* | KIAA1409, UNC79 |
| zf-C4H2 | 1 | 2.69 | 1.1918E-15 | ZC4H2 |
| Filaggrin | 1 | 2.67 | 3.29168E-73* | FLG, |
| DUF3591 | 2 | 2.66 | 1.74498E-73 | TAF1, TAF1L |
| KRAP_IP3R_bind | 4 | 2.65 | 3.14813E-48 | KIAA0748, LOC223075, CCDC129, SSFA2 |
| KAT11 | 2 | 2.64 | 9.5709E-47 | CREBBP, EP300 |
| Lipocalin_7 | 2 | 2.64 | 4.80138E-18 | FABP6, FABP1 |
| LIM_bind | 2 | 2.62 | 9.16617E-30 | LDB2, LDB1 |
| Casein_kappa | 1 | 2.61 | 2.75733E-10 | CSN3 |
| K_channel_TID | 1 | 2.60 | 0.002104297 | KCNA4 |
| WTX | 4 | 2.59 | 1.0931E-99 | AMER1, FAM123B, FAM123A, FAM123C |
| Asp_protease | 4 | 2.56 | 5.15604E-33 | DDI1, NRIP3, DDI2, NRIP2 |
| Cadherin_2 | 64 | 2.55 | 3.6706E-303 | PCDH11X, PCDHA8, PCDHGA1, PCDHA2, PCDHGA3, PCDHA7, PCDHA6, PCDH11Y, PCDHA1, PCDHA4, PCDH18, PCDHA5, PCDHA10, PCDHA9, PCDHGA2, PCDHG, PCDHGB3, PCDHA3, PCDH10, PCDHGB2, PCDHGA5, PCDHA11, PCDHB7, PCDHB12, PCDHB5, PCDHGA12, PCDHGA6, PCDHGA10, PCDHB4, PCDHGA4, PCDHGC3, PCDHB2, PCDHB3, PCDHGC5, PCDHA12, PCDHGA7, PCDHGA9, PCDHA13, PCDHAC1, PCDH9, PCDH17, PCDHAC2, PCDHB11, PCDHGB1, PCDHGB6, PCDHB1, PCDH8, PCDHB14, PCDHGA8, PCDHB6, PCDHB15, PCDHB16, PCDHGC4, PCDHGB7, PCDHB10, PCDH19, PCDH1, PCDH7, PCDH12, PCDHGA11, PCDHGB4, PCDHB18, PCDHB8, PCDHB13 |
| Lig_chan-Glu_bd | 9 | 2.52 | 7.81655E-56* | GRIK2, GRIA2, GRIA3, GRIK3, GRIK1, GRIA1, GRIA4, GRIK4, GRIK5 |
| Recep_L_domain | 7 | 2.49 | 2.32054E-81 | ERBB3, EGFR, ERBB4, INSRR, INSR, IGF1R, ERBB2, |
| Trefoil | 6 | 2.48 | 3.31782E-32 | MGAM, ZP4, SI, TFF2, TFF1, GAA |
| ADSL_C | 1 | 2.46 | 0.003681802 | ADSL |
| DUF4592 | 4 | 2.45 | 9.16671E-29 | KIAA1210, ENSG00000250423, KIAA1211, C2orf55 |
| Crisp | 3 | 2.44 | 5.43642E-08 | CRISP3, CRISP2, CRISP1 |
| DUF3508 | 1 | 2.42 | 5.97497E-15 | C6orf165 |
| RB_B | 3 | 2.39 | 2.57382E-25 | RB1, RBL2, RBL1 |
| AKAP_110 | 3 | 2.39 | 1.22057E-82 | AKAP3, AKAP4, SPHKAP |
| DUF4590 | 1 | 2.37 | 0.000223292 | C1orf173 |
| Dermcidin | 1 | 2.36 | 0.007242241 | DCD |
| DUF4638 | 1 | 2.36 | 8.57724E-13 | C16orf78, |
| BRCA-2_OB1 | 1 | 2.35 | 4.28206E-05 | BRCA2 |
| OGG_N | 1 | 2.34 | 0.000351802 | OGG1 |
| PDGF_N | 2 | 2.33 | 6.0752E-06 | PDGFB, PDGFA |
| Cu2_monooxygen | 3 | 2.33 | 4.36187E-19 | MOXD1, PAM, DBH |
| AAA_14 | 1 | 2.32 | 0.000191304 | NAV3 |
| T4_deiodinase | 3 | 2.30 | 4.89717E-37 | DIO2, DIO3, DIO1 |
| Neuregulin | 2 | 2.28 | 4.97806E-40 | NRG1, NRG2 |
| Not3 | 1 | 2.28 | 1.31376E-09 | CNOT3 |
| DUF1227 | 1 | 2.26 | 6.18411E-05 | ERCC2 |
| PI3K_C2 | 8 | 2.25 | 6.86461E-58 | PIK3CA, PIK3CG, PIK3C2G, PIK3CD, PIK3C3, PIK3CB, PIK3C2A, PIK3C2B |
| SRRM_C | 3 | 2.23 | 2.45186E-07 | KIAA1853, SRRM4, ENSG00000197630 |
| LCE | 17 | 2.22 | 3.03825E-67 | LCE2B, LCE1F, LCE3D, LCE2D, LCE2A, LCE5A, LCE2C, LCE3E, LCE1A, LCE1B, LCE3A, LCE1C, LCE4A, LCE1D, LCE1E, LCE3B, LCE3C |
| HIN | 4 | 2.21 | 4.86526E-36 | PYHIN1, MNDA, IFI16, AIM2 |
| SLAM | 1 | 2.20 | 0.002062904 | SLAMF1 |
| FTCD | 1 | 2.20 | 0.000280127 | FTCD |
| DUF2371 | 4 | 2.20 | 1.40277E-25 | TMEM200A, KIAA1913, TMEM200C, TMEM200B |
| NGF | 3 | 2.19 | 2.97643E-13 | BDNF, NTF3, NGF |
| Noelin-1 | 3 | 2.18 | 3.32952E-15 | OLFM3, OLFM2, OLFM1 |
| TBP-binding | 2 | 2.18 | 0.002184375 | TAF1, TAF1L |
| Cornifin | 4 | 2.17 | 1.30731E-14 | SPRR3, SPRR1A, SPRR1B, SPRR4 |
| Dickkopf_N | 4 | 2.17 | 8.11346E-07 | DKK2, DKK1, DKK4, DKK3 |
| Xin | 2 | 2.17 | 6.38024E-12 | XIRP2, XIRP1 |
| MH2 | 8 | 2.16 | 3.76567E-68 | SMAD4, SMAD3, SMAD9, SMAD2, SMAD6, SMAD1, SMAD7, SMAD5 |
| ROK | 1 | 2.15 | 1.60969E-05 | GNE |
| DNA_primase_lrg | 1 | 2.15 | 6.25996E-09 | PRIM2 |
| Na_trans_assoc | 10 | 2.15 | 3.23932E-83* | SCN5A, SCN1A, SCN3A, SCN2A, SCN11A, SCN10A, SCN7A, SCN9A, SCN8A, SCN4A |
| Runt | 3 | 2.14 | 2.02292E-14 | RUNX1, RUNX2, RUNX3 |
| CitMHS | 1 | 2.14 | 5.62497E-15 | OCA2 |
| Myc_target_1 | 1 | 2.14 | 2.30373E-05 | MYCT1 |
| DUF4483 | 1 | 2.12 | 3.27599E-05 | C9orf171 |
| CENP-N | 1 | 2.11 | 5.27789E-11 | CENPN |
| MYCBPAP | 1 | 2.11 | 2.24376E-14 | MYCBPAP |
| DUF4552 | 1 | 2.11 | 3.14894E-14 | C12orf40 |
| PaaSYMP | 2 | 2.10 | 1.37785E-09 | C3orf15, MAATS1 |
| FragX_IP | 2 | 2.10 | 2.96435E-62 | CYFIP2, CYFIP1 |
| Fer2_2 | 2 | 2.09 | 0.002428944 |  |
| TCL1_MTCP1 | 3 | 2.07 | 2.78978E-10 | TCL1B, MTCP1, TCL1A |
| KCNQC3-Ank-G_bd | 2 | 2.07 | 9.86672E-09 | KCNQ2, KCNQ3 |
| RB_A | 3 | 2.06 | 7.91895E-19 | RB1, RBL1, RBL2 |
| Nebulin | 4 | 2.06 | 2.2534E-119* | NEB, NEBL, NRAP, LASP1 |
| CAGE1 | 1 | 2.06 | 2.47695E-15 | CAGE1 |
| SIT | 4 | 2.06 | 4.13307E-12 | TRAT1, CD300LF, CD300A, SIT1 |
| LRR_5 | 6 | 2.05 | 1.3409E-28 | LRFN5, LRRC4C, LHCGR, LRRC4B, LRRN1, LRRC8A |
| Fe-ADH | 1 | 2.05 | 5.66669E-12 | ADHFE1 |
| PROL5-SMR | 3 | 2.05 | 1.86883E-08 | SMR3A, SMR3B, PROL1 |
| Band_3_cyto | 9 | 2.05 | 2.29718E-83 | SLC4A10, SLC4A8, SLC4A4, SLC4A5, SLC4A7, SLC4A9, SLC4A3, SLC4A1, SLC4A2 |
| Ant_C | 2 | 2.03 | 0.000460232 | ANTXR1, ANTXR2 |
| Protocadherin | 5 | 2.03 | 6.08116E-50 | PCDH11X, PCDH9, PCDH7, PCDH11Y, PCDH1 |
| GAGE | 30 | 2.01 | 2.8557E-86 | GAGE2A, GAGE12F, GAGE12G, GAGE12I, GAGE12C, GAGE12E, GAGE12D, GAGE2B, GAGE2C, GAGE13, GAGE12J, GAGE2D, PIWIL1, GAGE12H, PAGE2, PAGE1, XAGE5, GAGE10, GAGE2E, PAGE5, GAGE1, ENSG00000255738, PAGE2B, PAGE3, XAGE3, PAGE4, XAGE2B, XAGE1A, XAGE1B, XAGE1E |
| ANF_receptor | 37 | 2.01 | 0* | GRM8, GRM7, GRM5, GRIK2, GRM4, GRIA2, GRIA3, GRIA4, GRIK1, GRIK3, GRM3, GRIK5, GRID2, GPRC6A, CASR, GRM1, GRID1, GRIA1, TAS1R2, GABBR2, GRM6, NPR2, GRIN2B, NPR3, GRIK4, GRM2, TAS1R1, GRIN2A, GUCY2C, GUCY2F, GRIN1, GABBR1, GUCY2D, NPR1, TAS1R3, GRIN2C, GRIN2D |
| NMDAR2_C | 3 | 2.00 | 1.40646E-41 | GRIN2A, GRIN2B, GRIN2C |
| Dzip-like_N | 2 | 2.00 | 1.62854E-05 | DZIP1L, DZIP1 |
| Cadherin | 113 | 2.00 | 0* | CDH18, PCDHA10, PCDH11X, PCDHA5, PCDHA4, PCDHA8, PCDHA6, PCDHA7, FAT4, PCDHA1, PCDHA2, PCDHGA1, PCDHGA2, PCDHG, CDH7, PCDHA9, FAT3, CDH10, DSC3, DSG4, CDH11, PCDHA11, PCDHB8, CDH9, DCHS2, PCDHB10, PCDH20, PCDH11Y, CDH8, PCDHB2, PCDHGA12, PCDHB11, PCDHGB3, CDH1, PCDHA13, DSC1, PCDHB3, PCDH10, PCDHB12, DSC2, PCDHA3, PCDHB7, CDHR1, PCDHGA4, PCDHB4, CDH12, PCDHB16, FAT, FAT1, PCDHGB2, PCDHGB1, PCDH15, CDH4, PCDHGA6, PCDHGA10, PCDHB5, PCDHB14, CDH26, PCDHA12, PCDHGA3, PCDHB6, PCDH17, CDH6, PCDHAC1, PCDHB15, PCDHGA5, PCDHB13, DSG3, PCDHB1, CDH5, PCDHGA7, PCDHGC3, PCDHGC5, CDH2, DSG1, PCDH18, CDHR3, PCDHGA8, PCDH19, CDH24, CDHR2, PCDHGB6, CDH22, CDH13, RET, PCDHGA11, CDH19, PCDHGB7, PCDHGB4, PCDH1, PCDH9, CDH16, DSG2, PCDHGA9, CLSTN2, PCDHAC2, CDH20, PCDHB18, FAT2, CDH17, CDH3, CDH23, PCDH7, CELSR3, CELSR1, PCDH12, CELSR2, DCHS1, PCDHGC4, CDH15, CDHR4, PCDH8, CLSTN1 |
| Glyco_hydro_79n | 2 | 2.00 | 1.85025E-12 | HPSE2, HPSE |
| PH_11 | 4 | 1.99 | 1.69703E-10 | C10orf81, PLEKHS1, ADAP1, ADAP2 |
| I-set | 162 | 1.99 | 0* | TTN, FGFR2, NTM, PTPRD, NTRK3, NFASC, ROBO1, OPCML, CADM2, MDGA2, MXRA5, IL1RAPL1, PXDNL, NTRK2, FGFR1, FLT1, Q7Z2S2, CNTN5, CNTN4, ROBO2, LRFN5, LINGO2, DCC, NRG1, IGSF22, CNTN1, IL1RL1, CNTN6, JAM2, FSTL5, IGSF9B, MYBPC1, DSCAM, MAG, SIGLEC10, OBSCN, SIGLEC12, LRIT3, PDGFRA, LRIG1, MYPN, SIGLEC6, NCAM2, MDGA1, ROBO3, LRRC4B, HMCN1, MYOM3, SDK2, HEPACAM2, UNC5C, LRRC4C, MYOM2, IGSF5, PXDN, FGFR3, DSCAML1, FLT4, KDR, MYLK, CHL1, UNC5D, WFIKKN2, SDK1, L1CAM, IGSF9, EMB, PAPLN, CNTN3, MYBPC3, NRCAM, IGFN1, BSG, NEGR1, ALPK2, LSAMP, LRRN3, LRRN1, PALLD, PRTG, KIRREL, ALPK3, KIRREL2, LRFN1, KIRREL3, VCAM1, NRG2, ROR1, TYRO3, MYBPH, LRRN2, LRIG3, SPEG, BOC, IGSF10, FGFRL1, IGFBP7, LINGO4, MYBPC2, OBSL1, ADAMTSL1, ROBO4, NPTN, ISLR, SIGLEC1, NPHS1, MFAP3L, ROR2, LRIG2, CD101, CADM1, HSPG2, TMIGD1, UNC5A, MYOM1, FGFR4, MUSK, PDGFRB, TRIO, UNC5B, CNTN2, LRIT2, LRFN2, IGFBPL1, MYOT, GPR116, LRRC4, CEACAM5, GPR125, ADAMTSL3, PTK7, LRIT1, IGDCC3, NEO1, MERTK, VSIG2, IGDCC4, NEXN, LINGO1, SEMA3C, CDON, PTPRF, PTPRS, IL1R1, MYBPHL, FSTL4, LRRC24, LRFN4, WFIKKN1, LRFN3, MFAP3, SIGLEC11, KAZALD1, CCDC141, VSIG10, LINGO3, SIGLEC16, ENSG00000215428, TRAD, KALRN, PDGFRL, Q8TCI8 |
| ATP_Ca_trans_C | 4 | 1.99 | 4.28017E-08 | ATP2B2, ATP2B4, ATP2B3, ATP2B1 |
| GF_recep_IV | 6 | 1.99 | 2.90752E-28 | EGFR, ERBB4, ERBB3, PCSK5, PCSK6, ERBB2 |
| DZR | 2 | 1.98 | 7.91732E-08 | C20orf12, DZANK1 |
| TMEM71 | 1 | 1.98 | 0.02234329 | TMEM71 |
| DEP | 21 | 1.98 | 1.19429E-50 | RGS7, PREX2, RGS6, PREX1, DEPDC7, DEPDC5, DEPDC1B, RAPGEF4, DVL2, PLEK, DEPDC4, RGS9, PIKFYVE, DEPTOR, DEPDC1, GPR155, PLEK2, DVL3, RAPGEF3, DVL1, RGS11 |
| SK_channel | 5 | 1.97 | 2.50691E-19 | KCNN2, LOC145814, KCNN1, KCNN3, KCNN4 |
| MCC-bdg_PDZ | 2 | 1.96 | 1.96831E-05 | MCC, USHBP1 |
| CRAL_TRIO_N | 12 | 1.96 | 2.22962E-19 | CLVS2, C6orf213, CLVS1, RLBP1, SEC14L1, TTPAL, SEC14L3, SEC14L4, SEC14L5, SEC14L6, TTPA, PTPN9 |
| COX7B | 2 | 1.95 | 0.028560987 | COX7B, COX7B2 |
| FYVE_2 | 11 | 1.95 | 5.57404E-31 | RIMS2, RIMS1, RPH3A, SYTL4, MYRIP, SYTL5, SYTL3, RPH3AL, MOBP, MLPH, SYTL2 |
| Anth_Ig | 2 | 1.94 | 0.000979845 | ANTXR1, ANTXR2 |
| SelR | 3 | 1.94 | 8.51547E-08 | MSRB3, MSRB2, SEPX1 |
| PHTB1_N | 1 | 1.94 | 8.20719E-10 | BBS9 |
| Vinculin | 6 | 1.94 | 3.1827E-110* | CTNNA2, CTNNA3, CTNNA1, VCL, CTNNAL1, Q16370 |
| zf-CXXC | 11 | 1.92 | 6.10825E-14 | MBD1, KDM2B, MLL, MLL4, CXXC5, CXXC4, TET1, KDM2A, CXXC1, FBXL19, DNMT1 |
| Alpha_kinase | 6 | 1.92 | 1.3649E-29 | TRPM6, ALPK2, EEF2K, ALPK3, ALPK1, TRPM7 |
| Kv2channel | 2 | 1.92 | 1.63922E-12 | KCNB2, KCNB1 |
| Phospholip_A2_2 | 2 | 1.92 | 5.63526E-05 | PLA2G3, PROCA1 |
| PGC7_Stella | 6 | 1.91 | 1.50127E-23 | DPPA3, LOC401611, ENSG00000185095, ENSG00000188831, FAM156A, FAM156B |
| Ras | 135 | 1.91 | 0* | KRAS, NRAS, HRAS, RAC1, RAB37, RERGL, GRLF1, ARHGAP35, RHOA, RIT2, RAB3C, RAB28, RAB19, RAB19B, RAB39B, RERG, RASL12, RIT1, RHOH, RALB, RHOBTB2, RASD2, RAB34, RHEB, RHOJ, DIRAS1, REM1, RAP2C, RAB9B, RHOB, DIRAS2, RASEF, RAB33A, RAB3B, RALA, DNAJC27, RAB27B, GEM, RND1, RAP1A, NKIRAS2, RAB6C, DIRAS3, RAB6A, RAB20, RAB5A, RAB15, RAB5C, RABL3, RAB40C, RAC2, RAP1B, RAB6B, RND3, CDC42, MRAS, RHOQ, RASL10B, RAB3D, RAB24, RASL11A, RAB39, RAB36, RAB10, RAB30, RAB2A, RHOC, RAB40AL, RAB7L1, RAB21, RAP2B, RRAS2, RAB8A, RAB11B, RAB27A, RAB1B, RRAD, RAB5B, RAB35, RHOU, RAB41, RAB4A, RHOF, ERAS, RAB14, RAB2B, REM2, RAB40B, RAB38, RAB12, RAB18, RABL2A, RAB13, RAB23, NKIRAS1, RAB8B, RHOV, RAB7A, RHOG, RAP2A, RAB31, RHOBTB1, RAB40A, RHOT1, RAB22A, RAN, RAB33B, RAB3A, RND2, RAB43, RAC3, RAB25, RAB9A, RRAS, RABL5, RAB11A, RAB17, RHOD, RHEBL1, RASL11B, ARHGAP5, RAB1A, RAB32, RAB26, RASD1, IFT27, RASL10A, RAB42, RHOT2, RHOBTB3, RABL2B, hCG, RAB44, RAC1P4, ENSG00000172895 |
| RAG2 | 1 | 1.91 | 8.95108E-07 | RAG2 |
| Requiem_N | 3 | 1.90 | 1.80203E-05 | DPF3, DPF1, DPF2 |
| DUF1075 | 3 | 1.90 | 2.76676E-09 | FAM162A, C3orf28, FAM162B |
| Fox-1_C | 3 | 1.90 | 2.08154E-07 | RBFOX1, RBFOX2, RBFOX3 |
| NYD-SP12_N | 1 | 1.88 | 3.38928E-12 | SPATA16, |
| Lig_chan | 18 | 1.88 | 5.1888E-124 | GRIK2, GRIA2, GRIK1, GRIA3, GRIK3, GRID2, GRIN2A, GRIA1, GRIK5, GRIA4, GRID1, GRIN2B, GRIN3A, GRIN2C, GRIK4, GRIN2D, GRIN3B, GRIN1 |
| Leu_zip | 2 | 1.87 | 1.15799E-10 | LUZP2, LZTFL1 |
| AF-4 | 4 | 1.87 | 8.2459E-99* | AFF2, AFF3, AFF4, AFF1 |
| Sushi | 52 | 1.87 | 0* | CSMD1, CSMD3, CFHR5, CSMD2, CFHR2, CFHR4, CFH, SEZ6L2, CFHR3, MASP1, CR1L, SUSD4, F13B, NCAN, SEZ6L, SEZ6, CR1, C7, PAPPA2, CR2, CD55, PAPPA, SVEP1, ENSG00000165124, C1S, SELE, CFHR1, VCAN, SELL, MASP2, C4BPA, GABBR1, C2, AGC1, ACAN, APOH, SRPX2, C6, FBLN7, BCAN, CD46, SRPX, IL2RA, PAMR1, KIAA0247, CFB, SUSD2, C4BPB, SUSD1, SUSD3, SUSD5, C1R |
| FIIND | 2 | 1.87 | 2.77624E-10 | NLRP1, CARD8 |
| AIG1 | 7 | 1.85 | 2.66804E-44 | GIMAP8, GIMAP7, GIMAP6, GIMAP1, GIMAP4, GIMAP5, GIMAP2 |
| KASH | 5 | 1.84 | 1.24278E-05 | C14orf49, SYNE3, SYNE1, C19orf46, SYNE2 |
| zf-piccolo | 2 | 1.84 | 0.003592513 | PCLO, BSN |
| Maelstrom | 1 | 1.84 | 0.028359979 | MAEL |
| DSPc | 41 | 1.83 | 2.6718E-113* | PTEN, TPTE, TPTE2, ENSG00000197585, Q5JV89, DUSP13, DUSP22, DUSP5, DUSP27, DUPD1, SSH1, DUSP26, DUSP19, PTPDC1, CDC14B, DUSP4, DUSP21, DUSP11, DUSP10, DUSP7, DUSP9, SSH3, DUSP3, STYXL1, DUSP15, CDC14A, DUSP2, EPM2A, SSH2, DUSP6, PTPMT1, STYX, DUSP1, RNGTT, DUSP16, DUSP18, DUSP23, DUSP8, DUSP12, DUSP14, DUSP28 |
| fn1 | 4 | 1.83 | 3.11167E-10 | FN1, HGFAC, PLAT, F12 |
| CUB | 49 | 1.82 | 0* | CSMD3, CSMD1, NETO1, CSMD2, DMBT1, MASP1, NRP2, BMP1, TLL1, KREMEN1, OVCH1, TMPRSS15, PDGFD, SEZ6L2, SEZ6L, TMPRSS7, CUBN, PDGFC, EGFL4, MEGF8, LRP12, DCBLD2, GPR126, PCOLCE2, MASP2, NETO2, CDCP2, SCUBE3, NRP1, TLL2, MFRP, CUZD1, SCUBE2, C1S, TNFAIP6, LRP10, LRP3, C1R, SEZ6, ST14, ATRNL1, PCOLCE, OVCH2, ATRN, DCBLD1, C1RL, PAMR1, SCUBE1, KREMEN2 |
| SPRR2 | 6 | 1.82 | 1.27892E-06 | SPRR2G, SPRR2E, SPRR2B, SPRR2D, SPRR2F, SPRR2A |
| Sp100 | 5 | 1.82 | 3.99941E-09 | SP100, SP140L, SP140, SP110, AIRE |
| DUF3452 | 3 | 1.82 | 8.64117E-07 | RB1, RBL2, RBL1 |
| BRCA2 | 1 | 1.81 | 0.001179986 | BRCA2 |
| zf-HC5HC2H | 11 | 1.81 | 2.47467E-24 | MLL3, PHF6, KMT2D, MLL2, G2E3, MLL4, TCF20, MLL, PHF7, RAI1, PHF11 |
| Gly_acyl_tr_C | 4 | 1.81 | 2.6753E-05 | GLYATL1, GLYATL2, GLYAT, GLYATL3 |
| fn3 | 144 | 1.81 | 0* | PTPRD, TTN, EPHB1, ROBO1, EPHA3, USH2A, NFASC, PTPRB, CNTN4, EPHA6, ENSG00000188974, LIFR, EPHA4, TRIM9, TNR, IGSF9B, INSRR, ROBO2, IL31RA, CNTN5, EGFLAM, PTPRT, PTPRZ1, DCC, PTPRJ, TRIM67, IL7R, TNN, EPHA1, EPHA5, EPHB2, INSR, PTPRU, MYBPC1, DSCAM, CNTN1, NRCAM, CNTN3, SDK1, TEK, EPHA7, PTPRK, COL14A1, CNTN6, COL12A1, ROS1, CHL1, PTPRS, FN1, TYRO3, TNXB, MYOM3, COL20A1, MYBPC2, AXL, EPHA8, TRIM42, MYOM1, L1CAM, IGFN1, LRFN2, TRIM36, FLRT2, ANKFN1, PTPRH, NCAM2, CMYA5, KAL1, IGSF9, MYOM2, TNC, CSF2RB, DSCAML1, IGSF22, SDK2, PTPRF, COL7A1, BOC, CNTFR, ROBO3, CSF3R, FNDC1, EPHB6, TIE1, PTPRM, IL2RG, FNDC7, FNDC3B, IGDCC3, IL6ST, PTPRC, EPHB3, GHR, OSMR, FSD1, PRTG, LRFN1, SORL1, MYLK, FNDC5, CDON, IGDCC4, PHYHIPL, SNED1, PTPRO, CNTN2, LRRN3, EBI3, LRRN4, MYBPHL, NPHS1, OBSCN, EPHA2, IGF1R, IL12RB2, EPHB4, EPHA10, NEO1, FNDC3A, MYBPH, IL27RA, ITGB4, PTPRG, VWA1, FNDC4, LRIT1, ROBO4, MID1, MYBPC3, FSD2, ABI3BP, NCAM1, MERTK, LRRN4CL, FNDC8, LRFN3, IL12RB1, CRLF1, LRFN4, FSD1L, MPL, EPOR, TRAD, KALRN |
| Hormone_2 | 6 | 1.79 | 0.002533294 | GCG, ADCYAP1, VIP, GHRH, GIP, SCT |
| Ig_3 | 60 | 1.79 | 9.52071E-98* | MDGA2, LILRA2, FCRL1, FCRLA, IL1RL1, KIRREL, LILRA1, FCRL5, KIT, KIR3DL1, FCRL3, SIGLEC9, FCRL2, CD84, LILRA5, NCR1, DSCAM, LILRB2, IL1RAPL2, IGSF11, LY9, LILRB1, CSF1R, CD96, SIGLEC7, LILRA3, IL18R1, LILRB5, IGSF1, SDK1, LILRA6, ALCAM, LILRA4, SLAMF8, FCRL6, SLAMF9, KIR3DX1, SLAMF6, CEACAM5, CEACAM6, LILRB4, VSIG4, KIR3DL3, LILRB3, CEACAM8, BCAM, CNTN2, NRCAM, CEACAM1, IL1R1, SLAMF7, KIR3DL2, MCAM, VTCN1, ICAM5, PDCD1LG2, ICAM3, TARM1, KIR2DL3, C17orf60 |
| VCBS | 3 | 1.78 | 0.000143133 | CRTAC1, ITGA3, ITGA6 |
| Gly_acyl_tr_N | 4 | 1.77 | 1.36582E-13 | GLYATL1, GLYAT, GLYATL2, GLYATL3 |
| Med12-LCEWAV | 2 | 1.77 | 6.32497E-16 | MED12L, MED12 |
| Pkinase_Tyr | 122 | 1.77 | 0* | BRAF, FGFR2, EGFR, NTRK3, FGFR1, Q7Z2S2, JAK2, ERBB4, EPHB1, KIT, NTRK1, EPHA4, ABL2, INSRR, RET, KDR, FLT3, EPHA7, PTK2B, JAK3, NTRK2, ABL1, FLT4, ROS1, ERBB2, EPHA5, FGFR3, TNK2, TEX14, PDGFRA, MAP3K13, MET, DDR1, EPHA3, ALK, TYRO3, AXL, NPR2, DDR2, ROR2, EPHB2, TEK, TNNI3K, SYK, LCK, HCK, MUSK, EPHB3, MATK, PDGFRB, KSR1, MST1R, CSF1R, NPR1, FYN, ZAP70, LMTK3, LMTK2, KIAA1804, ENSG00000143674, INSR, KSR2, MAP3K7, LIMK1, MAP3K12, LTK, FES, FLT1, GUCY2F, TIE1, ITK, ERBB3, FRK, EPHA1, GUCY2C, JAK1, EPHA6, RAF1, ENSG00000188974, MERTK, EPHA2, ROR1, EPHA10, BLK, STYK1, EPHB6, LIMK2, EPHA8, TXK, BTK, FER, LYN, MLKL, BMX, MAP3K10, MAP3K11, GUCY2D, ANKK1, TYK2, TEC, FGFR4, FGR, IGF1R, PTK2, TESK2, EPHB4, CSK, ZAK, ENSG00000091436, FLJ23356, ENSG00000185900, PTK7, ILK, SRMS, AATK, YES1, SRC, ARAF, PTK6, TNK1, RYK, ENSG00000183317 |
| DPPIV_N | 6 | 1.77 | 4.79634E-50 | DPP10, DPP6, FAP, DPP4, DPP8, DPP9 |
| FAM131 | 3 | 1.76 | 3.00168E-12 | FAM131B, FAM131A, FAM131C |
| PDEase_I | 21 | 1.76 | 3.29231E-88 | PDE4D, PDE1C, PDE1A, PDE4B, PDE2A, PDE4A, PDE10A, PDE7A, PDE3A, PDE4C, PDE8B, PDE1B, PDE9A, PDE6C, PDE7B, PDE5A, PDE6A, PDE3B, PDE11A, PDE6B, PDE8A |
| ECM1 | 1 | 1.76 | 4.53514E-08 | ECM1 |
| FAD_binding_3 | 7 | 1.76 | 1.1977E-13 | KMO, MICA3, MICAL3, ENSG00000093100, COQ6, MICAL2, MICAL1 |
| hSH3 | 3 | 1.76 | 0.027313763 | FYB, C1orf168, PRAM1 |
| IL6Ra-bind | 11 | 1.75 | 8.64442E-18 | CSF2RA, IL5RA, IL13RA2, IL2RG, IL31RA, IL3RA, IL6ST, CSF2RB, IL6R, IL12RB2, IL13RA1 |
| SPAN-X | 11 | 1.75 | 9.99784E-17 | SPANXD, SPANXN2, SPANXN3, SPANXA1, SPANXA2, SPANXN1, SPANXC, SPANXN5, SPANXN4, SPANXB1, SPANXB2 |
| DUF4589 | 2 | 1.75 | 1.32519E-06 | PRR16, C15orf59 |
| Tho2 | 1 | 1.74 | 0.002749418 | THOC2 |
| WGR | 3 | 1.73 | 0.03743242 | PARP1, PARP3, PARP2 |
| Urocanase | 1 | 1.73 | 5.20545E-08 | UROC1 |
| 7tm_4 | 431 | 1.72 | 0* | OR2L13, OR4A15, OR5L1, OR2G6, OR52R1, ENSG00000176937, OR4M2, OR2T12, OR8U1, OR2M3, OR5D14, OR4K1, OR5J2, OR2T4, OR2M2, OR2L3, OR2M5, OR4C15, OR5D18, OR1L8, OR2M1P, OR4N2, OR14C36, OR5D13, OR5W2, OR11L1, OR2L8, OR2T33, OR52L1, OR2W3, OR5L2, OR4M1, OR6K3, OR2L2, OR8J3, OR5M3, OR2T3, OR4A5, OR10AG1, OR5F1, OR4K2, OR4K5, OR6N1, OR2M4, OR52J3, OR8H2, OR4D10, OR2AK2, OR8H3, ENSG00000187080, OR13C5, OR2B11, OR2T6, OR2A5, OR5T1, OR6V1, OR8I2, OR4C6, OR10G8, OR4A16, OR5AS1, OR10S1, OR10G9, OR4Q3, OR8K3, OR2M7, OR2T34, OR51B2, OR4C13, OR5T3, OR4N4, OR5M9, OR6T1, OR6K2, OR2T8, OR6K6, OR4C16, OR5D16, OR5R1, OR4D5, OR10G7, OR5M1, OR52M1, OR8H1, OR51G1, OR5AR1, OR2J2, OR2J1, OR52B2, OR10Z1, OR10J3, OR2C3, OR4C12, OR5M8, OR8D4, OR5I1, OR10Q1, OR5H15, OR6F1, OR5T2, OR10G4, OR4L1, OR2A14, OR5M11, OR5B12, OR5H1, OR51B4, OR10K1, OR4C3, OR4A47, OR4K13, OR5H2, OR5K3, OR5K1, OR2J3, OR5AC2, OR5H6, OR10T2, OR2G2, OR13G1, OR2T1, OR51B6, OR51Q1, OR52E4, OR8K5, OR5B3, OR2A25, ENSG00000175143, OR51I1, OR8J1, OR52A5, OR13C8, OR7D4, OR10K2, OR4C46, OR1S1, OR56A3, OR2A2, OR51E2, OR14A16, OR51F1, OR4X1, OR51M1, OR10R2, OR5B17, OR10H4, ENSG00000198965, OR6A2, OR10X1, OR2T10, OR52K1, OR51L1, OR5AP2, OR4N5, OR7G1, OR13C2, ENSG00000186400, OR56A4, OR51G2, OR2G3, OR8K1, OR11H12, OR4K15, OR5B2, OR4X2, OR6B1, OR6Y1, OR14I1, OR10A2, OR9G4, OR8D2, OR10P1, OR2Z1, OR10C1, OR13C4, OR3A2, OR4C11, OR1C1, OR52E2, OR52A1, OR52E8, OR10A4, OR4P4, OR4D11, OR8D1, OR4K14, OR10G2, OR1L3, OR52H1, OR6N2, OR51I2, OR9Q1, OR1S2, OR5B21, OR13F1, OR4S1, OR9K2, OR2B6, OR10J5, OR2F1, OR8B8, OR4S2, OR5AK2, OR9Q2, OR5A1, OR1N2, OR52D1, OR2K2, OR5H14, OR52N1, OR2B2, OR5P2, OR6X1, OR6M1, OR4D2, OR52B4, OR52E6, OR5K2, OR4E2, OR51B5, OR4D6, OR10A7, OR7D2, OR13C3, OR1B1, OR2A12, OR10A6, OR4B1, OR8B4, OR1Q1, OR10H2, OR2T11, OR9G1, OR6C74, OR4K17, OR1M1, OR10H5, OR14J1, OR9A4, OR4F6, OR7C2, OR51F2, OR52N2, OR56A1, OR11G2, OR6C75, OR5P3, OR7A5, OR1J4, OR5V1, OR10A5, OR10W1, OR10H1, OR2D3, OR4F15, OR5K4, OR10A3, OR1A1, OR52K2, OR6C1, OR11H4, OR1L4, OR2F2, OR2A7, OR6C3, OR52N4, OR3A1, OR52N5, OR56B4, OR2D2, OR6Q1, OR4D9, OR6C2, OR52B6, OR12D3, OR1F1, OR11H6, OR1J2, OR1A2, OR56B1, OR6C76, OR7E24, OR51D1, OR2AG2, OR6C6, OR1I1, OR2H2, OR1J1, OR6P1, OR13D1, OR5AN1, OR5A2, OR5AU1, OR3A4, OR2B3, OR1L1, ENSG00000173679, OR5M10, OR3A3, OR8B12, OR2V2, OR2H1, OR2S2, OR1E1, OR6C68, OR6C4, OR1K1, Q8NH95, OR52A4, OR2Y1, OR13H1, OR8A1, OR7G2, OR11H1, OR13C9, OR7C1, OR13A1, OR10G3, OR12D2, OR1N1, OR8B2, OR8B3, OR4D1, OR10H3, OR9I1, OR6C65, OR5C1, OR2T27, OR10V1, OR4C5, OR2W1, OR7A17, OR10AD1, OR8S1, OR9A2, OR7G3, OR1D2, OR11A1, OR1G1, OR2AG1, OR6C70, OR6S1, OR2L1P, OR2T2, OR13J1, OR1L6, ENSG00000171459, OR52W1, OR2AE1, OR2C1, OR7A10, OR4A13P, OR2V1, OR1E2, Q8NGM0, OR6B2, OR14K1, OR6B3, OR5D3P, Q8NH98, OR5AX1, OR14A2, OR2T35, OR4F5, LOC651503, O52L2, Q8NGP7, OR2A4, OR2AJ1, Q8NGP5, Q8NH80, OR2A1, OR2A42, OR2T29, OR4F4, O10D4, OR2AP1, OR51J1, Q8IXE7, Q8NH47, OR2T5, OR6W1P, OR6J1, Q8NGG1, Q8NH33, Q8NHC2, OR7A2P, OR4F29, OR4F16, OR4F3, OR10G6, OR5E1P, Q8NGE6, Q8NH06, Q8NH71, Q8NH77, Q8NHC1, ENSG00000187900, ENSG00000204702, OR4F17, O5AK3, OR51H1P, Q8NGQ7, Q8NGY4, Q8NH68, Q8NHB0, Q8NHB5, ENSG00000174126, Q8NGC8, Q8NGK8, Q8NGM4, Q8NH46, ENSG00000180494, ENSG00000198615, Q8NGD7, Q8NGF2, Q8NHA6, OR4F21, Q8N164, Q8NGM6, Q8NH08, Q8NH58, Q8NH75, Q8NH88, ENSG00000168113, ENSG00000183122, ENSG00000196454, ENSG00000198760, Q8NGP1, O10J6, OR10R3P |
| Cu2_monoox_C | 3 | 1.72 | 6.46855E-06 | MOXD1, PAM, DBH |
| Sarcoglycan_1 | 4 | 1.72 | 1.55361E-15 | SGCZ, SGCG, SGCD, SGCB |
| ETS_PEA3_N | 3 | 1.70 | 1.6747E-14 | ETV1, ETV5, ETV4 |
| Plectin | 6 | 1.70 | 6.96267E-38 | PLEC, DST, DSP, EVPL, MACF1, PPL |
| DCX | 8 | 1.69 | 1.36902E-17 | DCLK1, DCX, RP1L1, DCLK2, RP1, DCDC2, DCDC2B, DCDC2C |
| zf-met | 24 | 1.69 | 2.6383E-15 | ZNF385A, ZNF385, ZNF385D, ZMAT4, ZMAT1, ZNF385B, SCAPER, PRDM5, ZFHX4, ZMAT3, BNC1, ZNF131, PRDM10, ZFR, ZNF346, ZBTB32, ZNF800, LOC168850, ZFHX3, ZFR2, BNC2, SF3A2, ZNF574, TUT1 |
| FAM124 | 2 | 1.68 | 5.31083E-05 | FAM124B, FAM124A |
| Integrin_b_cyt | 6 | 1.68 | 0.023539043 | ITGB1, ITGB3, ITGB6, ITGB7, ITGB2, ITGB5 |
| Cadherin_pro | 7 | 1.68 | 3.22924E-07 | DSC3, DSC2, CDH13, DSC1, CDH1, CDH2, CDH4 |
| Involucrin | 1 | 1.67 | 0.003759117 | IVL |
| Tubulin | 26 | 1.67 | 3.61608E-84 | ENSG00000173213, ENSG00000196230, TUBB4Q, ENSG00000254381, TUBA3C, TUBA3D, TUBA4A, TUBB8, TUBE1, TUBAL3, TUBB4, TUBA3E, TUBB1, TUBB3, TUBD1, TUBA1B, TUBA8, TUBA1A, TUBB2B, TUBA1C, TUBB6, TUBG2, TUBB2A, TUBG1, TUBB2C, TUBB |
| Fib_alpha | 3 | 1.67 | 0.000275614 | FGA, FGG, FGB |
| SSFA2_C | 3 | 1.66 | 3.61261E-05 | LOC223075, CCDC129, SSFA2 |
| Mesothelin | 1 | 1.66 | 4.93466E-07 | MSLN |
| Ferritin | 5 | 1.66 | 4.44888E-08 | FTHL17, FTMT, FTL, ENSG00000171878, FTH1 |
| Meckelin | 1 | 1.66 | 6.23666E-10 | TMEM67 |
| EVC2_like | 1 | 1.66 | 0.000772011 | EVC2 |
| Ion_trans | 108 | 1.65 | 0* | CACNA1C, SCN7A, TPTE, CACNA1E, KCNH7, CACNA1I, KCNMA1, CACNA1H, KCNQ5, KCNH5, CACNA1G, SCN2A, SCN1A, NALCN, KCNC2, HCN1, SCN3A, ITPR1, SCN9A, SCN5A, TPTE2, ENSG00000197585, Q5JV89, KCND3, KCNH2, KCNQ2, KCND2, TRPC7, ENSG00000254809, SCN10A, KCNU1, TRPA1, KCNV1, KCNB2, CNGA2, TRPC3, CNGA4, SCN11A, TRPC5, CACNA1D, KCNC1, KCNA3, HCN4, KCNC4, KCNQ3, KCNH1, TRPM2, KCNA6, CNGA3, TRPV5, KCNH6, SCN8A, KCNA1, TRPC4, CACNA1S, CACNA1B, RYR2, KCNS2, KCNA5, KCNH8, CACNA1A, TPCN1, Q6YL47, KCNA10, KCNB1, TRPC6, KCNQ1, KCNG2, ITPR2, ITPR3, SCN4A, TRPC1, KCNA4, CACNA1F, KCNF1, TRPV6, KCNH4, KCNS3, KCNV2, KCNA2, CNGA1, HCN3, KCNC3, HCN2, RYR3, TRPM3, KCNG1, CATSPER4, KCNA7, TRPM1, CATSPER1, TPCN2, KCNG4, TRPV4, KCNQ4, KCNH3, RYR1, CATSPER3, HVCN1, TRPM7, KCND1, TRPM8, TRPV2, TRPV3, CATSPER2, KCNS1, KCNG3, TRPM4 |
| Glyco_transf_41 | 1 | 1.65 | 0.000243669 | OGT |
| DAG_kinase_N | 3 | 1.65 | 0.000737932 | DGKB, DGKG, DGKA |
| PYRIN | 23 | 1.65 | 3.14558E-25 | PYHIN1, MNDA, NLRP12, NLRP13, NLRP4, NLRP9, NLRP8, NLRP10, NLRP3, NLRP14, NLRP5, NLRP7, MEFV, NLRP11, AIM2, PYDC2, IFI16, PYDC1, NLRP2, NLRP1, PYCARD, NLRP6, NALP6 |
| Elongin_A | 4 | 1.65 | 0.001798776 | TCEB3B, TCEB3C, TCEB3CL, TCEB3CL2 |
| PAS_9 | 10 | 1.64 | 2.26797E-12 | KCNH7, KCNH5, KCNH8, PDE8B, KCNH1, PASK, KCNH6, KCNH2, KCNH3, KCNH4 |
| CCDC144C | 29 | 1.64 | 2.71518E-84 | ENSG00000158185, ENSG00000174501, ANKRD36C, ANKRD20A4, ANKRD30A, ANKRD36, ANKRD30B, ANKRD26, CCDC144B, CCDC144A, POTEF, LOC728378, ANKRD18B, ANKRD18A, CCDC144C, ANKRD20A1, POTE2, ENSG00000188219, POTED, ENSG00000180882, ANKRD36BP1, POTEI, ANKRD62, POTEJ, POTEB, ENSG00000230031, ANKRD20A2, ANKRD20A3, AC097374 |
| MACPF | 11 | 1.63 | 1.04334E-25 | FAM5C, C8A, C8B, C9, C6, DBC1, C7, FAM5B, MPEG1, ASTN2, PRF1 |
| ZU5 | 11 | 1.63 | 2.06296E-15 | ANK3, ANK1, UNC5D, ANK2, UNC5C, UNC5A, UNC5B, UNC5CL, PIDD, SH3BP4, TJP1 |
| PAX | 9 | 1.62 | 2.15062E-12 | PAX1, PAX3, PAX4, PAX5, PAX7, PAX6, PAX9, PAX8, PAX2 |
| EphA2_TM | 16 | 1.62 | 8.41199E-15 | EPHB2, EPHA4, EPHB1, EPHA3, EPHA5, EPHA6, EPHA7, ENSG00000188974, EPHA1, EPHB6, EPHA8, EPHA10, EPHA2, EPHB3, EPHB4, ENSG00000183317 |
| IL1 | 9 | 1.62 | 3.83887E-11 | IL37, IL1RN, IL1F10, IL36G, IL36A, IL36RN, IL1A, IL1B, IL18 |
| NCD3G | 13 | 1.62 | 1.11042E-07 | GRM3, GRM8, GRM5, CASR, GRM4, GRM1, GRM7, GRM6, GPRC6A, TAS1R2, TAS1R1, TAS1R3, GRM2 |
| Xlink | 15 | 1.62 | 2.5995E-31 | AGC1, ACAN, HAPLN1, BCAN, NCAN, VCAN, TNFAIP6, HAPLN4, STAB1, STAB2, HAPLN3, CD44, LYVE1, HAPLN2, SUSD5 |
| FMN_dh | 2 | 1.62 | 1.80907E-06 | FMN_dh |
| GCR | 1 | 1.61 | 0.018591325 | NR3C1 |
| Caldesmon | 2 | 1.61 | 1.21737E-08 | CALD1, LSP1 |
| WSC | 5 | 1.61 | 8.9034E-05 | WSCD2, WSCD1, KREMEN1, KREMEN2, PKD1 |
| DUF4457 | 1 | 1.60 | 3.54034E-08 | KIAA0556 |
| Cast | 2 | 1.60 | 5.92621E-17 | ERC2, ERC1 |
| GSDH | 3 | 1.60 | 3.81964E-09 | HHIPL2, HHIPL1, HHIP |
| PI-PLC-Y | 15 | 1.59 | 6.50973E-21 | PLCL1, PLCH1, PLCG2, PLCB1, PLCB4, PLCE1, PLCZ1, PLCL2, PLCB2, PLCG1, PLCD1, PLCD4, PLCB3, PLCD3, PLCH2 |
| ADAM_spacer1 | 22 | 1.59 | 4.18893E-31 | ADAMTS16, ADAMTS20, ADAMTS18, ADAMTS19, ADAMTS8, ADAMTS12, ADAMTS4, PAPLN, THSD4, ADAMTS5, ADAMTS2, ADAMTS1, ADAMTS10, ADAMTS6, ADAMTS3, ADAMTS9, ADAMTS15, ADAMTS17, ADAMTSL2, ADAMTSL4, ADAMTS14, ADAMTS7 |
| COLFI | 11 | 1.59 | 2.50398E-24 | COL11A1, COL2A1, COL3A1, COL5A2, COL1A2, COL5A1, COL5A3, COL1A1, COL27A1, COL24A1, COL11A2 |
| HNOBA | 5 | 1.58 | 9.18297E-06 | GUCY1A2, GUCY1A3, GUCY1B3, GUCY2F, GUCY2D |
| Far-17a_AIG1 | 3 | 1.57 | 0.000117348 | C6orf105, ADTRP, AIG1 |
| Sterol-sensing | 6 | 1.57 | 1.07683E-07 | PTCH1, NPC1L1, PTCH2, NPC1, HMGCR, SCAP |
| MYT1 | 3 | 1.56 | 4.37097E-08 | MYT1L, ST18, MYT1 |
| HCO3_cotransp | 10 | 1.56 | 6.33093E-52 | SLC4A8, SLC4A10, SLC4A4, SLC4A5, SLC4A11, SLC4A7, SLC4A9, SLC4A3, SLC4A2, SLC4A1 |
| BNIP2 | 4 | 1.54 | 0.000140348 | ATCAY, PRUNE2, BNIPL, BNIP2 |
| UvrD-helicase | 3 | 1.52 | 6.44525E-07 | Q8N8K0, TRANK1, FBXO18 |
| V1R | 3 | 1.52 | 6.53519E-05 | VN1R2, VN1R4, VN1R1 |
| TF_AP-2 | 5 | 1.52 | 3.71902E-07 | TFAP2A, TFAP2D, TFAP2B, TFAP2C, TFAP2E |
| UT | 2 | 1.51 | 7.15664E-06 | SLC14A1, SLC14A2 |
| DUF3518 | 2 | 1.51 | 0.031125868 | ARID1A, ARID1B |
| Peptidase_M13_N | 7 | 1.51 | 2.71908E-23 | ECE2, KEL, MME, PHEX, ECE1, ECEL1, MMEL1 |
| EF-hand_like | 15 | 1.50 | 3.84001E-09 | PLCG2, PLCL1, PLCZ1, PLCB1, PLCB4, PLCH1, PLCL2, PLCE1, PLCB2, PLCD1, PLCG1, PLCD3, PLCB3, PLCD4, PLCH2 |
| Neur_chan_memb | 46 | 1.50 | 2.27095E-79 | GABRB3, GABRB2, GABRQ, GABRA4, CHRNA4, GABRB1, GABRG2, HTR3A, GABRA6, GABRA1, HTR3D, GABRA2, GLRB, CHRNB4, CHRNA9, GABRG1, GABRE, GLRA2, GLRA3, GABRG3, CHRNA3, HTR3E, CHRNB2, CHRNB3, GABRA3, CHRND, CHRNA1, GABRR1, CHRNA2, CHRNA6, GABRD, GABRR2, GABRA5, GLRA4, CHRNA10, CHRNG, GABRP, CHRNA7, CHRNB1, GLRA1, HTR3C, CHRNE, CHRNA5, HTR3B, CHRFAM7A, GABRR3 |
| DUF3776 | 3 | 1.50 | 0.041124787 | PHF20L1, Q86U89, PHF20 |
| Ig_2 | 188 | 1.49 | 1.8014E-237* | PSG1, TTN, MDGA2, FGFR2, PSG8, FLT1, PSG4, FCRL1, LILRB5, PSG6, IGSF3, MXRA5, LILRB3, CNTN5, FCRL3, KIRREL2, PSG2, DCC, FLT4, FCRL5, CADM3, NPHS1, FCRLA, FCRL2, IL1RL2, IGSF1, CD226, LILRA1, CNTN4, IL18RAP, KIT, PSG5, SIGLEC10, LILRB1, SIGLEC12, KIRREL, PSG11, PSG9, KDR, CNTN1, IL1RAPL1, IL1RL1, FSTL5, PSG3, LILRB2, KIR2DL1, HEPACAM2, MDGA1, MAG, LILRA5, KIR2DL3, SIGLEC14, IGSF9B, ENSG00000197865, TIE1, FCRL4, FCER1A, CEACAM5, LILRA3, NCR1, VSIG1, DSCAM, NFASC, LILRA6, SIGLEC5, DSCAML1, LILRA2, VSIG10L, ALPK3, MUSK, IGSF21, OSCAR, FCAR, A1BG, SIGLEC8, SDK1, VCAM1, LAIR2, CNTN3, ADAMTSL3, PTGFRN, NRCAM, TMIGD2, NTRK1, PTPRK, ADAMTSL1, IL1RAPL2, IL18R1, CD86, IL1RAP, LAIR1, MYOM3, EMB, NEO1, PVRL1, IL1R2, CHL1, PVRL3, BOC, SIGLEC9, VSTM1, LILRA4, FCGR3B, KIR3DX1, SIGLEC1, GPA33, AMIGO2, CEACAM7, KIR3DL2, CEACAM16, FCGR2B, HEPACAM, IGSF5, TYRO3, F11R, GPR116, NPTN, LILRB4, FCRL6, CEACAM21, KIR2DL4, L1CAM, CDON, JAM3, CD22, BCAM, HSPG2, CEACAM18, BSG, ALCAM, CD96, PDGFRA, FCGR1A, CILP, CD19, FCGR2A, FCRLB, MCAM, PTK7, CD101, LAG3, SEMA7A, ICAM5, CEACAM6, CEACAM8, TMIGD1, VSIG10, CD244, IL1R1, ESAM, ISLR2, KIRREL3, AGER, IGSF9, PDGFRL, IGDCC4, ICAM1, CILP2, CEACAM1, GP6, TARM1, SIGLEC16, PDGFRB, SIGLEC7, FSTL4, AXL, MERTK, PDCD1LG2, MALT1, CLMP, KIR3DL3, FCGR1B, BTLA, SDK2, IGLON5, CNTN2, ICAM3, MYOM2, CD48, LRIG2, CXADR, MMP23B, SIGLEC15, CADM4, OBSL1, C17orf60, IGSF23, ENSG00000215428 |
| Tyrosinase | 3 | 1.49 | 0.002233787 | TYR, DCT, TYRP1 |
| Myelin_PLP | 3 | 1.49 | 0.000728125 | GPM6A, GPM6B, PLP1 |
| SRCR | 19 | 1.49 | 3.06464E-65 | DMBT1, CD5L, CD163, CD163L1, MARCO, LOXL2, MSR1, CD6, LOXL4, SRCRB4D, LOXL3, SCARA5, CD5, PRSS12, LGALS3BP, CFI, HHIPL1, SSC5D, ENSG00000214279 |
| KCNQ_channel | 5 | 1.49 | 8.72894E-08 | KCNQ5, KCNQ3, KCNQ2, KCNQ4, KCNQ1 |
| Lipase | 9 | 1.48 | 2.31674E-21 | PNLIP, LIPI, PNLIPRP3, PNLIPRP1, LPL, PLA1A, LIPH, LIPG, LIPC |
| PLC-beta_C | 3 | 1.48 | 0.038193107 | PLCB1, PLCB2, PLCB3 |
| Glyco_hydro_31 | 6 | 1.48 | 1.76378E-38 | MGAM, SI, GANAB, GAA, GANC, KIAA1161 |
| Serum_albumin | 4 | 1.48 | 9.48204E-13 | GC, ALB, AFM, AFP |
| TB | 7 | 1.47 | 2.33537E-11 | FBN2, LTBP1, LTBP2, FBN3, FBN1, LTBP4, LTBP3 |
| DUF1041 | 5 | 1.47 | 0.002314353 | UNC13C, CADPS, UNC13A, UNC13B, CADPS2 |
| Neur_chan_LBD | 47 | 1.47 | 7.51368E-72 | GABRB3, GABRB2, GABRG1, GABRA6, GABRA2, GABRA5, GABRA1, GABRG2, GLRA2, GLRA4, GABRG3, CHRNB3, GABRB1, GLRB, GABRA4, GABRE, GABRA3, GABRP, HTR3A, GLRA1, CHRND, CHRNA6, GABRQ, GLRA3, GABRR3, CHRNB2, HTR3C, CHRNB4, CHRNA4, HTR3B, CHRNA1, CHRNA9, CHRNA5, HTR3E, GABRR1, CHRNA2, CHRNA3, GABRR2, CHRNB1, GABRD, CHRNA10, CHRNG, CHRNE, CHRNA7, HTR3D, ZACN, CHRFAM7A |
| SEA | 22 | 1.47 | 1.59717E-41 | MUC16, ENSG00000232861, UMODL1, IMPG1, TMPRSS11A, TMPRSS11B, GPR110, TMPRSS7, TMPRSS15, TMPRSS11D, TMPRSS11E, TMPRSS11E2, IMPG2, TMPRSS11F, MUC13, MUC17, TMPRSS6, GPR116, ST14, AGRN, MUC12, MUC1 |
| O-FucT | 2 | 1.45 | 0.011376175 | POFUT2, POFUT1 |
| Tubulin_C | 25 | 1.45 | 1.02568E-19 | TUBA3C, TUBA3D, ENSG00000173213, ENSG00000196230, TUBB4Q, ENSG00000254381, TUBA3E, TUBB8, TUBAL3, TUBB1, TUBB4, TUBB3, TUBB2C, TUBB6, TUBA8, TUBA4A, TUBA1A, TUBA1C, TUBG1, TUBG2, TUBA1B, TUBE1, TUBB, TUBB2A, TUBB2B |
| DNA_pol_B_exo1 | 4 | 1.45 | 5.45918E-08 | POLE, POLD1, REV3L, POLA1 |
| zf-C4 | 47 | 1.45 | 1.32979E-25 | HNF4A, Q8N8C9, ESR2, HNF4G, NR4A3, RARB, NR1I3, NR1H4, THRA, ESRRB, RORA, ESRRG, NR5A2, RORB, NR6A1, RXRG, NR2F2, NR3C1, NR4A2, RARG, THRB, ESR1, NR4A1, AR, PGR, PPARA, NR2C1, RORC, NR1I2, NR1D2, RARA, VDR, NR1D1, NR2C2, NR2F1, PPARD, NR2E3, NR1H3, RXRA, NR2E1, RXRB, PPARG, NR3C2, NR1H2, NR2F6, NR5A1, ESRRA |
| zf-C2H2_2 | 8 | 1.45 | 3.13572E-08 | TSHZ2, TSHZ3, ZFHX4, TSHZ1, DNAJC21, ZNF277, ZNF622, ZFHX2 |
| Laminin_N | 16 | 1.45 | 4.99285E-27 | NTNG1, USH2A, NTNG2, LAMA2, LAMB4, LAMA1, LAMA3, NTN4, LAMB1, LAMB2, LAMB3, LAMC3, NTN1, LAMC1, LAMA5, NTN3 |
| Glyco_hydro_56 | 5 | 1.44 | 4.24305E-09 | SPAM1, HYAL4, HYAL3, HYAL1, HYAL2 |
| NACHT | 28 | 1.44 | 4.93428E-31 | NLRP4, NLRP12, NLRX1, NLRP1, NLRP9, NLRP7, NLRP13, NLRP3, NLRP8, NWD1, NLRP10, NLRP11, NLRP5, NLRC4, CIITA, NLRP14, NLRC5, NOD2, NLRP2, NOD1, NLRC3, NLRP6, NALP6, TEP1, NPHP3, C19orf39, KIAA1239, NAIP |
| DUF1387 | 3 | 1.44 | 0.00075489 | DNAPTP6, SPATS2L, SPATS2 |
| PAN_1 | 10 | 1.43 | 2.79981E-06 | KLKB1, HGF, F11, Q13209, Q49A61, MST1, PLG, MST1P9, PLGLB2, PLGLB1 |
| MIP | 14 | 1.43 | 2.97172E-23 | AQP7, AQP10, AQP8, MIP, AQP9, AQP2, AQP1, AQP3, AQP12B, AQP6, AQP5, AQP12A, AQP4, AQP11 |
| NUC194 | 2 | 1.43 | 0.008737609 | NOL7 |
| V-set | 174 | 1.43 | 9.0339E-134* | PSG1, MGC33530, VSTM2A, VSTM4, HHLA2, PSG8, AGC1, ACAN, SIGLEC6, CADM2, PVRL1, BTNL8, SIGLEC10, SIGLEC12, NCAN, PSG4, IGSF3, VSIG1, SIRPA, AMICA1, TIMD4, HEPACAM2, SIGLEC7, CD33, SIGLEC8, SLAMF6, BCAN, SCN3B, FCAMR, BTN2A2, MOG, SIRPB2, Q5JXA8, CD300E, PVRL3, SIGLEC9, HAPLN1, BTN2A1, TREM2, PSG2, SEMA3D, SIRPD, HAVCR2, PSG6, PSG5, TIGIT, CEACAM4, VPREB1, CD2, VSIG4, TREML2, NCR2, SIRPG, CD48, CD300A, AXL, HEPACAM, CD8B, CRTAM, CD300LG, CEACAM21, CEACAM18, IGSF21, PSG3, NCR3, PSG9, SLAMF9, CD300C, CEACAM7, CEACAM5, JAM2, HAVCR1, IGSF9B, BTNL9, VSTM2L, IGSF8, IGSF11, CD80, CD86, TREM1, PILRB, CD8A, CD300LF, CEACAM3, SIRPB1, CEACAM6, VTCN1, PVRL4, PVRL2, SIGLEC1, CEACAM16, KDR, CXADR, SLAMF7, PIGR, HAPLN4, BTNL3, SIGLEC16, VCAN, CD101, CADM3, GPA33, BTN3A2, MPZL2, JAM3, HAPLN3, CD7, TREML4, VSIG2, CD28, IGLON5, CTLA4, BTN3A1, CEACAM8, PSG11, ICOSLG, SIGLEC11, PTGFRN, CD83, SCN4B, CD4, CD79B, CD300LB, CD79A, C10orf54, BTN3A3, CADM1, CEACAM1, MXRA8, MPZL1, BTNL2, SCN2B, VPREB3, VSIG8, PDCD1, ALCAM, CD300LD, SIGLEC14, ENSG00000197865, C1orf204, ERMAP, F11R, MPZ, BTN1A1, MPZL3, CLMP, CD276, VSIG7, NPHS1, TREML1, PILRA, CD226, CEACAM19, LY6G6F, AMIGO1, IGSF6, MCAM, ENSG00000198229, ENSG00000171101, BTN2A3, SIGLEC5, U66061, VSTM2B, VSIG10, VSTM5, HAPLN2, ESAM, CADM4, PVR, CD274, TAPBPL, SIGLEC15, FAM187A, ENSG00000169664 |
| MAGE | 40 | 1.43 | 2.11188E-43 | MAGEB2, MAGEB6, MAGEB6P1, MAGEE1, MAGEC1, MAGEB10, MAGEA12, MAGEC3, MAGEC2, NDN, MAGEA6, MAGEB4, MAGEA4, TRO, MAGEB1, MAGEB18, MAGEA10, MAGEB3, MAGEA11, MAGEE2, MAGEA1, MAGEA8, MAGEA3, MAGED1, MAGEH1, MAGEL2, MAGED2, MAGEB16, NDNL2, MAGEB5, MAGEF1, MAGED4B, MAGED4, MAGEA13P, ENSG00000183981, MAGEA2B, LOC645864, MAGEB17, MAGEA9, MAGEA9B |
| Collagen | 85 | 1.42 | 6.8973E-166* | COL9A1, COL6A2, MSR1, COL11A1, COL4A5, COL3A1, MARCO, COL21A1, COL22A1, COL20A1, COLQ, COL25A1, COLEC11, COL2A1, COL23A1, COL4A3, COL14A1, COL7A1, COL19A1, COL4A4, MBL2, C1QTNF9, COLEC10, COL4A6, COL24A1, COL13A1, CCBE1, COL1A2, COL28A1, COL5A3, COL4A2, COL6A6, COL5A2, COL15A1, COL4A1, EDA, C1QTNF7, C1QTNF1, COL6A1, ADIPOQ, FCN1, SFTPD, C1QTNF2, GLDN, COL18A1, COL12A1, COL27A1, COLEC12, COL8A1, SCARA3, COL11A2, COL1A1, COL5A1, C1QC, C1QB, COL10A1, FCN2, COL9A3, EMID1, COL16A1, COL9A2, WDR33, OTOL1, CTHRC1, SFTPA2B, C1QTNF9B, SFTPA2, FLJ35880, COL6A5, SCARA5, COL17A1, COL6A3, COL8A2, C1QTNF6, C1QA, FCN3, EMID2, EMILIN1, C1QTNF3, C1QL1, C1QTNF8, SFTPA1, C1QTNF5, C1QL3, C1QL2 |
| zf-C2HC | 6 | 1.42 | 0.011873005 | MYT1L, ST18, MYT1, L3MBTL1, L3MBTL4, MYST2 |
| Reprolysin | 38 | 1.42 | 9.52701E-49 | ADAM12, ADAM22, ADAMTS16, ADAM21, ADAMTS18, ADAMTS20, ADAMTS19, ADAM18, ADAM28, ADAMTS12, ADAM29, ADAMTS5, ADAM19, ADAM7, ADAM2, ADAMTS4, ADAMTS14, ADAMTS8, ADAMTS2, ADAM11, ADAMTS10, ADAM23, ADAMTS6, ADAM30, ADAMTS3, ADAMTS1, ADAMTS7, ADAMDEC1, ADAM15, ADAM32, ADAMTS15, ADAMTS13, ADAMTS9, ADAMTS17, ADAM20, ADAM9, ADAM8, ADAM33 |
| EF-hand_3 | 6 | 1.42 | 0.012958772 | DTNA, DYTN, DMD, UTRN, DTNB, DRP2 |
| DUF1162 | 4 | 1.42 | 0.00464924 | VPS13A, VPS13C, VPS13D, VPS13B |
| UDPGT | 21 | 1.41 | 1.26099E-61 | UGT2B10, UGT2A1, UGT3A1, UGT3A2, UGT2B28, UGT2B4, UGT1A9, UGT2B7, UGT2B11, UGT1A7, UGT2A3, UGT1A10, UGT1A1, UGT1A5, UGT1A3, UGT2B15, UGT8, UGT1A6, UGT1A8, UGT1A4, UGT2B17 |
| EF-hand_2 | 6 | 1.41 | 0.000417565 | DTNA, DMD, DYTN, UTRN, DRP2, DTNB |
| G-gamma | 18 | 1.41 | 0.000151 | RGS7, RGS6, GNG4, GNG2, GNGT2, GNG11, GNG7, GNGT1, GNG5, RGS9, GBG5L, GNG3, RGS11, GNG12, GNG13, GNG10, GNG8, ENSG00000182625 |
| Abhydrolase_3 | 7 | 1.41 | 9.81254E-08 | AADACL2, AADACL3, NCEH1, AADACL4, AFMID, AADAC, LIPE |
| Laminin_G_2 | 40 | 1.41 | 3.91203E-79* | NRXN1, COL11A1, CNTNAP2, CRB1, FAT4, USH2A, CNTNAP5, FAT3, NELL2, NRXN3, EGFLAM, CNTNAP4, NELL1, NRXN2, SLIT2, LAMA5, FAT, FAT1, CELSR1, PROS1, CNTNAP3, SLIT1, CSPG4, SLIT3, LAMA4, COL5A1, LAMA3, COL24A1, GAS6, FAT2, COL11A2, CELSR2, CNTNAP1, CRB2, CNTNAP3B, EYS, CELSR3, LAMA2, Q5T669, HSPG2 |
| OATP | 12 | 1.41 | 2.46963E-37 | SLCO1C1, SLCO1B3, SLCO1B1, SLCO5A1, SLCO4C1, SLCO6A1, SLCO1A2, SLCO1B7, SLCO2B1, SLCO3A1, SLCO2A1, SLCO4A1 |
| FAT | 6 | 1.40 | 1.16866E-10 | ATM, PRKDC, ENSG00000121031, MTOR, TRRAP, ATR |
| Tropomyosin | 5 | 1.39 | 2.73967E-05 | TPM1, TPM3, ENSG00000184673, TPM2, TPM4 |
| 7tm_3 | 22 | 1.39 | 1.36799E-28 | GRM8, GRM7, GRM4, GPR158, GRM5, GRM1, GRM6, GRM3, CASR, GPRC5C, TAS1R2, GRM2, GPRC5B, GPRC6A, GABBR2, GPRC5A, GPRC5D, GPR179, TAS1R1, GPR156, GABBR1, TAS1R3 |
| Neurexophilin | 9 | 1.39 | 2.57648E-07 | FAM55B, FAM55A, NXPE1, NXPH2, NXPH1, FAM55D, NXPH3, FAM55C, NXPH4 |
| IRK | 15 | 1.38 | 1.95216E-23 | KCNJ12, KCNJ3, KCNJ10, KCNJ6, KCNJ4, KCNJ15, KCNJ16, KCNJ5, KCNJ8, KCNJ1, KCNJ2, KCNJ11, KCNJ9, KCNJ14, KCNJ13 |
| DUF3827 | 2 | 1.38 | 0.000225085 | KIAA1549, C11orf41 |
| GCC2_GCC3 | 12 | 1.38 | 0.001108523 | EPHA3, SVEP1, ENSG00000165124, EPHA7, SCUBE2, SCUBE3, EPHB6, TG, EPHB4, SCUBE1, EPHB3, EPHB2 |
| TIG | 25 | 1.38 | 3.38029E-30 | PLXNA4, Q8NAP5, PKHD1, EBF3, PKHD1L1, EBF2, RBPJ, MST1R, PLXNB2, NFATC1, NFATC4, PLXND1, PLXNB3, PLXNA1, CAMTA2, NFATC2, PLXNC1, PLXNA2, MET, PLXNB1, EXOC2, EBF1, PLXNA3, CAMTA1, COE4 |
| ADK | 13 | 1.38 | 4.48529E-18 | AKD1, FLJ42177, ENSG00000188423, AK8, C9orf98, AK2, AK5, AK4, AK3, SPEF2, CMPK1, AK1, C6orf224 |
| SBP_bac_3 | 9 | 1.37 | 4.96343E-13 | GRID2, GRIN2A, GRID1, GRIN2B, GRIN3A, GRIN2C, GRIN2D, GRIN3B, GRIN1 |
| CRAL_TRIO | 13 | 1.37 | 3.09081E-08 | CLVS2, C6orf213, CLVS1, SEC14L5, TTPA, SEC14L1, SEC14L4, SEC14L3, TTPAL, RLBP1, MOSPD2, PTPN9, SEC14L6 |
| Zona_pellucida | 18 | 1.36 | 1.46839E-16 | DMBT1, ZP4, ZPLD1, UMOD, GP2, UMODL1, ZP3, OIT3, CUZD1, ZP2, ZP1, TECTA, TECTB, PLAC1L, TGFBR3, PLAC1, POMZP3, ENG |
| STAS | 11 | 1.35 | 7.46167E-06 | SLC26A7, SLC26A9, SLC26A5, SLC26A8, SLC26A3, SLC26A4, SLC26A6, SLC26A10, SLC26A2, SLC26A1, SLC26A11 |
| T-box | 17 | 1.34 | 7.39394E-11 | TBX5, TBX15, TBX20, TBX1, TBX22, TBX18, TBX3, EOMES, TBR1, TBX10, TBX19, T, TBX21, TBX4, TBX2, MGA, TBX6 |
| MAP2_projctn | 1 | 1.34 | 0.031236395 | MAP2 |
| DNA_methylase | 4 | 1.34 | 0.03062362 | DNMT3A, TRDMT1, DNMT3B, DNMT1 |
| Anoctamin | 10 | 1.34 | 2.35862E-20 | ANO2, ANO4, ANO7, ANO1, ANO6, ANO5, ANO3, ANO9, ANO10, ANO8 |
| Hexokinase_1 | 5 | 1.34 | 5.21634E-05 | GCK, HK3, HK1, HKDC1, HK2 |
| zf-C3HC4_4 | 45 | 1.33 | 5.3772E-05 | SPRYD5, ENSG00000166013, TRIM48, TRIM5, TRIM10, RFPL2, TRIM26, TRIML1, TRIM61, TRIM41, TRIM43, LOC653192, TRIM49, TRIM17, TRIM49L2, TRIM49C, TRIM7, TRIM4, TRIM50, TRIM69, RFPL3, TRIM60, TRIM8, TRIM58, ENSG00000182053, TRIM49B, TRIM39, BFAR, RFPL4B, RFPL1, ENSG00000188683, TRIM6, TRIM25, TRIM31, TRIM68, RFPL4A, RNF39, TRIM34, TRIM62, TRIM11, TRIM22, RNF135, TRIM47, TRIM72, LOC493829 |
| Granin | 3 | 1.33 | 0.000496457 | CHGB, SCG2, CHGA |
| CATSPERD | 2 | 1.32 | 0.005203556 | C1orf101, TMEM146 |
| C2-set_2 | 37 | 1.32 | 1.91586E-11 | CADM2, SIGLEC10, SIGLEC12, KIRREL2, PVRL1, BTN2A1, CADM3, KIRREL3, CD200R1, AGER, BTN2A2, CADM1, CD80, ALCAM, CRTAM, NPHS1, PVRL3, BCAM, MAG, BTNL2, KIRREL, SIGLEC1, CADM4, ICOSLG, BTN3A1, BTN1A1, CD200R1L, PVRL4, CD22, MCAM, CD274, PVRL2, TMEM25, CD276, PVR, SIGLEC11, SIGLEC16 |
| PI-PLC-X | 18 | 1.32 | 5.06383E-08 | PLCB1, PLCE1, PLCXD3, PLCL1, PLCH1, PLCB2, PLCZ1, PLCB4, PLCL2, PLCXD2, PLCG2, PLCD3, PLCB3, PLCD1, PLCH2, PLCXD1, PLCD4, PLCG1 |
| EGF_CA | 78 | 1.31 | 1.95146E-39 | NOTCH1, NOTCH2NL, FBN2, NELL2, FBLN1, TPO, MASP1, LTBP1, NPNT, FAT4, MATN2, CUBN, EGFL6, GAS6, PROS1, CD97, THBS2, NOTCH2, NOTCH3, LTBP4, HMCN1, UMODL1, NID1, LRP1B, NELL1, FBN3, FBLN7, SCUBE1, EFEMP1, EMR1, FBN1, ELTD1, EGF, EFEMP2, EMR2, LTBP2, NOTCH4, MASP2, LRP2, FAT, FAT1, EGFL4, MEGF8, SUSD1, SCUBE2, EMR3, BMP1, LTBP3, CRELD2, FBLN5, CRELD1, MEGF6, FBLN2, THBS1, THBS3, THBS4, CD248, CCBE1, SVEP1, ENSG00000165124, LRP1, HEG1, TLL2, VWCE, CRTAC1, FAT3, COMP, EGFL7, JAG1, JAG2, SCUBE3, VLDLR, UMOD, THBD, EGFL8, EMR4, Q8TCI8, ENSG00000215428 |
| ig | 36 | 1.31 | 3.57826E-06 | NTRK3, SIGLEC6, CSF1R, SIGLEC14, ENSG00000197865, SCN1B, FAIM3, IL1R2, KIR2DL1, KIR3DL1, KIT, CD33, VCAM1, THY1, CD200, SIGLEC8, SIGLEC5, ENSG00000171101, CEACAM1, PVRL2, PTPRM, CD22, MCAM, SEMA3E, FGFR3, FLT3, KIR2DL3, SIGLEC11, PVRL4, C9orf164, SEMA4D, KIR3DL3, PDGFRB, PVR, FCGR1A, SDK2 |
| Disintegrin | 22 | 1.31 | 0.001539958 | ADAM22, ADAM12, ADAM23, ADAM18, ADAM19, ADAM29, ADAM7, ADAM21, ADAM11, ADAM2, ADAM28, ADAM30, ADAM9, ADAM32, ADAMDEC1, ADAM33, ADAM8, ADAM15, ADAM10, ADAM17, ADAM20, ENSG00000196115 |
| Pentaxin | 12 | 1.30 | 3.77488E-05 | CRP, APCS, SVEP1, ENSG00000165124, PTX4, NPTX2, NPTXR, GPR126, GPR112, NPTX1, PTX3, GPR144 |
| CPSase_L_D2 | 7 | 1.30 | 7.02715E-05 | CPS1, ACACA, ACACB, PCCA, MCCC1, CAD, PC |
| An_peroxidase | 10 | 1.30 | 4.61494E-16 | TPO, PXDNL, PTGS2, MPO, PXDN, EPX, PTGS1, LPO, DUOX1, DUOX2 |
| VWA | 45 | 1.30 | 1.16503E-53 | COL6A2, COL6A6, COL21A1, FLJ35880, COL6A5, COL6A3, MATN4, ANTXR1, ITIH5, COL22A1, ITIH2, ANTXR2, COL14A1, VIT, VWA2, COL12A1, ITGAM, COL20A1, ITGA2, CACNA2D1, PARP4, MATN2, VWF, ITGA1, ITIH1, ITGAE, ITGAX, ITGAL, ITGA10, COL6A1, COL28A1, MATN1, COCH, COL7A1, ITGAD, ITGA11, ITIH4, SVEP1, ENSG00000165124, CFB, C2, CACNA2D2, ITIH3, MATN3, VWA1 |
| Calx-beta | 10 | 1.30 | 2.56066E-09 | SLC8A1, SLC8A3, FRAS1, FREM2, FREM1, ENSG00000164946, GPR98, SLC8A2, ITGB4, FREM3 |
| MAGE_N | 26 | 1.30 | 1.75326E-05 | MAGEB2, MAGEB10, MAGEC2, MAGEA12, MAGEB18, MAGEA4, MAGEB6, MAGEB6P1, MAGEB3, MAGEA10, MAGEA5, MAGEB1, MAGEB4, MAGEA11, MAGEA8, MAGEA1, MAGEA6, MAGEB16, MAGEA3, LOC645864, MAGEB17, MAGEA9, MAGEA9B, MAGEA2B, MAGEA13P, ENSG00000183981 |
| cNMP_binding | 34 | 1.29 | 9.60015E-11 | CNBD1, HCN1, PRKG1, CNGA3, KCNH5, KCNH1, PRKG2, PNPLA7, PNPLA6, RAPGEF6, KCNH7, CNGB3, RAPGEF4, CNGA2, KCNH8, SLC9A10, SLC9A11, CNGB1, PRKAR2B, KCNH6, PRKAR2A, KCNH2, C20orf152, PRKAR1A, HCN3, KCNH3, HCN4, CNGA4, RAPGEF2, CNGA1, RAPGEF3, KCNH4, PRKAR1B, HCN2 |
| DUF3497 | 12 | 1.28 | 3.46904E-07 | LPHN3, BAI3, LPHN2, ELTD1, GPR113, BAI1, CELSR3, EMR3, CELSR2, CELSR1, BAI2, LPHN1 |
| Ets | 27 | 1.28 | 0.000654603 | Ets |
| Ion_trans_2 | 22 | 1.28 | 5.44238E-07 | KCNK10, KCNK9, KCNT2, KCNK16, KCNK2, KCNN2, KCNK13, LOC145814, KCNK7, KCNN1, KCNK6, KCNN3, KCNK18, KCNK15, KCNK17, KCNK5, KCNK3, KCNK1, KCNT1, KCNK4, KCNN4, KCNK12 |
| Ten_N | 7 | 1.27 | 4.29344E-05 | ODZ1, ODZ2, TENM2, TENM1, ENSG00000182933, ODZ3, ODZ4 |
| CAP | 15 | 1.27 | 0.011031048 | CRISP3, PI15, GLIPR1L2, CRISP2, CRISP1, CRISPLD1, GLIPR1, GLIPR1L1, R3HDML, PI16, CLEC18B, CLEC18A, CRISPLD2, GLIPR2, CLEC18C |
| RYDR_ITPR | 6 | 1.26 | 0.001504633 | ITPR1, RYR2, ITPR3, RYR3, RYR1, ITPR2 |
| OLF | 13 | 1.26 | 1.53682E-07 | LPHN2, LPHN3, OLFM3, OLFML2B, OLFM4, OLFM1, MYOC, OLFML2A, OLFML3, GLDN, OLFML1, OLFM2, LPHN1 |
| Cystatin | 19 | 1.25 | 0.024621017 | KNG1, CST2, CST1, CSTL1, CST5, CST4, CST6, CST8, CST11, CST9L, CST9, HRG, CSTA, FETUB, AHSG, CST7, CST3, CSTB, CST9LP1, C5orf32 |
| Sulfotransfer_1 | 33 | 1.25 | 1.68391E-17 | HS3ST4, SULT1C2, NDST4, CHST1, CHST2, CHST6, SULT1C3, SULT1A1, CHST5, HS3ST2, HS3ST6, HS3ST5, SULT1E1, DSEL, SULT1B1, CHST4, SULT2B1, SULT1C4, SULT2A1, SULT6B1, HS3ST1, SULT1A2, NDST3, NDST1, CHST7, HS3ST3A1, CHST3, CHST15, SULT4A1, NDST2, HS3ST3B1, WSCD1, SULT1A3 |
| Serpin | 36 | 1.25 | 1.09622E-27 | SERPINA9, SERPINB3, SERPINA6, SERPINB4, SERPINI2, SERPINE2, SERPINA10, SERPINB7, SERPINB2, SERPIND1, SERPINA12, SERPINA3, SERPINA7, SERPINA4, SERPINB13, SERPINA1, SERPINB5, SERPINB10, SERPINC1, SERPINB12, AGT, SERPINA5, SERPINB8, SERPINB11, SERPINI1, SERPINB9, SERPING1, SERPINE1, SERPINF2, SERPINE3, SERPINH1, SERPINB6, SERPINA13, SERPINF1, SERPINB1, HMSD |
| Pyridoxal_deC | 9 | 1.25 | 0.000972585 | DDC, GADL1, GAD2, GAD1, HDC, CSAD, SGPL1, PDXDC1, PDXDC2 |
| DHC_N1 | 10 | 1.25 | 1.69513E-10 | DNAH5, DNAH10, ENSG00000150980, DNAH9, DNAH8, DYNC2H1, DNAH2, DNAH11, DYNC1H1, DNAH17 |
| Na_sulph_symp | 6 | 1.24 | 0.000183979 | SLC13A2, SLC13A1, SLC13A3, SLC13A5, SLC13A4, OCA2 |
| TSP_1 | 58 | 1.24 | 3.04167E-26 | ADAMTS20, ADAMTS16, ADAMTS12, ADAMTS2, C6, THSD7B, SEMA5B, ADAMTS18, HMCN1, ADAMTSL1, ADAMTS19, BAI3, ADAMTS4, C7, ADAMTSL4, SEMA5A, ADAMTSL3, UNC5C, C9, THBS2, ADAMTS14, UNC5B, C8A, PAPLN, THSD7A, ADAMTS13, ADAMTS3, WISP2, UNC5D, ADAMTS6, ADAMTS5, ADAMTS9, BAI1, CFP, ADAMTS10, RSPO3, SPON1, ADAMTS7, THBS1, THSD4, WISP1, ADAMTS17, ADAMTS8, CILP, ADAMTS1, ADAMTS15, CTGF, WISP3, NOV, ADAMTSL2, THSD1, BAI2, RSPO1, ISM1, CYR61, ISM2, CILP2, Q76B61 |
| Hydrolase | 21 | 1.24 | 4.04703E-12 | ATP2B3, ATP2B2, ATP12A, ATP2B4, ATP1A2, ATP1A4, ATP1A3, ATP7A, ATP2B1, ATP2A3, ATP9A, ATP2A1, ATP11B, ATP2C2, ATP4A, ATP1A1, ATP7B, ATP9B, ATP2C1, PSPH, ATP2A2 |
| VWC | 29 | 1.23 | 0.000317961 | FRAS1, NELL2, COL3A1, NELL1, VWC2, WISP1, COL2A1, BMPER, VWF, WISP2, NOV, THBS2, PXDNL, CHRDL1, VWCE, CHRD, CHRDL2, CRIM1, ECM2, MUC5AC, MUC5B, PXDN, THBS1, COL5A2, COL1A1, CTGF, CYR61, Q8NBE0, ENSG00000196292 |
| COesterase | 14 | 1.23 | 2.06886E-14 | COesterase |
| TIR | 20 | 1.22 | 0.006119089 | IL1RAPL1, MYD88, TLR4, IL1RL1, IL1RL2, IL1RAPL2, IL1RAP, TLR3, IL18RAP, TLR9, TLR1, IL18R1, TLR8, TLR7, IL1R1, TLR2, TLR5, TLR10, TLR6, SIGIRR |
| Laminin_G_3 | 14 | 1.22 | 0.006758413 | PAPPA2, USH2A, CLSTN2, GPR133, COL15A1, PAPPA, GPR98, CLSTN3, NBEAL1, CLSTN1, NBEA, NBEAL2, COL18A1, LRBA |
| PI3_PI4_kinase | 21 | 1.22 | 1.7881E-07 | ATM, PIK3CA, MTOR, PIK3C2G, TRRAP, PIK3CG, ATR, PI4K2B, PRKDC, ENSG00000121031, SMG1, PIK3C3, PIK3C2B, PIK3CD, PI4K2A, PIK3C2A, PIK3CB, PI4KA, PI4KB, LOC220686, PI4KAP2 |
| C2 | 123 | 1.22 | 7.99859E-33 | RIMS2, UNC13C, PLCL1, PLCB1, PLCH1, DYSF, PCLO, PRKCB, HECW1, SYT4, SYT15, SYT16, OTOF, PLCB4, CPNE4, MYOF, RASAL2, UNC13A, RIMS1, PRKCG, RGS3, FER1L6, PLCZ1, CCDC33, PLCE1, SYT10, SYT14, PLCG2, RASA2, SYTL1, RIMS4, SYT5, UVRAG, SYT9, ITSN1, MCTP1, CPNE9, SYT1, PLCL2, SYT17, UNC13B, SYT12, TC2N, ABR, MTAC2D1, RASA1, PLCB2, CAPN6, SYTL5, SYT2, RAB11FIP2, BAIAP3, C2CD3, SYTL2, HECW2, NEDD4L, CPNE5, TOLLIP, SYT3, SYT13, RPH3A, WWP1, SYT11, DAB2IP, SYT7, PLA2G4D, SYTL3, ENSG00000189290, RASAL1, ESYT1, ESYT3, PRF1, MCTP2, RASA3, PLA2G4A, PLCH2, CPNE3, PLCD1, CPNE8, CPNE1, RIMS3, CC2D1B, ESYT2, C2CD2, PLCG1, SYTL4, DOC2A, RPGRIP1L, PRKCA, PLCD3, PLCD4, CPNE6, CPNE2, SYNGAP1, PRKCH, PIK3C2B, CPNE7, FER1L5, PLCB3, PIK3C2A, SYT8, UNC13D, KIAA0528, SYT14L, ITSN2, BCR, PRKCE, SMURF1, CC2D1A, SYT6, SMURF2, PLA2G4F, PLA2G4E, ITCH, RASA4, CAPN5, Q5JX50, Q9GZQ9, RAB11FIP1, C2CD4C, C2CD4D, RAB11FIP5, RASL2 |
| Sema | 32 | 1.21 | 3.10197E-21 | PLXNA4, Q8NAP5, SEMA6D, SEMA5B, SEMA6C, SEMA3E, SEMA5A, SEMA3A, SEMA3D, SEMA4F, MST1R, MET, SEMA3C, C9orf164, SEMA4D, SEMA4A, PLXNA3, SEMA3F, SEMA3G, PLXNA2, SEMA6B, PLXNA1, PLXNB2, SEMA7A, PLXNB3, SEMA4G, SEMA4C, SEMA6A, PLXNB1, SEMA3B, SEMA4B, PLXND1 |
| Patched | 10 | 1.21 | 0.000291467 | PTCH1, C6orf138, PTCHD3, PTCHD1, PTCHD2, DISP1, NPC1L1, NPC1, PTCH2, PTCHD4 |
| 7tm_1 | 315 | 1.21 | 1.4565E-122* | ADRA1A, CHRM2, NPSR1, OR51T1, ENSG00000176900, CCKBR, OPRM1, PTGER3, HTR1A, PTGFR, NMUR2, EDNRB, HTR5A, CHRM3, HTR7, OR51S1, NPY5R, HTR2C, HTR4, MCHR2, DRD2, DRD5, CCR5, HTR1E, PROKR2, CCR2, OR51A7, GPR50, ADRA2B, GHSR, HRH2, OR10J1, GPR101, HCRTR2, OR51A4, OPN1LW, CX3CR1, OPRK1, MRGPRX1, LPAR4, GPR174, LHCGR, HTR2A, P2RY8, TSHR, FPR1, P2RY13, OR51V1, P2RY10, GPR45, NPY1R, SSTR4, HRH1, FPR2, GPR109B, HCAR3, HTR1F, MC5R, AGTR1, GPR141, LGR6, MC3R, CCR3, CMKLR1, ADORA3, CXCR7, PPYR1, OR51E1, OR51A2, PROKR1, NPFFR2, CNR1, GPR173, PTGDR, GPR32, OR1D5, DRD3, NTSR2, CCKAR, GPR78, GPR139, CNR2, TACR3, MRGPRX4, P2RY2, AVPR1A, HTR1B, TRHR, S1PR1, OR52I2, S1PR4, NPY2R, EDG6, ADORA1, MTNR1B, FSHR, GPR55, GPR149, GPR6, TAAR6, CHRM5, GPR82, GPR15, CXCR2, NMBR, TAAR5, LPAR1, SSTR1, GPR12, NTSR1, OPN4, F2RL2, GPR161, NPBWR2, AGTR2, FPR3, BRS3, AVPR1B, CCBP2, GPR35, HRH3, CHRM4, TAAR2, GALR1, ADRA2A, FFAR1, P2RY1, MAS1L, GPR26, HRH4, OR2W5, CXCR3, MRGPRX2, GPR142, QRFPR, OPN5, TBXA2R, CXCR4, GPR87, GPR63, GPR85, MRGPRX3, RXFP2, CCR8, CXCR1, EDNRA, RXFP3, APLNR, O3FAR1, GPR39, DRD1, LPAR3, GPR65, GPR37, MRGPRD, F2R, BDKRB2, GRPR, P2RY4, HTR1D, NMUR1, XCR1, P2RY12, OXGR1, MC4R, LGR5, AVPR2, GPR61, PRLHR, P2RY6, GPR83, GPR162, BDKRB1, FFAR2, GPR4, RXFP1, CYSLTR1, GPR75, GPR153, OPRD1, P2RY14, S1PR3, GPR132, MCHR1, F2RL1, PTAFR, MTNR1A, ADRA1B, OPN1SW, GPR84, GPR20, CCR1, CCR4, C5AR1, TAAR1, GPR27, GPR171, NPBWR1, CHRM1, SSTR3, RHO, GPR17, TAAR8, MAS1, CYSLTR2, TACR1, DRD4, P2RY11, LGR4, HCAR2, CCR9, GPR34, GPR1, OXTR, TACR2, SSTR5, SSTR2, GPR77, OR2AT4, ADRA2C, GPR119, HTR6, ADRB2, GPR31, OPRL1, GNRHR, MRGPRE, SUCNR1, RGR, GALR2, ADRA1D, OPN1MW, OPN1MW2, F2RL3, PTGER4, RXFP4, FFAR3, GPR52, GPER, GPR183, OPN3, GPR182, PTGER2, GPR148, LPAR5, PTGIR, NPFFR1, CXCR5, CCR6, GPR37L1, CCRL1, ENSG00000118519, C3AR1, ADRB3, GPR19, Q8N0W1, ENSG00000186483, MC1R, GPR22, HTR2B, ADRB1, CCR7, RRH, GPR21, GPR44, LPAR6, GPR176, OXER1, GPR151, HCRTR1, GPR146, GPR152, GPBAR1, CCRL2, GPR25, HCAR1, LTB4R, GPR3, UTS2R, S1PR5, GPR18, OR7E5P, MLNR, LPAR2, ADORA2B, CXCR6, GALR3, GPR135, MRGPRF, CCR10, KISS1R, GPR88, GPR62, PTGER1, Q8IXE5, GPR42, Q8NHB3, GPR150, ENSG00000197526, ENSG00000198544, DAPL1, MRGPRG, Q8NH32, DRD5P1, ENSG00000176951, Q8NHC0, O60411, Q8NH11, Q8NH82, ENSG00000172764, ENSG00000188604, Q8NHB0, GNRHR2, NPY6R, OR1D4 |
| Fork_head | 49 | 1.21 | 3.34941E-05 | LOC442425, FOXB2, FOXA1, FOXN4, FOXG1, FOXP2, FOXF1, FOXD4, FOXO1, FOXB1, FOXL1, FOXD4L1, FOXK1, KIAA0415, FOXD4L5, FOXI1, FOXR2, FOXA2, FOXO4, FOXP1, FOXN3, FOXD4L4, FOXP4, FOXD4L2, FOXR1, FOXL2, FOXN1, FOXI2, FOXF2, FOXK2, FOXJ2, FOXA3, FOXC2, FOXJ3, FOXQ1, FOXS1, FOXN2, FOXE1, FOXJ1, FOXM1, FOXO3, FOXD4L3, FOXD4L6, FOXE3, FOXD3, FOXP3, FOXD2, FOXH1, FOXO6 |
| Sulfate_transp | 11 | 1.20 | 0.013296856 | SLC26A5, SLC26A9, SLC26A7, SLC26A4, SLC26A3, SLC26A11, SLC26A10, SLC26A2, SLC26A1, SLC26A6, SLC26A8 |
| Filamin | 11 | 1.20 | 8.02623E-08 | MYCBP2, FLNB, FLNA, FLNC, TRIM71, TRIM2, TRIM45, KDELC1, KDELC2, TRIM3, KIAA0317 |
| Pep_M12B_propep | 40 | 1.20 | 6.53736E-06 | ADAMTS16, ADAM22, ADAM21, ADAMTS20, ADAM12, ADAM19, ADAM29, ADAMTS18, ADAMTS5, ADAMTS12, ADAM18, ADAM28, ADAM7, ADAMDEC1, ADAMTS10, ADAM23, ADAM11, ADAMTS3, ADAMTS4, ADAMTS14, ADAMTS2, ADAM2, ADAMTS9, ADAM20, ADAM33, ADAMTS15, ADAM30, ADAM15, ADAMTS1, ADAMTS7, ADAM10, ADAM9, ADAM8, ADAMTS19, ADAMTS6, ADAM17, ADAM32, ADAMTS17, ADAMTS8, ENSG00000196115 |
| Plexin_cytopl | 10 | 1.18 | 0.000251417 | PLXNA4, Q8NAP5, PLXNB3, PLXND1, PLXNA2, PLXNC1, PLXNA1, PLXNB2, PLXNB1, PLXNA3 |
| Homeobox | 201 | 1.18 | 3.09314E-12 | POU3F4, ZFHX4, PRRX1, PAX7, NOBOX, PAX4, ISX, POU4F2, HOPX, HNF1A, PITX2, SATB2, VAX1, HNF1B, POU1F1, HOXD13, HOXA10, LASS3, HOXA1, DUXA, ONECUT2, HOXC13, HOXD9, HOXD4, HMBOX1, EVX2, VSX1, DMBX1, PAX3, HDX, LHX9, HOXA6, HOXA7, DLX5, LHX8, ALX4, ARGFX, SHOX2, CDX4, HOXD11, LMX1A, OTP, GSX1, HOXB13, DBX2, POU4F1, LHX6, HOXD3, HOXD1, PHOX2B, BARHL2, HOXD10, HOXC6, DPRX, OTX2, MEOX2, PBX1, SIX3, VAX2, EN1, GBX2, PROP1, HOXA5, LMX1B, ALX1, VSX2, DLX3, MSX1, BSX, CUX2, SATB1, ZHX1, POU6F1, HESX1, SHOX, POU2F1, MSX2, POU3F2, HOXA4, PHOX2A, BARX2, HOXC4, LBX1, ISL1, CRX, ESX1, HLX, SIX2, OTX1, POU4F3, EN2, HOXC9, HOXC5, SIX6, ZFHX3, UNCX, CUX1, DLX4, ZHX2, CDX2, HOXA11, RHOXF1, BARHL1, ZEB1, HOXC12, LHX5, MEOX1, DBX1, POU6F2, NOTO, POU2F2, CDX1, HOXA3, EMX2, ONECUT1, ADNP, HHEX, PAX6, TLX1, ARX, DLX2, PBX2, LHX3, VENTX, LHX1, HOXB4, HOXB6, HOXB1, POU3F3, GBX1, HOXA13, HOXC8, HOXB7, NANOG, HMX3, GSX2, PBX3, POU2F3, LASS5, SIX1, HOXB5, HOXB8, RAX, POU5F1, HOXA9, HOXC11, LASS2, LHX4, TLX2, HOXD8, HOXA2, EVX1, LHX2, GSC, HOXB2, HOXB9, LASS4, BARX1, HOXD12, DRGX, PBX4, SEBOX, MNX1, TLX3, SIX4, HMX2, PITX1, PITX3, MIXL1, PRRX2, HOXC10, ISL2, HOXB3, RAX2, SIX5, ZHX3, POU3F1, NANOGNB, ANHX, ALX3, RHOXF2, DLX1, RHOXF2B, NANOGP1, ZFHX2, LEUTX, POU5F1B, POU5F2, LASS6, EMX1, GSC2, DUX4, ONECUT3, DUX4L2, DLX6, DUX4L3, LBX2, PDX1, ENSG00000198694, DUX4L4, HMX1 |
| F5_F8_type_C | 25 | 1.18 | 0.002992416 | CNTNAP2, DDR2, F8, CNTNAP5, DDR1, EDIL3, F5, NRP2, CPXM1, CNTNAP4, RS1,CPXM2, BTBD9, ENSG00000181922, AEBP1, MFGE8, NRP1, CNTNAP1, DCBLD1, DCBLD2, NR2C2AP, CNTNAP3B, CNTNAP3, Q76B61, HSPB11 |
| G-alpha | 16 | 1.17 | 0.000251092 | GNAS, GNAT3, GNAO1, GNA13, GNAI2, GNAZ, GNAL, GNAQ, GNA11, GNA14, GNAI1, GNA15, GNAT2, GNAT1, GNA12, GNAI3 |
| TAS2R | 24 | 1.17 | 9.82121E-05 | TAS2R31, TAS2R1, TAS2R46, TAS2R40, TAS2R43, TAS2R16, TAS2R38, TAS2R60, TAS2R30, TAS2R39, TAS2R41, TAS2R19, TAS2R10, TAS2R9, TAS2R7, TAS2R8, TAS2R5, TAS2R14, TAS2R3, TAS2R20, TAS2R50, TAS2R42,TAS2R4, TAS2R13 |
| Glyco_transf_29 | 19 | 1.16 | 0.014870785 | ST6GAL2, ST8SIA4, ST6GALNAC3, ST6GALNAC5, ST8SIA3, ST8SIA5, ST8SIA6, ST3GAL6, ST3GAL5, ST8SIA2, ST8SIA1, ST3GAL3, ST6GALNAC2, ST6GALNAC6, ST6GALNAC1, ST6GALNAC4, ST3GAL4, ST3GAL2, ST6GAL1 |
| Myosin_tail_1 | 18 | 1.15 | 1.37899E-08 | MYH13, MYH1, MYH2, MYH8, MYH4, MYH7, MYH6, MYH14, MYH10, MYH15, MYH11, MYH3, MYH9, MYO18A, MYH7B, CGN, CGNL1, MYH16 |
| Ank | 77 | 1.14 | 0.006581786 | ANKRD30BL, ANK1, CDKN2A, DGKI, DAPK1, TRPA1, ANK2, TRPC5, ASB4, TRPC3, TNKS, KANK1, NOTCH1, PPP1R12B, TONSL, ANKRD28, BARD1, NFKBIE, CDKN2C, ANKS1A, ANKRD24, RNASEL, ANKRD34B, NFKB1, TRPV4, GABPB1, ANKRD27, ANKRD7, ABTB2, CDKN2D, NFKBIB, PPP1R16B, PSMD10, EHMT2, TRPC4, TRPV3, RFXANK, NFKBIA, TP53BP2, ANKHD1, DGKZ, ANKRD13A, FEM1B, ANKRD12, PLA2G6, ACAP2, RAI14, ACAP1, BCL3, KRIT1, PPP1R12A, ASB15, ASB3, ASB8, ANKRD20A1, OSBPL1A, ANKRD20A2, ANKRD20A4, ZDHHC17, ILK, TNKS2, PPP1R13B, GIT2, ANKRA2, CLPB, ANKRD20A3, ENSG00000164236, FANK1, ANKK1, KANK2, NFKB2, AGAP2, ASAP2, FEM1A, ANKZF1, ENSG00000157999, ANKIB1 |
| LRR_8 | 174 | 1.13 | 4.20399E-14 | NTRK3, LRRTM4, LINGO2, PXDNL, LRFN5, LRRTM1, SLIT3, LRRTM3, LRRC15, NTRK2, TLR4, LRTM1, LRRK2, LUM, FLRT2, SLIT2, SLITRK4, FSHR, LRRN3, MXRA5, GP5, SLITRK3, LRRC32, LRIG1, OPTC, LRFN2, SLITRK6, LGR6, LRCH1, LRRC17, KERA, SLITRK2, PHLPP, PODN, LRRIQ4, PHLPP1, ERBB2IP, LRRC55, LRRC18, LRRC1, LRCH2, LRRC3B, SLITRK1, FLRT3, LINGO1, FMOD, ANP32E, LRRN2, IGSF10, LRRC4, AMIGO2, LRIG3, TPBG, ISLR2, ISLR, LRRC27, CPN2, RXFP1, LGI2, DCN, TLR8, LRRC33, RXFP2, CHAD, LRG1, LRTM2, AMIGO3, LRRC8D, TLR6, LGR5, SLITRK5, IGFALS, LGI4, LRFN3, LRRC3, LINGO4, LGR4, ELFN2, ENSG00000183096, LRRC58, LRRC69, LRRK1, TSHR, PRELP, LRCH3, LRIT2, RTN4RL1, LRRN1, BGN, LRRC66, TLR7, TLR9, PIDD, TLR3, LRRC40, LRRC8B, TLR10, LGI1, SLIT1, FLRT1, LRFN4, TSKU, LRRC23, LRRC46, LRRC59, TLR5, B7, LRRC48, TLR1, LRIG2, TLR2, OGN, ASPN, LRRC20, LRRC28, GPR125, PXDN, LRRTM2, ANP32D, ECM2, LRRC4B, CNTRL, NYX, GP9, RTN4RL2, EPYC, LRRC37B, LRCH4, LRFN1, GP1BA, LINGO3, CD180, CNOT6, LRRN4, RTN4R, LRRC8A, SCRIB, RSU1, LRIT1, CHADL, OMD, SHOC2, FLII, OMG, MFHAS1, ENSG00000162621, Q7Z2Q7, LRRD1, LRRC53, ELFN1, LRRC70, PODNL1, LRRC8C, LRIT3, LGI3, GPR124, LRRC19, LRRC57, NISCH, LRRC24, LRRC38, PHLPP2, VASN, LRRC39, CNOT6L, PKD1, LRRC37A, LRRC3C, LRRC63, AMIGO1, LRRC37A3, LRRC26, LRRC37A2, LRRC10B |
| Y_phosphatase | 42 | 1.12 | 6.83156E-06 | PTPRD, PTPRK, PTPRB, PTPRU, PTPRN2, PTPN5, PTPRT, PTPN11, PTPRJ, PTPRZ1, PTPRE, PTPRC, PTPRR, PTPRF, PTPRN, PTPN3, PTPN12, PTPRH, PTPN14, PTPN2, PTPRM, PTPN4, PTPRS, PTPRA, PTPRG, PTPN13, PTPN21, PTPN6, PTPN18, PTPRO, PTPN23, PTPN9, PTPN1, PTPN7, PTP4A3, PTP4A1, PTPN20B, PTPN20A, Q5SWJ0, PTPN20C, PTP4A2, PTPN22 |
| PDZ | 133 | 1.11 | 3.11318E-07 | DLG2, RIMS2, MAGI1, PDZRN4, SNTG1, MPDZ, SDCBP2, PREX2, PARD3B, LIMK2, DFNB31, TIAM1, LMO7, MLLT4, LIN7A, LNX1, MAGI2, MAGI3, SCRIB, USH1C, SNTG2, RAPGEF6, APBA2, NOS1, MPP4, PDZRN3, IL16, PDLIM5, LDB3, GRIP1, RGS3, LRRC7, SYNPO2, RHPN1, MAST1, TJP2, LIMK1, FRMPD2, PARD3, DLG3, HTRA1, MAST3, TIAM2, SHANK2, MYO18A, MAST4, PDZD3, INADL, CASK, PTPN3, MPP2, PRX, TJP3, DLG5, PTPN13, LNX2, DVL1, CNKSR2, SHROOM3, APBA1, MPP7, PDLIM1, PARD6G, PARD6B, SHANK3, GRASP, MAST2, DVL3, CYTIP, DLG1, PDZD7, SLC9A3R2, MAGIX, PDZD4, ARHGAP21, ARHGEF12, GIPC1, PPP1R9A, RGS12, DVL2, GIPC2, SYNJ2BP, DLG4, PICK1, PDLIM3, MPP6, TJP1, RIMS1, SHANK1, FRMPD1, LIN7C, MPP5, STXBP4, GIPC3, MPP3, SNTB1, GOPC, MPP1, PDZD11, PDZK1, SDCBP, LIN7B, RADIL, RAPGEF2, SNX27, SHROOM2, CNKSR3, SYNPO2L, ARHGEF11, SHROOM4, PARD6A, PDLIM7, PDZD9, SLC9A3R1, SNTA1, PPP1R9B, GRID2IP, TAX1BP3, ARHGAP23, SIPA1L2, AC142381, ENSG00000167433, PTPN4, APBA3, PDLIM2, ERBB2IP, FRMPD3, FRMPD4, PDLIM4, SIPA1, SNTB2, FRMPDP2, FRMPD2P1 |
| zf-H2C2_2 | 647 | 1.10 | 5.34643E-37 | ZNF429, ZNF814, ZNF83, ZNF91, ZNF479, ZNF878, ZNF208, ZFP112, ZNF711, ZNF721, ZNF606, ZNF98, ZNF716, ZNF717, PRDM9, WT1, ZNF99, CTCF, ZNF8, ZNF676, ZNF441, ZNF626, ZNF836, ZNF799, ZNF662, ZIC4, ZNF423, ZNF493, ZSCAN1, ZNF37A, ZNF382, ZNF709, ZNF823, ZSCAN5B, ZNF845, ZNF286B, ZFP42, ZNF776, ZNF28, ZIM3, ZNF714, ZNF679, ZNF880, ZNF563, ZNF780A, ZNF236, ZNF486, MTF1, BCL6, IKZF3, ZIC1, ZNF480, ENSG00000197608, ZNF841, ZNF391, ZNF180, ZEB2, ZNF80, PRDM14, ZFP64, ZNF470, ZNF334, ZNF568, ZNF492, ZNF529, ZNF860, ZNF780B, ZNF732, ENSG00000198153, ZNF238, PLAG1, ZNF182, ZNF177, ZNF625, HKR1, ZNF577, ZNF649, ZNF600, ZNF415, ZNF536, ZNF616, ZNF816, ZNF138, ZNF831, ZNF665, ENSG00000221895, ENSG00000257591, ZNF678, ZIC3, SALL3, ZNF167, ZFP2, ZNF454, ZFP37, SALL1, ZNF560, ZNF442, ZNF573, ZNF677, ZNF256, ZNF135, ZNF33A, ZNF443, ZNF607, ZNF827, ZNF425, ZNF229, ZNF835, LOC152485, ZNF737, ZNF681, ZNF853, ZNF790, ZNF569, ZNF519, KLF3, ZNF275, ZNF436, ZNF643, ZNF354C, GLIS3, ZNF25, EGR2, KLF5, ZNF396, ZNF559, ZNF491, ZNF43, ZFP82, ZNF345, ZNF585A, ZNF546, ZNF615, ZNF583, ZNF71, ZNF549, OVOL2, ZNF648, ZNF774, ZNF267, ZNF599, ZFP30, ZNF701, ZNF547, ZNF594, ZNF682, ZNF419, ZNF117, ZNF432, ZNF90, KLF6, SNAI2, ZNF285, ZNF667, ZNF471, ZNF793, BCL6B, IKZF4, CTCFL, YY2, ZNF331, KLF17, ZNF502, FEZF2, ZEB1, ZNF33B, ZIC2, ZNF668, ZFP3, ZNF333, ZNF566, ZNF420, ZNF585B, ZNF175, ZNF614, ZNF611, ZNF160, ZNF211, ZNF700, ZNF101, ZNF85, ZNF181, ZNF528, ZNF320, ZNF141, ZSCAN20, ZNF564, ZNF100, ZNF418, HIVEP1, REPIN1, ZNF251, ZNF534, ZNF749, ZNF705G, EGR1, BCL11A, ZNF510, ZNF846, ZBTB7A, ZNF192, ZNF624, ZNF439, ZNF675, SP3, ZNF189, ZNF521, ZBTB48, ZBTB11, KLF15, PRDM5, SP8, ZNF107, ZNF572, ZNF438, ZSCAN2, ZNF19, ZNF232, ZNF556, ZNF57, ZNF562, ZNF490, ZNF791, ZNF14, ZNF567, ZNF570, ZNF571, ZNF540, ZNF781, ZNF221, ZNF223, ZNF460, ZNF584, ZNF133, GZF1, ZBTB41, ZNF670, ZNF705A, SALL2, ZKSCAN2, ZNF319, ZNF93, ZNF430, ZNF222, ZNF473, ZNF468, ZNF543, RREB1, ZNF623, ZFP1, ZNF239, ZNF195, ZFP90, ZNF440, ZNF829, PRDM13, ZNF3, ZNF12, ZNF274, FLJ38451, MGC33414, ZNF225, ZNF736, ZNF683, ZNF792, ZNF808, ZBTB16, ZKSCAN4, ZNF323, ZNF485, ZNF555, ZNF426, ZNF45, ZNF484, ZNF30, ZNF730, ZNF250, RBAK, ZNF134, ZSCAN10, GLI1, SNAI1, ZNF157, ZNF254, ZNF384, ZNF691, ZNF672, ZNF514, ZNF619, ZNF148, ZBTB49, ZNF354B, ZNF184, ZKSCAN5, ZNF655, ZNF398, ZNF16, ZNF367, ZNF248, ZNF408, ZNF10, KLF12, ZNF205, ZNF200, ZNF263, ZNF75A, ZNF286A, SP6, ZNF24, ZNF558, ZNF317, ZNF565, ZNF146, ZNF574, ZNF227, ZNF610, ZSCAN5A, ZFP28, ZNF304, ZNF416, ZNF530, ZNF551, ZNF671, ZNF552, ZNF417, ZNF329, ZNF132, SALL4, ZNF70, GFI1, ZNF124, ZNF214, ZNF84, ZNF121, ZNF136, ZNF431, ZFP14, ZNF224, ZNF233, ZNF582, ZNF311, ZNF713, ZNF92, ZKSCAN1, ZNF782, ZNF501, ZNF273, ZNF169, ZNF768, ZNF358, ZNF154, ZNF74, ZBTB38, ZNF786, Q6ZMS4, ZBTB34, ZNF407, ENSG00000177835, ZNF765, ZNF20, ZNF813, ZNF724P, ZNF284, ZNF234, ZNF302, ZNF695, ZNF852, ZNF226, SP4, ZNF500, ZBTB20, ZNF498, FIZ1, ZNF669, ZNF708, ZNF235, ZNF347, ZNF773, GLI2, ZNF350, ZFX, ZNF41, ZNF630, ZBTB17, ZNF684, ZBTB7B, ZNF513, ZNF621, ZNF366, ZNF354A, ZNF165, ZNF282, ZNF746, ZNF467, ZFP41, GLI4, SCRT1, ZNF517, ZNF7, ZNF79, ZNF32, ZNF215, ZNF434, ZNF785, ZNF689, ZNF23, ZNF77, ZNF561, KLF1, ZNF581, ZSCAN4, ZSCAN22, ZNF337, ZNF335, HIC2, ZNF260, ZNF264, ZNF324B, ZNF680, ZNF658, ZNF483, ZNF35, ZNF596, ZNF143, ZSCAN30, ZNF557, ZNF699, ZNF627, ZNF253, ZNF527, ZNF787, ZNF2, ZFAT, ZNF283, ZNF697, ZNF812, ZNF729, ZNF81, ZNF404, ZNF805, ZFP62, ZNF766, ZNF778, ZNF544, ZNF66P, ZNF197, IKZF2, ZNF383, ZNF554, BCL11B, KLF10, IKZF1, KLF4, KLF8, PRDM1, ZNF449, PRDM16, E4F1, ZNF362, GLIS1, ZNF445, ZNF660, MYNN, ZKSCAN3, ZBTB22, ZNF696, ZBTB6, GFI1B, ZNF22, ZNF664, KLF13, ZNF770, ZNF710, ZNF287, ZFP161, ZNF653, KLF2, ZNF155, ZNF114, ZNF613, ZNF587, ZNF497, ZNF324, SCRT2, ZNF341, ZBTB8A, ZNF605, ZBTB1, VEZF1, ZNF230, ZNF772, ZNF620, ZBTB24, ZNF789, ZNF550, HIC1, ZNF17, ZNF586, ZNF142, ZNF589, ZXDC, ZNF775, NM, Q7Z7K7, ZNF257, ZNF788, ZNF763, ZNF34, ZNF879, ZNF268, ZNF548, OSR2, ZNF727, ZNF433, ZNF844, ZNF44, ZNF525, ZNF705B, ZNF850, ZNF300, OSR1, MZF1, ZSCAN12, YY1, REST, GLI3, ZNF507, ZBTB33, ZNF75D, ZBTB40, HIVEP3, EGR4, ZSCAN21, KLF14, ZBTB3, OVOL1, PRDM4, ZNF140, ZNF219, ZNF410, ZNF597, ZNF48, KLF16, ZNF526, ZNF575, ZNF784, ZBTB45, ZBTB46, PRDM15, ZNF642, ZFP91, ZNF26, ZNF18, ZNF652, ZNF397, ZBTB7C, ZNF833P, SP5, ZNF518B, FEZF1, IKZF5, MAZ, ZNF506, ZFP57, ZNF834, ZBTB44, ZSCAN23, ZBTB47, ZNF629, SP7, GTF3A, ZNF628, ZBTB8B, ZNF394, GLIS2, EGR3, ZNF764, ZBTB39, HINFP, KLF11, ZFP92, PATZ1, ZBTB37, ZNF281, KLF7, ZNF193, PLAGL1, ZBTB5, KLF9, ZBTB26, ZBTB43, ZNF641, ZNF213, ZNF174, ZNF296, ZNF524, ZNF444, ZNF343, ZNF692, ZNF69, ZIC5, ZSCAN29, HIVEP2, ZNF777, ZNF837, ZNF516, ZNF278, ZNF707, PRDM6, ZSCAN5C, ZNF891, ZNF740, SP9, ZNF252P, ZNF771, ENSG00000188474, ZFPM1, PLAGL2, ZNF639, ZNF322A, ZSCAN16, ZNF212, PRDM12, ZNF202, ZNF688, SNAI3, ZNF658B, SP2, ZBTB10, ZNF674, AEBP2, ZNF720, ZSCAN5D, ZNF747, SP1, ZNF217, ZBTB2, ZNF646, ZBTB32, ZNF579, ZFY, ZNF826P, ZNF705D, Q7M4M3, ZNF487P, ZBTB42, OVOL3 |

The significant domains in whole genome are listed by the Pfam domain name, the number of domains, the mutation enrichment expressed as the ratio of the observed number of domain mutations to the expected number of mutation, the Bonferroni corrected p-value and the gene names. The list sorted by enrichment score followed by the number of domains.

*Indicates that the initial *p*-value was calculated using Fisher’s exact test.
