# Supplementary material for: Identification and analysis of mutational hotspots in oncogenes and tumour suppressors
Source: Oncotarget. 2017 Feb 19;8(13):21290–304. doi: 10.18632/oncotarget.15514 (PMC5400584; doi:10.18632/oncotarget.15514)
Supplement: Supplementary file 5 [file oncotarget-08-21290-s005.docx]

**Supplementary Table 9:** Domains enriched in truncation mutations in the whole genome.

| **Domains** | **No of domains** | **Enrichment score** | ***p*-value** | **Genes** |
| --- | --- | --- | --- | --- |
| APC_crr | 2 | 27.92 | 1.80468E-80* | APC, APC2 |
| Gal_Lectin | 3 | 21.59 | 1.11855E-75* | ENSG00000166473, LPHN2, PKD1L2 |
| VHL | 1 | 18.19 | 2.58048E-87* | VHL |
| NHR2 | 1 | 16.69 | 1.41522E-40 | RUNX1T1 |
| DUF3454 | 3 | 15.71 | 1.42647E-48* | NOTCH1, NOTCH2, NOTCH3 |
| SAMP | 1 | 12.01 | 3.52654E-18 | APC |
| PLDc | 1 | 11.83 | 9.76661E-07 | PLD2 |
| Tower | 1 | 11.66 | 5.75749E-11 | BRCA2 |
| RNA_pol_Rpb1_R | 2 | 11.52 | 3.00015E-68 | POLR2A, ZNF768 |
| YHS | 3 | 11.34 | 1.07385E-35 | AKD1, ENSG00000188423, FLJ42177 |
| TGF_beta_GS | 1 | 11.34 | 9.48637E-15 | ACVR1B |
| Xin | 1 | 10.47 | 7.74718E-21 | XIRP2 |
| Hormone_2 | 2 | 10.13 | 9.02905E-11 | GCG, VIP |
| GoLoco | 3 | 9.82 | 1.19469E-22 | GPSM2, GPSM3, RGS12 |
| Involucrin | 1 | 9.43 | 1.74731E-36 | IVL |
| APC_15aa | 1 | 9.28 | 9.46757E-13 | APC |
| RB_A | 3 | 8.55 | 1.06058E-60* | RB1, RBL1, RBL2 |
| P53_TAD | 1 | 8.50 | 9.92881E-10 | TP53 |
| SAP | 7 | 8.10 | 1.44823E-32 | CCAR1, DEK, MAMSTR, MKL1, MKL2, MYOCD, RAD18 |
| Ig_Tie2_1 | 1 | 8.00 | 1.36183E-11 | TEK |
| FATC | 3 | 7.91 | 2.18346E-11 | ATM, ENSG00000121031, PRKDC |
| hEGF | 15 | 7.63 | 1.82881E-27 | CRB2, DLL3, JAG2, MEGF10, MEGF11, NOTCH1, NOTCH3, NOTCH4, PEAR1, Q8N2E2, SLIT3, TIE1, TNXB, VWDE, WIF1 |
| Tubulin-binding | 3 | 7.44 | 5.02649E-24 | MAP2, MAP4, MAPT |
| UIM | 3 | 7.39 | 0.000514672 | ATXN3, STAM2, USP25 |
| PTEN_C2 | 9 | 7.37 | 4.879E-113* | DNAJC6, ENSG00000197585, PTEN, Q5JV89, TENC1, TNS1, TNS3, TPTE, TPTE2 |
| Titin_Z | 1 | 7.18 | 6.87716E-30 | TTN |
| F-box-like | 7 | 6.93 | 4.23887E-34 | GATA2, GATA3, GATA4, GATA5, MTA2, MTA3, TRPS1 |
| GATA | 11 | 6.93 | 6.62883E-60 | ECT2L, FBXL3, FBXO15, FBXO16, FBXO18, FBXO31, FBXO39, FBXO48, FBXO6, FBXW12, FBXW7 |
| LRR_7 | 4 | 6.92 | 0.000123352 | LRRC58, LRRIQ4, TLR10, TLR2 |
| RPEL | 4 | 6.80 | 8.41829E-18 | MKL2, PHACTR1, PHACTR3, PHACTR4 |
| Proteasome_A_N | 1 | 6.80 | 0.002359918 | RAD50 |
| Rad50_zn_hook | 2 | 6.80 | 0.032252244 | PSMA1, PSMA8 |
| VGCC_beta4Aa_N | 3 | 6.68 | 9.08327E-10 | CACNB1, CACNB2, CACNB4 |
| BRCA2 | 1 | 6.58 | 9.01594E-14 | BRCA2 |
| SH3_3 | 1 | 6.58 | 0.000344911 | MYCBP2 |
| zf-CCHC | 4 | 6.18 | 0.000120383 | ZCCHC11, SF1, ZCCHC6, ZCCHC5 |
| zf-C2H2 | 108 | 6.16 | 2.9324E-109* | BCL11B, CTCF, EGR1, FEZF1, GLI2, HKR1, LOC152485, MAZ, MECOM, MZF1, OSR2, OVOL1, OVOL2, PEG3, PLAG1, PRDM16, PRDM2, PRDM8, PRDM9, RBAK, REPIN1, SNAI2, ZBTB1, ZBTB11, ZBTB16, ZBTB20, ZBTB38, ZBTB39, ZBTB41, ZFHX4, ZFP57, ZFP90, ZFX, ZIC4, ZIC5, ZIM2, ZKSCAN4, ZNF10, ZNF131, ZNF140, ZNF142, ZNF16, ZNF197, ZNF202, ZNF232, ZNF256, ZNF276, ZNF285, ZNF286A, ZNF292, ZNF304, ZNF320, ZNF329, ZNF333, ZNF334, ZNF33A, ZNF34, ZNF343, ZNF366, ZNF383, ZNF384, ZNF391, ZNF394, ZNF398, ZNF41, ZNF420, ZNF423, ZNF426, ZNF442, ZNF443, ZNF445, ZNF473, ZNF498, ZNF507, ZNF516, ZNF530, ZNF532, ZNF536, ZNF567, ZNF57, ZNF573, ZNF579, ZNF580, ZNF581, ZNF582, ZNF599, ZNF610, ZNF629, ZNF639, ZNF646, ZNF652, ZNF667, ZNF680, ZNF681, ZNF717, ZNF721, ZNF74, ZNF768, ZNF772, ZNF780A, ZNF789, ZNF799, ZNF814, ZNF827, ZNF837, ZNF91, ZSCAN18, ZXDB |
| ANATO | 4 | 6.15 | 2.25796E-11 | FBLN1, C5, C3, FBLN2 |
| CDI | 3 | 6.11 | 1.54961E-25 | CDKN1A, CDKN1B, CDKN1C |
| zf-H2C2_5 | 8 | 6.06 | 2.27069E-23 | LOC152485, ZFP64, ZFX, ZFY, ZNF219, ZNF462, ZNF507, ZNF827 |
| zf-CCCH | 11 | 6.05 | 3.59829E-39 | DHX57, HELZ, MBNL1, MKRN1, PARP12, PPP1R10, RC3H2, ZC3H3, ZFP36, ZFP36L1, ZFP36L2, SF1, ZCCHC11, ZCCHC5, ZCCHC6 |
| IQ | 26 | 6.00 | 9.55982E-61 | ASPM, CAMTA1, CAMTA2, GAP43, INVS, IQCE, IQCF1, IQCF2, IQCF3, IQCF5, IQCF6, IQGAP1, IQGAP2, IQGAP3, KIAA1683, LRRIQ1, MYO1A, MYO1B, MYO5C, MYO7B, MYO9A, OBSCN, PPEF1, SPA17, SPATA17, UBE3B |
| LEM | 4 | 5.98 | 6.27732E-10 | ANKLE2, FLJ39369, LEMD1, TMPO |
| GHMP_kinases_N | 2 | 5.96 | 5.90832E-09 | GALK2, MVK |
| ITAM | 5 | 5.92 | 3.70868E-05 | CD247, CD3D, CD79A, CD79B, FCER1G |
| DDA1 | 1 | 5.86 | 0.026423494 | DDA1 |
| Rb_C | 2 | 5.83 | 2.32975E-27 | RB1, RBL1 |
| SAE2 | 1 | 5.77 | 0.034127395 | RBBP8 |
| PHD_2 | 3 | 5.67 | 0.000100735 | BRPF3, MLLT10, PHF15 |
| BAH | 7 | 5.55 | 6.66944E-47* | BAHD1, DNMT1, MTA1, MTA2, PBRM1, RERE, TNRC18 |
| PDEase_I_N | 2 | 5.52 | 2.71523E-05 | PDE1B, PDE1C |
| OSR1_C | 4 | 5.44 | 5.72776E-06 | OXSR1, STK39, WNK2, WNK4 |
| z-alpha | 2 | 5.43 | 2.25604E-08 | ADAR, ZBP1 |
| Cu2_monooxygen | 1 | 5.42 | 4.2293E-05 | PAM |
| fn1 | 3 | 5.31 | 2.55539E-14 | F12, FN1, HGFAC |
| Casein | 1 | 5.30 | 0.017644518 | CSN2 |
| Ant_C | 2 | 5.14 | 1.42545E-07 | ANTXR1, ANTXR2 |
| OAR | 5 | 5.12 | 0.005309794 | ALX1, OTP, PITX2, PITX3, VSX2 |
| LRRNT | 8 | 5.11 | 6.63757E-15 | ASPN, DCN, LINGO2, LRRTM3, SLIT1, SLIT3, TPBG, VASN |
| RB_B | 2 | 5.05 | 5.44795E-24 | RB1, RBL1 |
| PACT_coil_coil | 1 | 5.05 | 3.10635E-10 | AKAP9 |
| zf-C2H2_jaz | 7 | 5.04 | 5.17251E-08 | CIZ1, GPATCH8, NUFIP1, PRDM10, PRDM5, ZNF593, ZNF804A |
| zf-C2H2_4 | 45 | 5.02 | 8.07727E-61 | ATMIN, CTCF, IKZF3, INSM2, LOC152485, LOC168850, PLAGL1, PLAGL2, PRDM10, PRDM14, REST, RREB1, SALL2, SALL3, TRERF1, ZBTB1, ZBTB11, ZBTB20, ZBTB40, ZFP42, ZIC4, ZNF131, ZNF142, ZNF219, ZNF276, ZNF296, ZNF335, ZNF423, ZNF451, ZNF516, ZNF521, ZNF526, ZNF57, ZNF574, ZNF579, ZNF580, ZNF618, ZNF641, ZNF644, ZNF646, ZNF670, ZNF672, ZNF687, ZNF800, ZNF827 |
| zf-MYND | 7 | 4.95 | 5.63901E-12 | DEAF1, RUNX1T1, SMYD2, SMYD3, ZMYND10, ZMYND12, ZMYND8 |
| Integrin_b_cyt | 2 | 4.95 | 0.001864439 | ITGB2, ITGB3 |
| zf-CXXC | 7 | 4.89 | 2.41662E-19 | CXXC1, CXXC5, FBXL19, KDM2A, MBD1, MLL, MLL4 |
| FYRN | 5 | 4.89 | 3.60021E-15 | KMT2D, MLL2, MLL3, MLL4, TBRG1 |
| Lep_receptor_Ig | 2 | 4.86 | 4.37082E-06 | CSF3R, LEPR |
| zf-C5HC2 | 1 | 4.81 | 0.01596873 | KDM5C |
| FF | 7 | 4.79 | 2.87013E-39 | ARHGAP35, ARHGAP5, GRLF1, PRPF40A, PRPF40B, TCERG1, TCERG1L |
| HS1_rep | 2 | 4.77 | 0.003755023 | AMFR, TAB3 |
| CUE | 2 | 4.77 | 3.59238E-05 | CTTN, HCLS1 |
| DUF3452 | 3 | 4.72 | 1.09335E-26 | RB1, RBL1, RBL2 |
| DED | 4 | 4.70 | 6.29918E-22 | CASP10, CASP8, CFLAR, FADD |
| DSL | 2 | 4.69 | 0.005231535 | DLL4, JAG1 |
| Casc1 | 1 | 4.67 | 6.79846E-07 | CASC1 |
| PI31_Prot_N | 1 | 4.57 | 0.001971029 | FBXO7 |
| ING | 3 | 4.55 | 3.9769E-12 | ING1, ING2, ING5 |
| QLQ | 2 | 4.54 | 2.3524E-08 | MUC4, ISM2 |
| AMOP | 2 | 4.54 | 0.002295781 | SMARCA2, SMARCA4 |
| zf-met | 15 | 4.48 | 1.3231E-16 | BNC2, LOC168850, PRDM10, PRDM5, TUT1, ZFHX3, ZFHX4, ZFR, ZFR2, ZMAT1, ZMAT4, ZNF131, ZNF346, ZNF385D, ZNF800 |
| Thiol-ester_cl | 5 | 4.43 | 0.000229311 | A2ML1, C3, CD109, CPAMD8, PZP |
| zf-RanBP | 10 | 4.42 | 1.69008E-11 | EWSR1, FUS, MDM2, NEIL3, NUP153, RANBP2, RBM10, SHARPIN, ZRANB1, ZRANB2 |
| Nebulin | 4 | 4.36 | 4.03982E-45 | LASP1, NEB, NEBL, NRAP |
| MORN | 10 | 4.25 | 3.09253E-16 | ALS2, ALS2CL, JPH1, JPH2, JPH3, MORN1, MORN2, MORN3, RSPH1, RSPH10B |
| zf-H2C2_2 | 467 | 4.21 | 0* | BCL11A, BCL6, CTCF, CTCFL, EGR1, EGR2, EGR3, ENSG00000197608, ENSG00000198153, ENSG00000221895, ENSG00000257591, FEZF1, FIZ1, FLJ38451, GLI1, GLI2, GLI4, GLIS1, GLIS2, GLIS3, GZF1, HINFP, HIVEP2, HKR1, IKZF2, IKZF3, IKZF5, KLF1, KLF12, KLF14, KLF15, KLF17, KLF3, KLF6, KLF7, KLF9, MAZ, MTF1, MYNN, MZF1, OSR1, OSR2, PATZ1, PLAG1, PRDM12, PRDM5, PRDM9, Q6ZMS4, Q7Z7K7, RBAK, REPIN1, REST, SALL1, SALL2, SALL3, SCRT2, SNAI2, SP1, SP3, SP4, SP7, WT1, YY2, ZBTB10, ZBTB11, ZBTB16, ZBTB17, ZBTB20, ZBTB22, ZBTB24, ZBTB3, ZBTB32, ZBTB33, ZBTB37, ZBTB38, ZBTB39, ZBTB41, ZBTB45, ZBTB48, ZBTB49, ZBTB6, ZBTB7A, ZBTB7B, ZBTB8B, ZEB1, ZEB2, ZFAT, ZFP112, ZFP14, ZFP161, ZFP2, ZFP28, ZFP3, ZFP30, ZFP37, ZFP41, ZFP42, ZFP62, ZFP82, ZFP90, ZFP92, ZFPM1, ZFX, ZFY, ZIC1, ZIC4, ZIM3, ZKSCAN1, ZKSCAN2, ZKSCAN3, ZKSCAN4, ZKSCAN5, ZNF10, ZNF100, ZNF101, ZNF107, ZNF114, ZNF117, ZNF12, ZNF121, ZNF124, ZNF132, ZNF133, ZNF134, ZNF135, ZNF136, ZNF14, ZNF140, ZNF142, ZNF146, ZNF148, ZNF154, ZNF155, ZNF157, ZNF16, ZNF160, ZNF165, ZNF167, ZNF169, ZNF174, ZNF175, ZNF18, ZNF180, ZNF181, ZNF182, ZNF184, ZNF189, ZNF19, ZNF192, ZNF193, ZNF197, ZNF20, ZNF202, ZNF208, ZNF211, ZNF215, ZNF217, ZNF22, ZNF221, ZNF222, ZNF223, ZNF224, ZNF225, ZNF226, ZNF229, ZNF23, ZNF230, ZNF233, ZNF234, ZNF235, ZNF236, ZNF238, ZNF239, ZNF25, ZNF250, ZNF251, ZNF254, ZNF256, ZNF26, ZNF260, ZNF264, ZNF267, ZNF268, ZNF273, ZNF275, ZNF278, ZNF28, ZNF281, ZNF282, ZNF283, ZNF284, ZNF285, ZNF286A, ZNF286B, ZNF287, ZNF296, ZNF3, ZNF30, ZNF304, ZNF311, ZNF317, ZNF319, ZNF320, ZNF322A, ZNF323, ZNF331, ZNF333, ZNF334, ZNF337, ZNF33A, ZNF33B, ZNF34, ZNF341, ZNF345, ZNF347, ZNF350, ZNF354A, ZNF354B, ZNF354C, ZNF358, ZNF367, ZNF37A, ZNF382, ZNF383, ZNF391, ZNF394, ZNF398, ZNF404, ZNF407, ZNF41, ZNF415, ZNF416, ZNF417, ZNF418, ZNF419, ZNF420, ZNF423, ZNF425, ZNF426, ZNF429, ZNF43, ZNF430, ZNF431, ZNF432, ZNF433, ZNF434, ZNF436, ZNF438, ZNF439, ZNF44, ZNF440, ZNF441, ZNF442, ZNF443, ZNF449, ZNF45, ZNF454, ZNF460, ZNF468, ZNF470, ZNF471, ZNF473, ZNF479, ZNF48, ZNF480, ZNF483, ZNF484, ZNF485, ZNF486, ZNF490, ZNF491, ZNF492, ZNF493, ZNF497, ZNF498, ZNF500, ZNF501, ZNF502, ZNF506, ZNF510, ZNF513, ZNF514, ZNF519, ZNF525, ZNF527, ZNF528, ZNF530, ZNF534, ZNF536, ZNF540, ZNF543, ZNF544, ZNF546, ZNF547, ZNF548, ZNF549, ZNF550, ZNF551, ZNF552, ZNF554, ZNF555, ZNF557, ZNF558, ZNF560, ZNF563, ZNF564, ZNF565, ZNF566, ZNF567, ZNF568, ZNF569, ZNF57, ZNF570, ZNF571, ZNF572, ZNF573, ZNF577, ZNF581, ZNF582, ZNF583, ZNF584, ZNF585A, ZNF585B, ZNF586, ZNF587, ZNF589, ZNF594, ZNF596, ZNF597, ZNF599, ZNF600, ZNF606, ZNF607, ZNF611, ZNF613, ZNF614, ZNF615, ZNF616, ZNF619, ZNF620, ZNF621, ZNF623, ZNF624, ZNF625, ZNF626, ZNF627, ZNF630, ZNF641, ZNF649, ZNF652, ZNF653, ZNF655, ZNF658, ZNF660, ZNF662, ZNF664, ZNF665, ZNF667, ZNF668, ZNF669, ZNF66P, ZNF670, ZNF671, ZNF675, ZNF676, ZNF677, ZNF678, ZNF680, ZNF681, ZNF682, ZNF689, ZNF691, ZNF695, ZNF696, ZNF697, ZNF699, ZNF7, ZNF700, ZNF701, ZNF708, ZNF709, ZNF71, ZNF711, ZNF714, ZNF716, ZNF717, ZNF720, ZNF721, ZNF724P, ZNF727, ZNF729, ZNF730, ZNF732, ZNF736, ZNF737, ZNF749, ZNF75A, ZNF75D, ZNF763, ZNF764, ZNF765, ZNF766, ZNF768, ZNF77, ZNF772, ZNF773, ZNF774, ZNF776, ZNF778, ZNF780A, ZNF780B, ZNF781, ZNF782, ZNF784, ZNF786, ZNF788, ZNF789, ZNF79, ZNF790, ZNF791, ZNF792, ZNF793, ZNF799, ZNF80, ZNF805, ZNF808, ZNF81, ZNF812, ZNF813, ZNF814, ZNF816, ZNF823, ZNF826P, ZNF829, ZNF83, ZNF831, ZNF833P, ZNF835, ZNF836, ZNF837, ZNF84, ZNF841, ZNF844, ZNF845, ZNF846, ZNF85, ZNF850, ZNF852, ZNF878, ZNF879, ZNF880, ZNF90, ZNF91, ZNF92, ZNF93, ZNF98, ZNF99, ZSCAN12, ZSCAN16, ZSCAN20, ZSCAN21, ZSCAN22, ZSCAN29, ZSCAN5A, ZSCAN5B, ZSCAN5C, ZSCAN5D |
| Bromodomain | 29 | 4.16 | 2.32596E-77 | ATAD2, ATAD2B, BAZ1B, BAZ2A, BAZ2B, BRD1, BRD2, BRD3, BRD4, BRD7, BRD8, BRD9, BRDT, BRPF3, BRWD1, BRWD3, CECR2, CREBBP, ENSG00000184100, KAT2B, PBRM1, PHIP, SMARCA2, SMARCA4, SP140, TAF1, TAF1L, TRIM33, ZMYND8 |
| zf-C2H2_6 | 26 | 4.10 | 2.51871E-19* | LOC168850, PLAGL2, SNAI2, ZFP64, ZFPM1, ZFPM2, ZNF175, ZNF296, ZNF304, ZNF43, ZNF445, ZNF521, ZNF526, ZNF556, ZNF560, ZNF574, ZNF600, ZNF606, ZNF607, ZNF646, ZNF675, ZNF691, ZNF770, ZNF787, ZNF79, ZNF800 |
| TFIID-18kDa | 2 | 3.98 | 0.008599724 | SUPT3H, TAF13 |
| Longin | 2 | 3.98 | 0.028313072 | SEC22A, VAMP7 |
| DUF2039 | 1 | 3.96 | 0.009642446 | C9orf85 |
| LRR_1 | 26 | 3.94 | 4.94604E-17 | CD14, CD180, LGR5, LGR6, LRR1, LRRC10, LRRC31, LRRC4, LRRC4C, LRRC52, LRRC57, LRRC7, LRRCC1, LRRK2, LRRTM1, LRRTM3, NXF1, PHLPP2, PODNL1, SHOC2, SLIT2, SLITRK3, TLR3, TLR7, TLR8, TLR9 |
| WW | 21 | 3.92 | 5.75137E-13 | CMIP, FBXL13, FBXL14, FBXL2, FBXL5, FBXL7, FMOD, LRRC16A, NLRC5, NLRP1, NLRP12, NLRP13, NLRP2, NLRP4, NLRP5, NLRP7, NLRP8, NOD1, NOD2, RLTPR, RNH1 |
| LRR_6 | 22 | 3.92 | 1.15333E-21 | APBB3, BAG3, DMD, DRP2, ENSG00000189290, FNBP4, GAS7, HECW2, ITCH, MAGI2, NEDD4, NEDD4L, PIN1, PRPF40B, SAV1, SETD2, SMURF2, WAC, WBP4, WWP1, WWP2, YAP1 |
| SPAN-X | 10 | 3.85 | 3.77242E-26 | SPANXA1, SPANXA2, SPANXB1, SPANXB2, SPANXD, SPANXN1, SPANXN2, SPANXN3, SPANXN4, SPANXN5 |
| EF-hand_6 | 7 | 3.85 | 0.001743685 | CALB1, CALB2, CAPN3, CAPNS1, EFCAB11, EFCAB2, NIN |
| DND1_DSRM | 2 | 3.82 | 0.018629092 | DND1, SON |
| HARE-HTH | 3 | 3.81 | 1.10336E-06 | ASXL1, ASXL2, ASXL3 |
| WH2 | 7 | 3.78 | 1.25918E-05 | COBL, LMOD1, LOC392529, WAS, WASF3, WASL, WIPF2 |
| EGF | 38 | 3.74 | 1.2228E-69 | ACAN, AGC1, BCAN, CRB1, CRB2, DLK1, DLL1, DLL3, DLL4, DNER, EDIL3, ENSG00000165124, EYS, F12, FAT, FAT1, HABP2, HEG1, HGFAC, JAG1, JAG2, MEP1A, MFGE8, MMRN1, NOTCH1, NOTCH2, NOTCH2NL, NOTCH3, NOTCH4, PROS1, Q5T669, SELL, SLIT1, SLIT2, SLIT3, SNED1, SVEP1, VCAN |
| Arm | 13 | 3.65 | 2.57231E-22 | APC, ARMC1, CTNND1, CTNND2, ENSG00000185467, KPNA2, KPNA3, KPNA4, KPNA5, KPNA7, KPNB1, PKP4, SPAG6 |
| TPR_1 | 25 | 3.60 | 1.50025E-22 | CDC16, CDC23, CDC27, CNOT10, CTR9, DNAJC7, GPSM2, KDM6A, KLC1, NASP, NCF2, OGT, PEX5L, PPID, RGPD3, STIP1, TOMM34, TTC1, TTC17, TTC3, TTC37, TTC3P1, TTC6, TTC9, UNC45B |
| Ank | 27 | 3.58 | 7.5828E-37 | ABTB2, ACAP1, ANK1, ANK2, ANKRD12, ANKRD13A, ANKRD28, ANKRD7, ANKS1A, ASB4, BCL3, CDKN2A, CDKN2C, KANK1, NFKBIA, NFKBIE, NOTCH1, PPP1R12A, PPP1R12B, PSMD10, RNASEL, TNKS, TP53BP2, TRPA1, TRPC3, TRPC4, TRPC5 |
| TPR_2 | 9 | 3.58 | 1.3384E-06 | AIPL1, CDC27, DNAJC7, IFIT5, NAA15, TTC12, TTC16, TTC23, TTC29 |
| zf-FCS | 5 | 3.50 | 1.88027E-05 | GRIA2, GRIA4, GRIK2, GRIK3, GRIK4 |
| Lig_chan-Glu_bd | 7 | 3.50 | 1.82898E-13 | PHC1, PHC1B, ZMYM1, ZMYM3, ZMYM4, ZMYM5, ZMYM6 |
| Kelch_5 | 6 | 3.45 | 0.00288517 | KLHDC9, KARCA1, ATRNL1, KLHDC1, HCFC2, LZTR1 |
| Defensin_beta_2 | 15 | 3.41 | 7.42934E-06 | DEFB106A, DEFB106B, DEFB110, DEFB111, DEFB112, DEFB113, DEFB116, DEFB118, DEFB123, DEFB124, DEFB127, DEFB129, DEFB131, DEFB132, DEFB133 |
| EF-hand_1 | 12 | 3.35 | 0.000415143 | CABP1, CABP5, CALM1, CALM2, CALM3, CALML3, CALML5, CHP2, GUCA1C, HPCAL4, NCS1, VSNL1 |
| DUF3518 | 2 | 3.35 | 2.50878E-24 | ARID1A, ARID1B |
| SWIRM | 3 | 3.35 | 0.033377597 | SMARCC1, SMARCC2, TADA2A |
| FRG1 | 3 | 3.34 | 1.13187E-17 | C20orf80, FRG1, FRG1B |
| TAN | 1 | 3.32 | 0.002879574 | ATM |
| Tis11B_N | 2 | 3.29 | 4.44851E-06 | ZFP36L1, ZFP36L2 |
| WD40 | 156 | 3.25 | 7.0673E-106* | AAAS, AAMP, APAF1, ARPC1A, ARPC1B, ATG16L1, BRWD1, BRWD3, BTRC, C10orf64, CDC20, CDC20B, CDC40, CDRT1, CIAO1, COPA, COPB2, CORO1A, CORO2A, CORO6, CORO7, CSTF1, DCAF12L1, DCAF12L2, DCAF6, DCAF8L1, DCAF8L2, DMWD, DMXL1, DMXL2, DNAI1, DNAI2, DTL, EDC4, ELP2, EML1, EML2, EML5, EML6, ENSG00000183851, ENSG00000241322, ENSG00000251537, ERCC8, FBXW10, FBXW11, FBXW2, FBXW4, FBXW5, FBXW7, FBXW8, FZR1, GEMIN5, GNB1L, GNB2, GNB2L1, GNB3, GNB4, HERC1, HIRA, IFT80, KATNB1, KIF21A, KIF21B, LLGL2, LOC340578, MAPKBP1, MED16, MLST8, NBEA, NLE1, NSMAF, NWD1, PAAF1, PAFAH1B1, PAK1IP1, PEX7, PHIP, PIK3R4, PLAA, PLRG1, POC1B, PPWD1, PRPF19, PRPF4, PWP1, PWP2, RAE1, RBBP4, RBBP7, RFWD2, RRP9, SEC13, SEC31A, SEH1L, SMU1, SPAG16, STRAP, STRN, STRN3, STRN4, TAF5, TAF5L, TBL1X, TBL1XR1, TBL1Y, TBL3, TEP1, TLE1, TLE2, TLE3, TLE4, TRAF7, TSSC1, WDFY1, WDFY2, WDFY3, WDFY4, WDHD1, WDR1, WDR12, WDR16, WDR17, WDR24, WDR26, WDR27, WDR3, WDR31, WDR33, WDR38, WDR44, WDR45, WDR47, WDR48, WDR49, WDR5, WDR53, WDR59, WDR5B, WDR6, WDR62, WDR63, WDR64, WDR66, WDR69, WDR7, WDR70, WDR72, WDR75, WDR76, WDR82, WDR88, WDR89, WDSUB1, WDTC1, WSB1, ZFP106 |
| ASXH | 2 | 3.24 | 3.59177E-06 | ASXL2, ASXL3 |
| SOCS_box | 13 | 3.23 | 3.11116E-08 | ASB11, ASB14, ASB15, ASB18, ASB4, ASB5, ASB7, ASB8, CISH, NEURL2, SPSB1, TULP4, WSB1 |
| STAG | 3 | 3.23 | 3.45633E-07 | STAG1, STAG2, STAG3L4 |
| DUF3583 | 1 | 3.23 | 0.005676605 | PML |
| EphA2_TM | 7 | 3.18 | 3.1422E-07 | EPHA2, EPHA3, EPHA4, EPHA5, EPHA8, EPHB1, EPHB6 |
| ZZ | 9 | 3.14 | 2.47079E-05 | DTNA, MIB1, DTNB, EP300, DRP2, KCMF1, DYTN, SQSTM1, CREBBP |
| fn2 | 7 | 3.13 | 0.005452321 | ELSPBP1, F12, FN1, HGFAC, MMP2, PLA2R1, SEL1L |
| PHD | 38 | 3.11 | 3.54713E-39 | AIRE, BAZ1A, BAZ2B, CHD3, CXXC1, DIDO1, DPF1, DPF2, DPF3, ING1, ING2, ING4, KDM5A, KDM5B, KDM5C, KMT2D, MLL, MLL2, MLL3, MLL4, MYST3, NSD1, PHF1, PHF10, PHF14, PHF20, PHF20L1, PHF21B, PHF23, PHF3, PHF8, PYGO1, Q86U89, TAF3, TIF1, TRIM24, TRIM33, WHSC1 |
| DUF846 | 2 | 3.05 | 0.002225304 | FAM18B2, FAM18A |
| Sterol-sensing | 4 | 3.02 | 1.17598E-09 | HMGCR, NPC1, PTCH1, SCAP |
| zf-C2HC | 9 | 2.96 | 0.002376269 | EPB41, EPB41L1, EPB41L2, EPB41L4A, EPB41L4B, EPB41L5, FARP2, FRMD3, PTPN4 |
| FA | 4 | 2.96 | 0.039072127 | L3MBTL1, MYT1, MYT1L, ST18 |
| SHIPPO-rpt | 12 | 2.94 | 0.000391408 | ENSG00000166013, RFPL2, RFPL4B, SPRYD5, TRIM22, TRIM25, TRIM34, TRIM48, TRIM6, TRIM60, TRIM68, TRIM69 |
| zf-C3HC4_4 | 5 | 2.94 | 0.001525566 | C1orf201, C4orf37, ODF3, ODF3L1, ODF3L2 |
| Histone | 57 | 2.90 | 1.82745E-68 | CENPA, H2AFY, H2AFZ, H2AFZP2, H3F3A, H3F3B, HIST1H2AA, HIST1H2AB, HIST1H2AC, HIST1H2AD, HIST1H2AE, HIST1H2AG, HIST1H2AH, HIST1H2AI, HIST1H2AK, HIST1H2AL, HIST1H2AM, HIST1H2BC, HIST1H2BD, HIST1H2BE, HIST1H2BF, HIST1H2BG, HIST1H2BH, HIST1H2BI, HIST1H2BL, HIST1H2BM, HIST1H2BN, HIST1H2BO, HIST1H3A, HIST1H3B, HIST1H3C, HIST1H3D, HIST1H3E, HIST1H3F, HIST1H3G, HIST1H3H, HIST1H3I, HIST1H3J, HIST1H4A, HIST1H4B, HIST1H4C, HIST1H4D, HIST1H4E, HIST1H4F, HIST1H4H, HIST1H4I, HIST1H4J, HIST1H4K, HIST1H4L, HIST2H2AC, HIST2H2BE, HIST2H3A, HIST2H3C, HIST2H3D, HIST2H4A, HIST2H4B, HIST4H4 |
| EGF_CA | 1 | 2.88 | 1.3715E-08 | WT1 |
| WT1 | 45 | 2.88 | 7.96097E-56 | BMP1, CD97, COMP, CRELD1, CRELD2, CUBN, EFEMP1, EGF, EGFL4, EGFL6, EMR2, FAT4, FBLN5, FBLN7, FBN1, FBN2, FBN3, HEG1, HMCN1, LRP1, LRP1B, LRP2, LTBP1, LTBP2, LTBP3, LTBP4, MASP1, MEGF8, NELL1, NELL2, NID1, NOTCH1, NOTCH2, NOTCH2NL, NOTCH3, NPNT, PROS1, SCUBE1, SUSD1, THBS2, THBS3, THBS4, TPO, UMODL1, VWCE |
| Kazal_2 | 8 | 2.85 | 0.000142431 | EGFL4, KARCA1, KLHDC1, KLHDC10, KLHDC2, KLHDC9, MEGF8, MKLN1 |
| Kelch_4 | 19 | 2.85 | 1.87524E-13 | AGRN, FSTL1, FSTL5, IGFBP7, RECK, SLCO1B3, SLCO1B7, SLCO1C1, SLCO2A1, SLCO4C1, SLCO6A1, SMOC1, SMOC2, SPINK5, SPOCK3, TMEFF1, TMEFF2, WFIKKN1, WFIKKN2 |
| Sel1 | 12 | 2.83 | 0.001146688 | AKT2, AKT3, CIT, PKN2, PRKCA, PRKCB, PRKCH, PRKCI, RPS6KA1, RPS6KA6, RPS6KB2, STK38 |
| Pkinase_C | 7 | 2.83 | 0.00214075 | EEF2K, KIAA0141, KIAA0746, LRP2BP, SEL1L, SEL1L2, SEL1L3 |
| HMG_box | 19 | 2.79 | 3.89449E-08 | MID2, TIF1, TRIM22, TRIM24, TRIM26, TRIM27, TRIM28, TRIM34, TRIM37, TRIM41, TRIM42, TRIM49, TRIM49L2, TRIM50, TRIM54, TRIM55, TRIM59, TRIM6, TRIM71 |
| zf-B_box | 23 | 2.79 | 8.27838E-17 | CIC, HMG20A, HMGB2, HMGXB4, LEF1, PBRM1, SMARCE1, SOX11, SOX17, SOX21, SOX3, SOX5, SOX6, SOX9, SP100, SRY, TCF7, TCF7L2, TOX, TOX2, TOX3, TOX4, WDHD1 |
| TSP_1 | 11 | 2.78 | 0.040388973 | CBL, TNRC6C, USP13, UBQLN4, UBQLN1, TDRD3, UBL7, UBAC1, UBQLN2, LATS1, RAD23B |
| UBA | 49 | 2.78 | 3.02792E-77 | ADAMTS1, ADAMTS10, ADAMTS12, ADAMTS13, ADAMTS14, ADAMTS16, ADAMTS17, ADAMTS18, ADAMTS19, ADAMTS2, ADAMTS20, ADAMTS3, ADAMTS5, ADAMTS6, ADAMTS7, ADAMTS9, ADAMTSL1, ADAMTSL2, ADAMTSL3, ADAMTSL4, BAI1, BAI2, BAI3, C6, C7, C9, CFP, CTGF, HMCN1, ISM1, ISM2, PAPLN, RSPO1, RSPO3, SEMA5A, SEMA5B, SPON1, THBS1, THBS2, THSD1, THSD4, THSD7A, THSD7B, UNC5B, UNC5C, UNC5D, WISP1, WISP2, WISP3 |
| PapD-like | 3 | 2.75 | 4.35635E-06 | HYDIN, LOC652153, LOC652737 |
| WTX | 4 | 2.72 | 4.21636E-38 | AMER1, FAM123A, FAM123B, FAM123C |
| KRAB | 123 | 2.68 | 5.20924E-42 | ENSG00000128563, KRBA2, PMS2P3, PRDM9, ZFP14, ZFP28, ZFP92, ZNF124, ZNF133, ZNF135, ZNF154, ZNF155, ZNF169, ZNF181, ZNF19, ZNF192, ZNF202, ZNF208, ZNF212, ZNF215, ZNF221, ZNF225, ZNF229, ZNF233, ZNF234, ZNF25, ZNF253, ZNF254, ZNF264, ZNF267, ZNF268, ZNF273, ZNF274, ZNF28, ZNF283, ZNF284, ZNF285, ZNF287, ZNF333, ZNF334, ZNF337, ZNF33A, ZNF343, ZNF350, ZNF382, ZNF425, ZNF429, ZNF43, ZNF430, ZNF431, ZNF441, ZNF442, ZNF45, ZNF454, ZNF468, ZNF479, ZNF480, ZNF483, ZNF492, ZNF500, ZNF514, ZNF525, ZNF527, ZNF530, ZNF534, ZNF548, ZNF554, ZNF555, ZNF558, ZNF560, ZNF561, ZNF562, ZNF565, ZNF567, ZNF568, ZNF573, ZNF577, ZNF582, ZNF583, ZNF586, ZNF596, ZNF597, ZNF605, ZNF606, ZNF613, ZNF616, ZNF626, ZNF643, ZNF649, ZNF675, ZNF677, ZNF679, ZNF682, ZNF69, ZNF709, ZNF717, ZNF720, ZNF727, ZNF729, ZNF730, ZNF732, ZNF736, ZNF737, ZNF75A, ZNF766, ZNF772, ZNF778, ZNF790, ZNF791, ZNF793, ZNF799, ZNF81, ZNF814, ZNF829, ZNF836, ZNF846, ZNF85, ZNF878, ZNF880, ZNF91, ZNF92, ZNF93, ZNF98 |
| SH3_1 | 39 | 2.68 | 1.98489E-17 | ABL2, AHI1, ARHGAP4, ARHGEF26, BCAR1, CD2AP, CSK, DBNL, DNMBP, DOCK5, EPS8, EPS8L1, EPS8L2, FYN, GRAP2, GRAPL, HCK, ITSN2, LYN, MYO1E, NCF2, NCK1, NOSTRIN, NPHP1, PLCG2, RASA1, SGEF, SH3PXD2B, SLA, SORBS2, SPTAN1, STAC, STAC3, STAM, TEC, TP53BP2, TRIO, TRIP10, VAV1 |
| FXa_inhibition | 12 | 2.65 | 0.002581273 | ASTN1, ASTN2, CD248, F9, FBN1, GAS6, LDLR, LRP1B, LRP5, MATN3, PROS1, TLL1 |
| Ldl_recept_a | 24 | 2.64 | 1.60378E-19 | C6, C9, CFI, CORIN, DGCR2, HSPG2, LDLR, LDLRAD3, LRP1, LRP10, LRP12, LRP1B, LRP2, LRP3, LRP4, LRP6, MAMDC4, RXFP2, SORL1, SPINT1, TMPRSS15, TMPRSS6, TMPRSS7, VLDLR |
| C1_1 | 20 | 2.64 | 1.49391E-08 | CDC42BPA, CDC42BPB, DGKA, DGKB, DGKE, DGKI, DGKK, PRKCA, PRKCB, PRKCD, PRKCH, PRKCQ, PRKD1, RASGRP1, RASGRP2, RASGRP3, RASSF5, UNC13C, VAV1, VAV3 |
| Sushi | 41 | 2.63 | 4.52377E-74* | APOH, BCAN, C1R, C1S, C4BPA, C6, C7, CD46, CD55, CFH, CFHR1, CFHR2, CFHR3, CFHR4, CFHR5, CR1, CR1L, CR2, CSMD1, CSMD2, CSMD3, ENSG00000165124, F13B, GABBR1, IL2RA, KIAA0247, MASP1, MASP2, NCAN, PAMR1, PAPPA, SELE, SELL, SEZ6, SEZ6L, SEZ6L2, SRPX, SRPX2, SUSD4, SVEP1, VCAN |
| ARID | 9 | 2.62 | 4.74537E-09 | ARID1A, ARID1B, ARID2, ARID3C, ARID4B, ARID5B, JARID2, KDM5B, KDM5C |
| RRM_6 | 7 | 2.61 | 0.000103883 | FBN1, FBN2, FBN3, LTBP1, LTBP2, LTBP3, LTBP4 |
| TB | 26 | 2.61 | 2.59303E-19 | A1CF, ESRP1, ESRP2, EWSR1, HNRNPA3, HNRNPF, HNRNPH1, HNRNPH2, HNRPF, HNRPH1, IGF2BP1, IGF2BP3, PRR8, PTBP1, PTBP2, RAVER2, RBM10, RBM12, RBM12B, RBM26, RBM28, RBM33, RBM5, RBM6, TDRD10, U2AF2 |
| Chromo | 18 | 2.59 | 3.6313E-08 | CBX1, CBX3, CBX4, CBX5, CDY2A, CDY2B, CDYL, CDYL2, CHD1, CHD2, CHD3, CHD4, CHD5, CHD6, CHD7, CHD8, CHD9, MPHOSPH8 |
| Ank_4 | 15 | 2.59 | 0.000385226 | ANK3, ANKHD1, ANKRD16, ANKRD30A, ANKRD36C, ANKRD39, ANKRD44, ANKRD5, ANKS6, ASB13, ENSG00000158185, ENSG00000174501, ILK, NOTCH1, RIPK4 |
| Plectin | 5 | 2.55 | 1.08915E-06 | DSP, DST, EVPL, MACF1, PLEC |
| Ldl_recept_b | 13 | 2.51 | 7.84249E-08 | EGF, LDLR, LRP1, LRP1B, LRP2, LRP4, LRP5, LRP6, LRP8, NID1, NID2, SORL1, VLDLR |
| VWC | 19 | 2.48 | 1.82723E-11 | CHRD, CHRDL1, CHRDL2, COL2A1, COL3A1, COL5A2, CRIM1, CYR61, FRAS1, MUC5AC, MUC5B, NELL1, NELL2, PXDNL, THBS1, VWC2, VWCE, VWF, WISP1 |
| Runt | 3 | 2.46 | 0.003254998 | RUNX2, RUNX1, RUNX3 |
| SH3_2 | 3 | 2.43 | 2.22985E-05 | PHF20, PHF20L1, Q86U89 |
| DUF3776 | 26 | 2.43 | 1.01011E-09 | ARHGEF7, CASKIN2, DLG2, DOCK2, DOCK3, FYB, ITSN2, MPP5, MPP6, MPP7, OTOR, PIK3R1, RIMBP2, RIMBP3, RIMBP3B, RIMBP3C, SAMSN1, SASH3, SH3BP4, SORBS1, STAC, STAC3, TJP1, VAV2, VAV3, ZDHHC6 |
| EF-hand_8 | 17 | 2.42 | 0.003065515 | CABP7, CALM1, CALM2, CALM3, CETN2, CETN3, EFCAB6, EFHA2, GUCA1A, KCNIP2, KCNIP3, LPCAT1, MYL12A, MYL2, RYR1, SLC25A25, TNNC2 |
| Kelch_1 | 40 | 2.38 | 7.15084E-21 | CCIN, ENC1, GAN, HCFC1, IPP, IVNS1ABP, KBTBD10, KBTBD12, KBTBD3, KBTBD6, KBTBD8, KEAP1, KLHDC10, KLHDC6, KLHDC7A, KLHDC7B, KLHDC8B, KLHL1, KLHL12, KLHL13, KLHL14, KLHL15, KLHL17, KLHL18, KLHL22, KLHL23, KLHL24, KLHL25, KLHL26, KLHL28, KLHL3, KLHL31, KLHL32, KLHL33, KLHL36, KLHL4, KLHL5, KLHL6, KLHL8, KLHL9 |
| MH2 | 7 | 2.38 | 3.5307E-10 | SMAD1, SMAD2, SMAD3, SMAD4, SMAD5, SMAD7, SMAD9 |
| IL8 | 20 | 2.37 | 1.78627E-05 | CCL11, CCL13, CCL15, CCL18, CCL19, CCL2, CCL20, CCL21, CCL23, CCL24, CCL4L1, CCL4L2, CCL8, CXCL12, CXCL14, CXCL5, CXCL9, IL8, PPBP, XCL1 |
| DSPc | 30 | 2.31 | 7.10057E-26 | CDC14B, DUPD1, DUSP10, DUSP11, DUSP12, DUSP13, DUSP14, DUSP16, DUSP18, DUSP22, DUSP26, DUSP27, DUSP28, DUSP4, DUSP6, DUSP7, DUSP8, DUSP9, ENSG00000197585, EPM2A, PTEN, Q5JV89, RNGTT, SSH1, SSH2, SSH3, STYX, STYXL1, TPTE, TPTE2 |
| zf-HC5HC2H | 1 | 2.31 | 0.005845364 | FLG |
| Filaggrin | 11 | 2.31 | 2.62023E-06 | G2E3, KMT2D, MLL, MLL2, MLL3, MLL4, PHF11, PHF6, PHF7, RAI1, TCF20 |
| LRR_4 | 21 | 2.27 | 0.000106445 | CD180, CIITA, ECM2, FLII, LRRC1, LRRC2, LRRC30, LRRC39, LRRC47, LRRC56, LRRC58, LRRC67, LRRC7, LRRC8E, LRRCC1, LRRD1, LRSAM1, PHLPP2, PPP1R7, SHOC2, TLR8 |
| CARD | 18 | 2.25 | 3.63947E-07 | BCL10, BIRC2, BIRC3, CARD11, CARD14, CARD16, CARD18, CARD8, CASP1, CASP4, CASP5, IFIH1, NLRC4, NLRP1, NOD1, NOD2, PYCARD, RIPK2 |
| PSI | 16 | 2.25 | 0.00216755 | ATRNL1, EGFL4, MEGF8, MET, PLXNA1, PLXNA2, PLXNA3, PLXNA4, Q8NAP5, SEMA3G, SEMA4F, SEMA4G, SEMA5B, SEMA6C, SEMA6D, SEMA7A |
| SH3_9 | 37 | 2.23 | 1.19238E-09 | ABI2, AMPH, ARHGAP10, ARHGEF5, ARHGEF6, ASAP2, BIN1, CD2AP, DNMBP, DOCK1, FUT8, ITSN1, ITSN2, LASP1, MYO1F, NCK1, PACSIN1, PACSIN2, PACSIN3, PPP1R13L, PSTPIP1, RIMBP2, SH3BP4, SH3D19, SH3GL2, SH3GL3, SH3GLB2, SH3KBP1, SH3RF1, SH3RF2, SH3TC2, SH3YL1, SKAP2, SORBS2, SORBS3, SPATA13, STAC2 |
| FERM_C | 18 | 2.22 | 4.27735E-09 | EPB41L2, EPB41L3, EPB41L4A, EPB41L5, EZR, FARP2, FRMD3, FRMD4A, FRMD4B, FRMD5, FRMD6, FRMPD2, MSN, NF2, PTPN14, PTPN3, PTPN4, RDX |
| dsrm | 13 | 2.20 | 2.53003E-06 | ADAD1, ADAR, ADARB2, DGCR8, DHX9, DROSHA, DUS2L, EIF2AK2, PRKRA, SLC4A1AP, STAU1, STAU2, TARBP2 |
| RCC1 | 8 | 2.18 | 0.000732223 | C13orf27, LOC93081, TEX30, ABHD16A, ABHD12B, ABHD12, KIAA1310, KANSL3 |
| bZIP_1 | 16 | 2.18 | 1.81534E-08 | ALS2, HERC1, HERC2, HERC3, HERC4, HERC5, HERC6, IBTK, NEK8, NEK9, RCBTB2, RCC1, RCC2, RPGR, SERGEF, WBSCR16 |
| Abhydrolase_5 | 15 | 2.18 | 0.031855704 | ATF1, ATF2, ATF4, ATF6, ATF7, BATF, BATF2, BATF3, CREB1, CREB5, FOS, FOSB, FOSL2, JDP2, JUN |
| HLH | 17 | 2.17 | 0.01273651 | ANKS1A, ASZ1, DLC1, EPHA3, EPHA7, LRSAM1, PPFIA2, PPFIBP1, PPP1R9A, SAMD12, SAMD14, SAMD15, SAMSN1, SARM1, TP63, TP73, WDSUB1 |
| SAM_2 | 44 | 2.17 | 1.73057E-09 | ARNT, ARNT2, ARNTL, ARNTL2, ASCL1, ATOH1, BHLHA15, BHLHE41, CLOCK, FERD3L, FIGLA, HES1, HEYL, ID3, MAX, MESP2, MGA, MITF, MLX, MLXIP, MXD1, MXD3, MXI1, MYCL1, MYCN, MYF5, MYF6, MYOD1, NEUROD2, NEUROD4, NEUROD6, NEUROG2, NHLH1, OLIG3, SIM1, SREBF2, TAL1, TCF12, TCF21, TCF4, TFE3, TFEB, TFEC, TWIST1 |
| C1-set | 15 | 2.13 | 1.32074E-06 | AZGP1, B2M, CD1A, CD1B, CD1C, CD1D, CD1E, HFE, HHLA2, MR1, NCR3LG1, SIRPA, SIRPB1, SIRPG, TAPBP |
| SH2 | 14 | 2.09 | 0.000797453 | ARNT, ARNT2, ARNTL, ARNTL2, CLOCK, HIF1A, HIF3A, NCOA1, NCOA2, NCOA3, NPAS2, NPAS3, PDE8A, SIM2 |
| PAS | 64 | 2.09 | 1.82264E-21 | BCAR3, BLK, BTK, CHN1, CHN2, CLNK, CRKL, DAPP1, FER, FRK, FYN, GRAP2, GRAPL, GRB10, GRB14, hCG, HCK, INPP5D, INPPL1, JAK2, LCP2, NCK1, NCK2, PIK3R1, PIK3R2, PIK3R3, PLCG2, PTPN11, RASA1, SH2B1, SH2B2, SH2B3, SH2D1A, SH2D4A, SH2D4B, SH2D7, SHB, SHC3, SHC4, SHD, SHE, SLA, SLA2, SOCS2, SOCS3, SOCS4, STAP1, STAT1, STAT2, STAT3, STAT5B, STAT6, SYK, TEC, TENC1, TNS1, TNS3, TNS4, TXK, VAV1, VAV2, VAV3, YES1, ZAP70 |
| LRR_8 | 106 | 2.08 | 1.65248E-46 | AMIGO2, AMIGO3, ASPN, B7, BGN, CHAD, CHADL, CNTRL, DCN, ECM2, ELFN2, EPYC, ERBB2IP, FLII, FLRT2, FLRT3, FMOD, FSHR, GPR124, GPR125, IGFALS, IGSF10, ISLR, ISLR2, KERA, LGI1, LGI2, LGR4, LGR5, LGR6, LINGO2, LINGO4, LRCH1, LRCH2, LRFN2, LRFN4, LRFN5, LRG1, LRIG1, LRIG2, LRIG3, LRIT1, LRIT2, LRIT3, LRRC1, LRRC15, LRRC18, LRRC23, LRRC24, LRRC28, LRRC32, LRRC39, LRRC4, LRRC40, LRRC55, LRRC66, LRRC70, LRRC8B, LRRC8D, LRRIQ4, LRRK2, LRRN1, LRRN2, LRRN3, LRRTM1, LRRTM2, LRRTM3, LRRTM4, LRTM1, LUM, MXRA5, OMD, PHLPP, PHLPP1, PIDD, PODN, PODNL1, PRELP, PXDN, PXDNL, Q7Z2Q7, RSU1, RTN4RL1, RXFP1, RXFP2, SCRIB, SHOC2, SLIT1, SLIT2, SLIT3, SLITRK1, SLITRK2, SLITRK3, SLITRK4, SLITRK6, TLR1, TLR10, TLR3, TLR4, TLR5, TLR6, TLR7, TLR9, TPBG, TSHR, VASN |
| FAM47 | 7 | 2.08 | 3.34755E-19 | ENSG00000185448, FAM47A, FAM47B, FAM47C, FAM47E, LOC442444, Q6ZV65 |
| PWWP | 13 | 2.08 | 0.000107105 | BRPF3, DNMT3A, DNMT3B, HDGF, HDGFRP3, MUM1L1, NSD1, PSIP1, PWWP2B, WHSC1, WHSC1L1, ZCWPW1, ZCWPW2 |
| Disintegrin | 15 | 2.06 | 0.020773995 | ADAM10, ADAM12, ADAM18, ADAM2, ADAM20, ADAM21, ADAM22, ADAM23, ADAM29, ADAM30, ADAM32, ADAM33, ADAM7, ADAM8, ADAM9 |
| Pro-rich | 6 | 2.05 | 1.27112E-06 | PRB1, PRB2, PRB3, PRB4, PRH2, PRR4 |
| zf-RING_2 | 26 | 2.04 | 0.035611689 | DTX3, DTX4, LONRF2, MYCBP2, PDZRN4, RBCK1, RNF11, RNF111, RNF115, RNF126, RNF13, RNF139, RNF148, RNF149, RNF165, RNF175, RNF181, RNF215, RNF25, RNF32, RNF43, RNF44, RNF6, SCAF11, TRIM59, ZNRF3 |
| Gelsolin | 11 | 2.04 | 0.00692656 | AVIL, CAPG, GSN, SCIN, SEC23A, SEC24A, SEC24B, SEC24C, SEC24D, VIL1, VILL |
| JmjC | 10 | 2.03 | 0.03012614 | ARHGAP4, FCHO2, FCHSD1, FER, FES, FNBP1L, GAS7, PACSIN3, SRGAP1, SRGAP3 |
| FCH | 14 | 2.03 | 7.9483E-06 | KDM6A, KDM4E, KDM4C, KDM5C, KDM5B, KDM3A, KDM4B, UTY, KDM3B, KDM6B, HR, KDM4A, PHF8, KDM2A |
| I-set | 25 | 2.02 | 5.01162E-16* | EGFL4, HSPG2, LAMA1, LAMA2, LAMA3, LAMA4, LAMA5, LAMB1, LAMB2, LAMB3, LAMB4, LAMC1, LAMC2, LAMC3, MEGF10, MEGF11, MEGF6, MEGF8, NTN1, NTN4, NTN5, NTNG1, NTNG2, PEAR1, USH2A |
| Laminin_EGF | 113 | 2.02 | 9.5218E-150 | ADAMTSL1, ADAMTSL3, ALPK3, BOC, BSG, CADM2, CCDC141, CDON, CHL1, CNTN1, CNTN2, CNTN3, CNTN4, CNTN5, CNTN6, DCC, DSCAM, DSCAML1, EMB, FGFR1, FGFR2, FGFR3, FGFR4, FGFRL1, FLT1, FLT4, HEPACAM2, HMCN1, HSPG2, IGDCC3, IGFBP7, IGFBPL1, IGFN1, IGSF10, IGSF5, IGSF9, IGSF9B, IL1RAPL1, IL1RL1, JAM2, KDR, KIRREL2, KIRREL3, L1CAM, LINGO4, LRFN5, LRIG1, LRIG3, LRIT2, LRIT3, LRRC4B, LRRC4C, LRRN2, LRRN3, LSAMP, MAG, MDGA1, MDGA2, MERTK, MUSK, MXRA5, MYBPC1, MYBPC2, MYBPC3, MYLK, MYOM1, MYOM2, MYOT, MYPN, NCAM2, NEGR1, NEXN, NFASC, NPHS1, NPTN, NRCAM, NRG1, NTM, NTRK2, OBSCN, OBSL1, OPCML, PALLD, PDGFRA, PDGFRL, PRTG, PTK7, PTPRD, PTPRS, PXDN, PXDNL, Q7Z2S2, ROBO1, ROBO2, ROBO3, ROBO4, ROR1, ROR2, SDK1, SDK2, SEMA3C, SIGLEC1, SIGLEC10, SIGLEC12, SIGLEC6, SPEG, TMIGD1, TTN, TYRO3, UNC5A, UNC5D, VCAM1, VSIG10 |
| CTNNB1_binding | 4 | 1.99 | 5.70677E-05 | LEF1, TCF7, TCF7L1, TCF7L2 |
| MH1 | 9 | 1.98 | 0.006764796 | SMAD4, NFIA, SMAD2, SMAD3, SMAD7, SMAD9, NFIB, SMAD5, SMAD1 |
| Cadherin | 110 | 1.96 | 3.03631E-30* | A1CF, CELF1, CELF2, CELF4, CELF6, CIRBP, CPEB4, CSTF2, CSTF2T, DAZAP1, DAZL, DND1, EIF4B, ELAVL1, ELAVL2, ELAVL3, ELAVL4, ENOX1, ENOX2, ENSG00000106232, ENSG00000176757, ENSG00000187999, FLJ20273, FUS, G3BP2, HNRNPA1, HNRNPA1L2, HNRNPA1P16, HNRNPA2B1, HNRNPA3, HNRNPAB, HNRNPC, HNRNPD, HNRNPF, HNRNPM, HNRNPR, HNRPD, HNRPDL, HNRPF, HNRPR, IGF2BP1, IGF2BP2, IGF2BP3, LOC120364, MKI67IP, MYEF2, NCL, NONO, PABPC1, PABPC1L, PABPC3, PABPC4, PABPC4L, PABPC5, POLDIP3, PPARGC1B, PPIE, PPRC1, PTBP1, PTBP3, RALY, RBFOX1, RBFOX2, RBM14, RBM15, RBM18, RBM19, RBM23, RBM28, RBM3, RBM38, RBM39, RBM4, RBM42, RBM44, RBM45, RBM46, RBM47, RBM7, RBMS3, RBMX, RDM1, ROD1, SAFB2, SCAF4, SCAF8, SF3B4, SFPQ, SLIRP, SLTM, SNRNP35, SNRNP70, SNRPA, SPEN, SRSF1, SRSF11, SRSF12, SRSF4, SRSF5, SRSF6, SSB, SYNCRIP, TAF15, TIA1, TIAL1, TRA2A, TRA2B, TRNAU1AP, U2AF1L4, U2AF2 |
| RRM_1 | 108 | 1.96 | 2.6729E-117 | CDH1, CDH10, CDH11, CDH12, CDH13, CDH15, CDH16, CDH17, CDH18, CDH19, CDH2, CDH20, CDH22, CDH24, CDH26, CDH3, CDH4, CDH5, CDH6, CDH7, CDH8, CDH9, CDHR1, CDHR2, CDHR3, CELSR1, CELSR2, CELSR3, CLSTN1, CLSTN2, DCHS1, DCHS2, DSC1, DSC2, DSC3, DSG1, DSG3, DSG4, FAT, FAT1, FAT2, FAT3, FAT4, PCDH1, PCDH10, PCDH11X, PCDH11Y, PCDH12, PCDH15, PCDH17, PCDH18, PCDH19, PCDH20, PCDH7, PCDH8, PCDH9, PCDHA1, PCDHA10, PCDHA11, PCDHA12, PCDHA13, PCDHA2, PCDHA3, PCDHA4, PCDHA5, PCDHA6, PCDHA7, PCDHA8, PCDHA9, PCDHAC1, PCDHAC2, PCDHB1, PCDHB10, PCDHB11, PCDHB12, PCDHB13, PCDHB14, PCDHB15, PCDHB16, PCDHB18, PCDHB2, PCDHB3, PCDHB4, PCDHB5, PCDHB6, PCDHB7, PCDHB8, PCDHG, PCDHGA1, PCDHGA10, PCDHGA11, PCDHGA12, PCDHGA2, PCDHGA3, PCDHGA4, PCDHGA5, PCDHGA6, PCDHGA7, PCDHGA8, PCDHGA9, PCDHGB1, PCDHGB2, PCDHGB3, PCDHGB4, PCDHGB6, PCDHGB7, PCDHGC4, PCDHGC5 |
| zf-C4 | 26 | 1.94 | 0.000103765 | ESR1, ESRRG, HNF4A, HNF4G, NR1D2, NR1H3, NR1H4, NR1I2, NR1I3, NR2E1, NR2F2, NR3C1, NR3C2, NR4A2, NR5A2, PGR, Q8N8C9, RARB, RARG, RORA, RORC, RXRA, RXRG, THRA, THRB, VDR |
| PYRIN | 18 | 1.94 | 0.004084803 | AIM2, IFI16, MEFV, MNDA, NLRP1, NLRP10, NLRP11, NLRP12, NLRP13, NLRP14, NLRP2, NLRP3, NLRP4, NLRP5, NLRP7, NLRP8, NLRP9, PYHIN1 |
| RasGAP | 11 | 1.90 | 5.37275E-06 | DAB2IP, GAPVD1, IQGAP2, IQGAP3, NF1, RASA1, RASA2, RASA3, RASAL1, RASAL2, SYNGAP1 |
| LIM | 46 | 1.88 | 6.24671E-09 | ABLIM1, ABLIM2, ABLIM3, C6orf49, CSRP1, CSRP2, CSRP3, FBLIM1, FHL1, FHL3, FHL5, LASP1, LDB3, LHX3, LHX4, LHX5, LHX8, LHX9, LIMCH1, LIMD1, LIMD2, LIMK2, LIMS1, LIMS2, LIMS3, LMCD1, LMO2, LMO4, LMX1B, LPP, LPXN, MICAL1, MICALL2, NRAP, PDLIM2, PDLIM4, PDLIM5, PDLIM7, PRICKLE2, PRICKLE3, PRICKLE4, TES, TGFB1I1, TRIP6, WTIP, ZYX |
| fn3 | 119 | 1.85 | 9.7436E-98* | ANKFN1, AXL, BOC, CDON, CHL1, CMYA5, CNTFR, CNTN1, CNTN2, CNTN3, CNTN4, CNTN5, CNTN6, COL12A1, COL14A1, COL20A1, COL7A1, CRLF1, CSF2RB, DCC, DSCAM, DSCAML1, EBI3, EGFLAM, ENSG00000188974, EPHA1, EPHA10, EPHA2, EPHA3, EPHA4, EPHA5, EPHA6, EPHA7, EPHA8, EPHB1, EPHB2, EPHB3, EPHB6, FLRT2, FN1, FNDC1, FNDC3A, FNDC3B, FNDC5, FNDC7, FSD1, FSD1L, FSD2, GHR, IGDCC4, IGF1R, IGFN1, IGSF22, IGSF9, IGSF9B, IL12RB2, IL27RA, IL2RG, IL31RA, IL6ST, IL7R, INSR, INSRR, ITGB4, KAL1, L1CAM, LIFR, LRFN2, LRFN4, LRRN3, LRRN4CL, MID1, MPL, MYBPC1, MYBPC2, MYBPC3, MYBPH, MYLK, MYOM1, MYOM2, MYOM3, NCAM2, NEO1, NFASC, NPHS1, NRCAM, OBSCN, OSMR, PHYHIPL, PRTG, PTPRB, PTPRC, PTPRD, PTPRF, PTPRH, PTPRK, PTPRM, PTPRS, PTPRT, ROBO1, ROBO2, ROBO3, ROBO4, ROS1, SDK1, SDK2, SNED1, SORL1, TEK, TIE1, TNC, TNN, TNR, TNXB, TRIM36, TRIM42, TTN, TYRO3, USH2A |
| Ephrin_lbd | 14 | 1.83 | 2.73766E-05 | ENSG00000188974, EPHA1, EPHA10, EPHA2, EPHA3, EPHA4, EPHA5, EPHA6, EPHA7, EPHA8, EPHB1, EPHB2, EPHB3, EPHB6 |
| Homeobox | 108 | 1.82 | 2.45383E-13 | ADNP, ALX1, ALX3, ALX4, ARGFX, BARHL2, BARX2, BSX, CDX1, CDX4, CRX, CUX1, DBX2, DLX3, DLX4, DLX5, DUXA, EMX2, EN1, EVX2, GBX1, GBX2, GSC2, GSX2, HESX1, HHEX, HMX2, HMX3, HNF1A, HNF1B, HOXA10, HOXA11, HOXA13, HOXA2, HOXA3, HOXA4, HOXA6, HOXB1, HOXB2, HOXB5, HOXB7, HOXB9, HOXC5, HOXC8, HOXC9, HOXD1, HOXD12, HOXD13, HOXD3, HOXD4, HOXD8, ISL1, ISL2, LASS3, LASS4, LASS5, LBX1, LBX2, LEUTX, LHX2, LHX3, LHX4, LHX6, LMX1A, MEOX1, MEOX2, MNX1, MSX2, NANOG, NOBOX, ONECUT1, OTP, OTX2, PAX6, PAX7, PBX4, PITX2, POU1F1, POU2F2, POU2F3, POU3F1, POU3F2, POU3F3, POU3F4, POU4F1, POU5F1B, POU6F1, RHOXF1, RHOXF2, SATB1, SATB2, SEBOX, SHOX2, SIX1, SIX2, SIX3, SIX4, SIX6, TLX2, UNCX, VAX2, VSX1, ZEB1, ZFHX3, ZFHX4, ZHX1, ZHX2, ZHX3 |
| SCAN | 34 | 1.81 | 7.4818E-05 | PEG3, ZIM2, ZKSCAN1, ZKSCAN3, ZKSCAN4, ZNF174, ZNF192, ZNF193, ZNF197, ZNF213, ZNF215, ZNF232, ZNF24, ZNF274, ZNF287, ZNF323, ZNF394, ZNF397, ZNF444, ZNF445, ZNF449, ZNF483, ZNF498, ZNF500, ZNF75D, ZSCAN1, ZSCAN18, ZSCAN20, ZSCAN21, ZSCAN22, ZSCAN23, ZSCAN29, ZSCAN4, ZSCAN5B |
| Collagen | 10 | 1.79 | 1.84625E-07 | FLNA, FLNB, FLNC, KDELC1, KDELC2, KIAA0317, MYCBP2, TRIM2, TRIM45, TRIM71 |
| CUB | 45 | 1.79 | 1.57838E-31 | ATRN, ATRNL1, BMP1, C1R, C1RL, C1S, CDCP2, CSMD1, CSMD2, CSMD3, CUBN, CUZD1, DCBLD1, DCBLD2, DMBT1, EGFL4, GPR126, KREMEN1, LRP10, LRP12, LRP3, MASP1, MASP2, MEGF8, MFRP, NETO1, NETO2, NRP1, NRP2, OVCH1, OVCH2, PAMR1, PCOLCE, PCOLCE2, PDGFC, PDGFD, SCUBE1, SCUBE3, SEZ6L, SEZ6L2, ST14, TLL1, TLL2, TMPRSS15, TMPRSS7 |
| Filamin | 67 | 1.79 | 1.92062E-38 | C1QA, C1QB, C1QC, C1QTNF1, C1QTNF3, C1QTNF7, C1QTNF8, C1QTNF9, C1QTNF9B, CCBE1, COL10A1, COL11A1, COL11A2, COL12A1, COL13A1, COL14A1, COL15A1, COL16A1, COL17A1, COL18A1, COL19A1, COL1A1, COL1A2, COL20A1, COL21A1, COL22A1, COL23A1, COL24A1, COL25A1, COL27A1, COL28A1, COL2A1, COL3A1, COL4A1, COL4A2, COL4A3, COL4A4, COL4A5, COL4A6, COL5A1, COL5A2, COL5A3, COL6A1, COL6A2, COL6A5, COL6A6, COL7A1, COL8A1, COL8A2, COL9A1, COL9A2, COL9A3, COLEC10, COLEC11, COLEC12, COLQ, CTHRC1, EMID1, EMID2, FCN1, FLJ35880, MSR1, OTOL1, SCARA3, SCARA5, SFTPD, WDR33 |
| Cadherin_2 | 39 | 1.78 | 2.85426E-05 | PCDH10, PCDH11X, PCDH11Y, PCDH18, PCDH19, PCDH8, PCDH9, PCDHA11, PCDHA13, PCDHA2, PCDHA3, PCDHA7, PCDHA8, PCDHAC1, PCDHAC2, PCDHB1, PCDHB10, PCDHB11, PCDHB12, PCDHB14, PCDHB16, PCDHB2, PCDHB3, PCDHB4, PCDHB5, PCDHB6, PCDHB7, PCDHG, PCDHGA10, PCDHGA11, PCDHGA2, PCDHGA3, PCDHGA5, PCDHGA6, PCDHGA8, PCDHGB1, PCDHGB2, PCDHGB6, PCDHGC5 |
| SRCR | 19 | 1.76 | 0.000896697 | ASCC1, BICC1, FUBP1, FUBP3, FXR1, FXR2, HDLBP, HNRNPK, IGF2BP1, IGF2BP2, IGF2BP3, KHDRBS2, KHSRP, MEX3A, NOVA1, PCBP2, PCBP3, QKI, SF1 |
| KH_1 | 16 | 1.76 | 0.017042056 | FGF12, FGF5, FGF6, FGF13, FGF9, FGF10, FGF7,FGF8, FGF14, FGF19, FGF23, FGF4, FGF18, FGF22, FGF16, FGF1 |
| FGF | 15 | 1.76 | 5.39942E-09 | CD163, CD163L1, CD5, CD5L, CD6, DMBT1, LOXL2, LOXL3, LOXL4, MARCO, MSR1, PRSS12, SCARA5, SRCRB4D, SSC5D |
| TPR_11 | 41 | 1.75 | 3.17368E-05 | BBS4, CDC27, ENSG00000204815, FKBPL, GTF3C3, IFIT2, IFIT5, KDM6A, LONRF2, NAA16, OGT, PEX5, PEX5L, PPID, SGTB, SMYD4, SPAG1, TANC1, TANC2, TMTC1, TMTC2, TMTC4, TOMM34, TOMM70A, TTC12, TTC14, TTC16, TTC18, TTC21B, TTC25, TTC27, TTC3, TTC30A, TTC33, TTC37, TTC3P1, TTC4, TTC6, TTC9C, WDTC1, ZC3H7A |
| C2 | 87 | 1.74 | 3.12541E-21 | C2CD3, C2CD4D, CAPN5, CAPN6, CC2D1A, CC2D1B, CCDC33, CPNE1, CPNE3, CPNE4, CPNE6, CPNE7, CPNE8, CPNE9, DOC2A, DYSF, ENSG00000189290, ESYT1, ESYT2, ESYT3, FER1L5, FER1L6, HECW2, ITCH, ITSN2, KIAA0528, MCTP1, MCTP2, MTAC2D1, MYOF, OTOF, PCLO, PIK3C2A, PIK3C2B, PLA2G4A, PLA2G4E, PLCB2, PLCB3, PLCB4, PLCE1, PLCH1, PLCL2, PLCZ1, PRF1, PRKCA, PRKCB, PRKCG, RAB11FIP1, RAB11FIP5, RASA1, RASA2, RASA3, RASAL1, RASAL2, RGS3, RIMS1, RIMS2, RPGRIP1L, RPH3A, SMURF2, SYNGAP1, SYT1, SYT10, SYT11, SYT14, SYT15, SYT16, SYT17, SYT2, SYT4, SYT5, SYT6, SYT7, SYT8, SYT9, SYTL1, SYTL2, SYTL3, SYTL4, SYTL5, TC2N, TOLLIP, UNC13A, UNC13B, UNC13C, UNC13D, WWP1 |
| Peptidase_C14 | 11 | 1.73 | 2.12265E-05 | CASP1, CASP10, CASP14, CASP2, CASP3, CASP4, CASP5, CASP6, CASP7, CASP8, MALT1 |
| cNMP_binding | 25 | 1.72 | 0.000861284 | CNBD1, CNGA1, CNGA2, CNGA4, CNGB1, CNGB3, HCN1, HCN2, KCNH1, KCNH5, KCNH6, KCNH7, KCNH8, PNPLA7, PRKAR1A, PRKAR1B, PRKAR2A, PRKAR2B, PRKG1, PRKG2, RAPGEF3, RAPGEF4, RAPGEF6, SLC9A10, SLC9A11 |
| Kringle | 15 | 1.67 | 0.000736988 | LPA, PLG, HGF, ROR2, LPAL2, HABP2, MST1, ROR1, HGFAC, PLAU, Q13209, Q49A61, MST1P9, F2, PLAT |
| Spectrin | 23 | 1.66 | 1.04836E-27 | ACTN1, ACTN2, ACTN4, AKAP6, C14orf49, DMD, DRP2, DST, KALRN, MACF1, SPTA1, SPTAN1, SPTB, SPTBN1, SPTBN2, SPTBN4, SPTBN5, SYNE1, SYNE2, SYNE3, TRAD, TRIO, UTRN |
| PDZ | 71 | 1.63 | 2.7485E-11 | APBA1, APBA2, ARHGEF11, CASK, CNKSR3, DFNB31, DLG1, DLG2, DLG3, ERBB2IP, FRMPD1, GIPC2, GIPC3, GRASP, GRIP1, HTRA1, IL16, INADL, LIMK2, LIN7A, LIN7C, LMO7, LNX1, LNX2, LRRC7, MAGI1, MAGI2, MAST1, MAST2, MAST3, MLLT4, MPDZ, MPP3, MPP5, MPP6, MPP7, NOS1, PARD3, PARD3B, PARD6A, PDZD3, PDZD4, PDZD9, PDZK1, PDZRN3, PDZRN4, PICK1, PREX2, PTPN13, PTPN3, RAPGEF6, RGS12, RGS3, RIMS1, RIMS2, SCRIB, SDCBP2, SHANK1, SHANK2, SHROOM4, SNTG1, SNX27, SYNJ2BP, SYNPO2, TAX1BP3, TIAM1, TIAM2, TJP1, TJP2, TJP3, USH1C |
| EF-hand_7 | 53 | 1.60 | 0.002021875 | CABP4, CABP5, CABP7, CALB1, CALB2, CALM1, CALM2, CALM3, CALML5, CALN1, CAPS2, CAPSL, CETN1, CETN2, CETN3, CIB3, DGKB, DST, EFCAB1, EFCAB11, EFCAB6, EFHD1, EFHD2, FKBP14, GUCA1A, GUCA1B, GUCA1C, HPCAL4, KCNIP1, KCNIP2, KCNIP3, KCNIP4, LPCAT2, MYL10, NCS1, NECAB2, NUCB1, NUCB2, OCM, OCM2, PLCH1, PLS3, PPP2R3A, PPP2R3B, PPP3R1, PPP3R2, RCN2, RCVRN, RHBDL3, SLC25A23, SLC25A25, SPTAN1, SRI |
| Ig_2 | 127 | 1.58 | 2.26627E-19 | A1BG, ADAMTSL3, AGER, ALCAM, AMIGO2, BCAM, BOC, BSG, BTLA, CADM3, CADM4, CD101, CD22, CD226, CD244, CD48, CD86, CD96, CEACAM16, CEACAM21, CEACAM7, CILP, CILP2, CNTN1, CNTN2, CNTN3, CNTN4, CXADR, DCC, DSCAM, DSCAML1, ENSG00000197865, FCAR, FCER1A, FCGR2A, FCGR2B, FCRL1, FCRL2, FCRL3, FCRL4, FCRL5, FCRL6, FCRLA, FCRLB, FGFR2, FLT1, FLT4, FSTL5, GP6, GPA33, GPR116, HEPACAM, HSPG2, ICAM1, ICAM3, ICAM5, IGLON5, IGSF1, IGSF21, IGSF3, IGSF5, IGSF9, IGSF9B, IL18R1, IL18RAP, IL1R2, IL1RAP, IL1RAPL1, IL1RAPL2, IL1RL1, ISLR2, KDR, KIR2DL1, KIR2DL4, KIR3DX1, KIRREL, KIRREL3, L1CAM, LAIR2, LILRA1, LILRA2, LILRA3, LILRA4, LILRA5, LILRA6, LILRB1, LILRB2, LILRB3, LILRB4, LILRB5, MALT1, MDGA1, MDGA2, MUSK, MXRA5, MYOM2, MYOM3, NFASC, NPHS1, NRCAM, PDGFRB, PSG1, PSG11, PSG2, PSG3, PSG4, PSG6, PSG8, PSG9, PTGFRN, PTK7, PTPRK, PVRL1, PVRL3, SDK1, SIGLEC1, SIGLEC10, SIGLEC12, SIGLEC14, SIGLEC5, SIGLEC8, TIE1, TTN, VCAM1, VSIG1, VSIG10L, VSTM1 |
| Laminin_G_2 | 34 | 1.47 | 2.94677E-06 | CELSR1, CELSR2, CELSR3, CNTNAP1, CNTNAP2, CNTNAP3, CNTNAP4, CNTNAP5, COL11A1, COL11A2, COL24A1, COL5A1, CRB1, CRB2, CSPG4, EGFLAM, FAT, FAT1, FAT2, FAT3, FAT4, GAS6, LAMA3, LAMA4, LAMA5, NELL1, NELL2, NRXN1, NRXN2, NRXN3, PROS1, SLIT2, SLIT3, USH2A |
| RhoGAP | 54 | 1.45 | 4.21719E-05 | ABR, AC142381, ARAP2, ARHGAP1, ARHGAP11A, ARHGAP12, ARHGAP15, ARHGAP17, ARHGAP18, ARHGAP20, ARHGAP21, ARHGAP22, ARHGAP23, ARHGAP24, ARHGAP25, ARHGAP26, ARHGAP28, ARHGAP29, ARHGAP30, ARHGAP31, ARHGAP32, ARHGAP33, ARHGAP35, ARHGAP36, ARHGAP39, ARHGAP4, ARHGAP40, ARHGAP44, ARHGAP5, ARHGAP6, ARHGAP8, C5orf5, CHN2, DLC1, ENSG00000167433, FAM13A, FAM13B, GMIP, GRLF1, HMHA1, INPP5B, LOC257106, MYO9A, OCRL, OPHN1, PIK3R1, RACGAP1, RALBP1, SH3BP1, SRGAP1, SRGAP3, STARD8, SYDE1, SYDE2 |
| Ank_2 | 129 | 1.42 | 1.97177E-12 | ABTB2, AGAP5, ANK2, ANK3, ANKAR, ANKFY1, ANKHD1, ANKIB1, ANKK1, ANKLE1, ANKMY1, ANKMY2, ANKRA2, ANKRD12, ANKRD13B, ANKRD13C, ANKRD16, ANKRD17, ANKRD18A, ANKRD18B, ANKRD2, ANKRD20A4, ANKRD22, ANKRD23, ANKRD29, ANKRD30A, ANKRD30B, ANKRD33, ANKRD34A, ANKRD34B, ANKRD34C, ANKRD35, ANKRD36, ANKRD36C, ANKRD37, ANKRD39, ANKRD40, ANKRD42, ANKRD44, ANKRD45, ANKRD46, ANKRD49, ANKRD5, ANKRD50, ANKRD52, ANKRD55, ANKRD56, ANKRD57, ANKRD60, ANKRD7, ANKS1B, ANKS6, ANKUB1, ASAP1, ASAP3, ASB10, ASB12, ASB13, ASB14, ASB15, ASB16, ASB18, ASB2, ASB5, ASB6, ASB7, ASB9, ASZ1, BCOR, BTBD11, CLIP3, CLPB, CTTNBP2, ENSG00000158185, ENSG00000174501, ENSG00000188219, ENSG00000230031, ENSG00000254809, ESPNL, FLJ43980, GABPB2, GIT1, GLS, GLS2, GPANK1, HACE1, ILK, INVS, KANK4, KIDINS220, LOC157567, LOC728378, LRRK1, MIB1, MIB2, MTMR8, MYO16, NFKBID, NOTCH1, NOTCH3, NRARP, NUDT12, OSBPL1A, POTE2, POTEA, POTEB, POTEC, POTED, POTEF, POTEG, POTEH, PPP1R13L, PPP1R16A, PPP1R16B, RAI14, RIPK4, SHANK1, SNCAIP, TANC1, TANC2, TNKS, TNKS2, TNNI3K, TRPC6, TRPC7, TRPV3, TRPV5, UACA, ZDHHC13 |
| V-set | 122 | 1.41 | 1.31881E-07 | ALCAM, AMICA1, BCAN, BTN2A1, BTN3A2, BTN3A3, BTNL2, BTNL3, BTNL8, BTNL9, C1orf204, CADM2, CADM3, CADM4, CD101, CD2, CD226, CD274, CD28, CD300A, CD300C, CD300LB, CD300LD, CD300LF, CD300LG, CD4, CD48, CD79A, CD79B, CD80, CD83, CD86, CD8A, CD8B, CEACAM1, CEACAM18, CEACAM19, CEACAM21, CEACAM3, CEACAM4, CEACAM6, CEACAM7, CEACAM8, CLMP, CRTAM, CXADR, ESAM, FCAMR, GPA33, HAPLN1, HAPLN2, HAPLN4, HAVCR1, HAVCR2, HEPACAM, HHLA2, ICOSLG, IGLON5, IGSF21, IGSF3, IGSF9B, KDR, MGC33530, MOG, MPZ, MPZL1, MPZL2, NCR2, NPHS1, PDCD1, PIGR, PILRA, PILRB, PSG1, PSG11, PSG2, PSG4, PSG5, PSG6, PSG8, PSG9, PTGFRN, PVR, PVRL3, Q5JXA8, SCN2B, SCN3B, SEMA3D, SIGLEC10, SIGLEC11, SIGLEC12, SIGLEC16, SIGLEC5, SIGLEC6, SIGLEC7, SIGLEC8, SIGLEC9, SIRPB1, SIRPB2, SIRPD, SIRPG, SLAMF6, SLAMF7, TIGIT, TIMD4, TREM1, TREM2, TREML1, TREML2, TREML4, U66061, VCAN, VPREB1, VPREB3, VSIG1, VSIG10, VSIG2, VSIG8, VSTM2A, VSTM2L, VSTM4, VSTM5 |
| Lectin_C | 68 | 1.37 | 0.0339018 | CHODL, REG3G, ATRNL1, CLEC2B, CLEC3A, REG3A, CLEC4D, KLRG1, OLR1, CLEC4M, CD207, REG4, BCAN, REG1A, CD72, KLRB1, CD69, CLEC1A, KLRK1, MRC2, PLA2R1, PKD1L2, CLEC2A, ENSG00000166473, VCAN, SELL, LY75, CLEC5A, SFTPA2B, CLEC10A, NCAN, CLEC11A, CLEC6A, KLRC2, COLEC12, SFTPA2, CLEC1B, FREM1, ENSG00000164946, AGC1, ACAN, CLEC4F, REG1B, KLRG2, MRC1, MBL2, SFTPD, PRG3, CLEC2D, CLEC9A, KLRD1, KLRC1, ASGR1, CLEC4G, MRC1L1, LAYN, CLEC18B, CD302, DGCR2, KLRF1, CLEC2L, COLEC11, CLEC4A, CD93, CLEC12B, ASGR2, CLEC4E, ATRN |
| PH | 122 | 1.30 | 0.00946844 | ABR, ACAP1, ACAP2, ACAP3, ADRBK2, AFAP1, AFAP1L1, AFAP1L2, AGAP1, AGAP2, AGAP3, AGAP6, AKAP13, AKT1, AKT3, ANLN, APBB1IP, ARAP1, ARAP2, ARAP3, ARHGAP12, ARHGAP15, ARHGAP21, ARHGAP24, ARHGAP25, ARHGAP27, ARHGEF18, ARHGEF4, ARHGEF6, ARHGEF7, ARHGEF9, ASAP2, ASAP3, BTK, CDC42BPG, CNKSR1, CNKSR2, COL4A3BP, CYTH4, DAPP1, DEF6, DGKD, DNM1, DNM2, DNM3, DOCK11, DOK4, FAM109A, FAM109B, FARP1, FARP2, FERMT2, FGD1, FGD3, FGD4, FGD5, FGD6, GAB1, GAB2, GAB4, GRB10, GRB14, IPCEF1, ITK, KIF1A, MPRIP, MYO10, NET1, OSBP2, OSBPL11, OSBPL5, OSBPL8, OSBPL9, PHLDB2, PHLDB3, PLD1, PLEK, PLEK2, PLEKHA1, PLEKHA2, PLEKHA3, PLEKHA4, PLEKHA5, PLEKHA6, PLEKHA7, PLEKHA8, PLEKHB2, PLEKHD1, PLEKHF1, PLEKHF2, PLEKHH1, PLEKHH2, PLEKHM2, PLEKHM3, PLEKHO2, PREX1, PRKD1, Q96HZ0, RALGPS1, RAPH1, RASA1, RASA2, RASGRF1, ROCK1, ROCK2, RTKN, SBF1, SH2B1, SH3D20, SKAP1, SKAP2, SNTB1, SOS1, SOS2, STAP1, SWAP70, TEC, TRIOBP, VAV1, VAV2, VAV3, VEPH1 |

The significant domains in whole genome are listed by the Pfam domain name, the number of domains, the mutation enrichment expressed as the ratio of the observed number of domain mutations to the expected number of mutation, the Bonferroni corrected p-value and the gene names. The list sorted by enrichment score followed by the number of domains.

*Indicates that the initial *p*-value was calculated using Fisher’s exact test.
